# Correlation Energy of a Weakly Interacting Fermi Gas with Large Interaction Potential

**DOI:** 10.1007/s00205-023-01893-6

**Published:** 2023-07-05

**Authors:** Niels Benedikter, Marcello Porta, Benjamin Schlein, Robert Seiringer

**Affiliations:** 1 Dipartimento di Matematica, Università degli Studi di Milano, Via Cesare Saldini 50, 20133 Milan, Italy; 2 SISSA, Mathematics Area, Via Bonomea 265, 34136 Trieste, Italy; 3 Institute of Mathematics, University of Zurich, Winterthurerstrasse 190, 8057 Zurich, Switzerland; 4 IST Austria, Am Campus 1, 3400 Klosterneuburg, Austria

## Abstract

Recently the leading order of the correlation energy of a Fermi gas in a coupled mean-field and semiclassical scaling regime has been derived, under the assumption of an interaction potential with a small norm and with compact support in Fourier space. We generalize this result to large interaction potentials, requiring only $|\cdot | \hat{V} \in \ell ^1 (\mathbb {Z}^3)$. Our proof is based on approximate, collective bosonization in three dimensions. Significant improvements compared to recent work include stronger bounds on non-bosonizable terms and more efficient control on the bosonization of the kinetic energy.

## Introduction

The interacting high-density Fermi gas models a variety of important physical systems, in particular the behavior of electrons in alkali metals. The simplest approximation for the computation of its physical properties is mean-field theory, that is, Hartree–Fock theory. Hartree–Fock theory only includes the minimal amount of quantum correlations unavoidable due to the antisymmetry requirement on the wave function of fermionic many-body systems. In the present paper we consider corrections to the Hartree–Fock energy due to non-trivial quantum correlations (that is, entanglement in the ground state).

According to [7], the dominant effect of correlations on the ground state energy should be described by the *random-phase approximation (RPA)*, which may also be formulated as a partial resummation of the perturbation series [17] or as a theory of particle–hole pairs behaving as bosonic quasiparticles [27]. The latter point of view was recently used by [4, 5] (extending the second-order result of [20]) to rigorously prove the validity of the random-phase approximation for the ground state energy, assuming the interaction potential to be small and its Fourier transform to have compact support. *In the present paper, that result is generalized to arbitrarily large interaction potentials without restriction on the support.* Our proof is a refinement of the method of [4, 5], a crucial point of which is to delocalize particle–hole pairs over patches on the Fermi surface, thus circumventing the Pauli principle and justifying the approximate bosonization of particle–hole pairs. This approach leads to a bosonic quasifree effective theory, from which the ground state energy can be computed.

The further predictions of this bosonic effective theory have been discussed in [1] and it has also been proven to be a good approximation for the time evolution of the Fermi gas [5], refining the time-dependent Hartree–Fock approximation derived in [3, 8–10]. An alternative approach to the ground state energy, avoiding delocalization and thus closer in spirit to [27] has been developed recently in [13]: still, also there an averaging over different particle–hole pairs is needed to justify the bosonization. In another context, the low-density Fermi gas, bosonization ideas have been applied by [15, 18, 19].

Let us turn to the mathematical description of our result. We consider a system of *N* fermions on the torus $\mathbb {T}^3 := \mathbb {R}^3 / (2\pi \mathbb {Z}^3)$ interacting through a potential *V*. The system is described on the Hilbert space $L^2_\text{ a } (\mathbb {T}^{3N})$, consisting of all $\psi \in L^2 (\mathbb {T}^{3N})$ that are antisymmetric under exchange of particles,

$$\begin{aligned} \psi (x_{\sigma (1)} , \dots , x_{\sigma (N)}) = \text {sgn} (\sigma ) \psi (x_1, \dots , x_N) \end{aligned}$$

for all permutations $\sigma \in \mathcal {S}_N$. The Hamiltonian is the linear self-adjoint operator

1.1 $$\begin{aligned} H_N := \sum _{j=1}^N -\hbar ^2 \Delta _{x_j} + \lambda \sum _{i<j}^N V (x_i - x_j) \;. \end{aligned}$$

The interaction potential *V* is assumed to have non-negative Fourier transform $\hat{V} \geqq 0$. (For the interaction potential we use the convention that the Fourier transform is $V(x) = \sum _{k \in \mathbb {Z}^3} \hat{V}(k) e^{ik\cdot x}$, unlike for the Fourier transform of wave functions which we normalize to be unitary.) Because of the antisymmetry of the wave functions, the sum of the Laplacians is typically of order $N^{5/3}$, as may be seen most easily from the the non-interacting case $V=0$, where the ground state is a Slater determinant of *N* plane waves $f_k(x) = (2\pi )^{-3/2} e^{ik\cdot x}$, the momenta $k \in \mathbb {Z}^3$ being located in a ball of radius proportional to $N^{1/3}$. To make both kinetic and potential energy scale extensively (that is, proportionally to the number of particles *N*) we set

$$\begin{aligned} \hbar := N^{-1/3} \quad \text{ and } \quad \lambda := N^{-1}\;.\end{aligned}$$

This is interpreted as a mean-field limit coupled to a semiclassical limit with effective Planck constant $\hbar = N^{-1/3} \rightarrow 0$ as $N \rightarrow \infty$; this scaling limit has been introduced by [25, 28] to derive the Vlasov equation from many-body quantum mechanics.

We are interested in the ground state energy

$$\begin{aligned} E_N := \inf \text {spec} (H_N) = \inf \left\{ \langle \psi , H_N \psi \rangle : \psi \in L^2_\text{ a } (\mathbb {T}^{3N}) ,\ \Vert \psi \Vert = 1 \right\} \;. \end{aligned}$$

A first approximation for $E_N$ is the Hartree–Fock energy, defined by restricting the variational problem to Slater determinants, that is,

$$\begin{aligned} E_N^\text{ HF } := \inf \Big \{ \langle \psi , H_N \psi \rangle : \psi = \bigwedge _{j=1}^N u_j \text { where } \{ u_j \}_{j=1}^N \text { is an orthonormal family in}\, L^2 (\mathbb {T}^3) \Big \}\;. \end{aligned}$$

As already mentioned, for the non-interacting case $V=0$, the Hartree–Fock and the many-body ground state energy are attained by the Fermi ball

1.2 $$\begin{aligned} \psi _\text{ F }:= \bigwedge _{k \in B_\text{ F }} f_k\;, \end{aligned}$$

with the plane waves $f_k (x) := (2\pi )^{-3/2} e^{ik\cdot x}$, for $x \in \mathbb {T}^3$ and $k \in \mathbb {Z}^3$. Here, the Fermi ball $B_\text{ F }$ is a set of *N* different momenta $p \in \mathbb {Z}^3$ with $\sum _{p}|p|^2$ as small as possible. To simplify our analysis we assume that the Fermi ball is completely filled and thus uniquely defined, that is, that $B_\text{ F }= \{ k \in \mathbb {Z}^3 : |k| \leqq k_\text{ F }\}$. This can be achieved by considering a sequence $k_\text{ F }\rightarrow \infty$ and fixing $N := |B_\text{ F }|$ as a function of $k_\text{ F }$. We find the relation $k_\text{ F }= \kappa N^{1/3}$ between the two parameters, with $\kappa = \kappa _0 + \mathcal {O}(N^{-1/3})$ and $\kappa _0 := (3/4\pi )^{1/3}$.

Under the assumption of a complete Fermi ball and non-negative $\hat{V}$, it was proven in [5, Theorem A.1] that the Hartree–Fock energy $E_N^\text{ HF }$ is still attained by the Fermi ball (1.2), even when $V \not = 0$. It follows that

1.3 $$\begin{aligned} E_N^\text{ HF } = \langle \psi _\text{ F }, H_N \psi _\text{ F }\rangle = \sum _{p \in B_\text{ F }} \hbar ^2 p^2 + \frac{N}{2} \hat{V} (0) - \frac{1}{2N} \sum _{k,k' \in B_F} \hat{V} (k - k') \;. \end{aligned}$$

In this paper we focus on the *correlation energy*, defined as the difference $E_N - E_N^\text{ HF }$, due to many-body interactions among particles. The following theorem, our main result, provides an explicit formula for the dominant order (order $\hbar$) of the correlation energy:

### Theorem 1.1

(Main result: RPA correlation energy) Suppose $V \in L^1 (\mathbb {T}^3)$ with $\hat{V} \geqq 0$ and

$$\begin{aligned} \sum _{k \in \mathbb {Z}^3} \hat{V} (k) |k| < \infty \;. \end{aligned}$$

For $k_\text{ F }> 0$ let $N := | B_\text{ F }| = |\{ k \in \mathbb {Z}^3 : |k| \leqq k_\text{ F }\}|$. Then there exists $\alpha > 0$ such that

1.4 $$\begin{aligned} E_N = E_N^\text{ HF } + E_N^\text{ RPA } + \mathcal {O}(N^{-1/3-\alpha }) \qquad \text {for}\, k_\text{ F }\rightarrow \infty , \end{aligned}$$

where the RPA energy formula is

1.5 $$\begin{aligned} E_N^\text{ RPA } := \hbar \kappa _0 \sum _{k \in \mathbb {Z}^3} |k| \left( \frac{1}{\pi } \int _0^\infty \log \left( 1 + 2\pi \kappa _0 \hat{V} (k) \Big (1-\lambda \arctan \big (\frac{1}{\lambda }\big ) \Big ) \right) {\text{ d }}\lambda - \frac{\pi }{2} \kappa _0 \hat{V} (k) \right) . \end{aligned}$$

### Remarks

(i) Unlike the result of [5], where $\Vert V\Vert _{\ell ^\infty }$ was assumed to be small, here we do not assume smallness of the interaction potential.
(ii) A further generalization is given in Appendix A: there, the upper bound of (1.4) is shown to hold assuming only $\hat{V} \geqq 0$ and $\sum _{k \in \mathbb {Z}^3} |k| \hat{V} (k)^2 < \infty$. Thanks to only the second power of the potential appearing, this almost covers the Coulomb potential. While our paper was under review, a new upper bound for the correlation energy has been established in [14] for square integrable potentials; this includes potentials with Coulomb singularity. In this case, an additional second order contribution to the exchange energy, which is part of the error in our setting, becomes relevant.

In the next section we will introduce the correlation Hamiltonian which describes corrections to Hartree–Fock theory. In Sect. 3 we give a heuristic introduction to the bosonization method by which the correlation Hamiltonian can be approximately diagonalized. The remaining sections are dedicated to the steps of the rigorous implementation of this strategy, culminating in the proof of Theorem 1.1 in Sect. 9.

## Correlation Hamiltonian

As the first step to the proof of Theorem 1.1, we apply a particle–hole transformation to the Hamiltonian, by which we obtain the *correlation Hamiltonian* which describes only the corrections to mean-field (Hartree–Fock) theory. This is an exact computation not involving any approximation.

We use second quantization on the fermionic Fock space $\mathcal {F}= \bigoplus _{n \geqq 0} L^2 (\mathbb {T}^3)^{\otimes _a n}$. On $\mathcal {F}$, we use the well-known creation and annihilation operators satisfying canonical anticommutation relations, namely for all momenta $p,q \in \mathbb {Z}^3$ we have

2.1 $$\begin{aligned} \{ a_p , a_q^* \} = \delta _{p,q}, \quad \{ a_p, a_q \} = \{ a_p^* , a_q^* \} = 0. \end{aligned}$$

As a simple consequence of (2.1), we find the operator norms $\Vert a_p^* \Vert _\text{ op }\leqq 1$ and $\Vert a_p \Vert _\text{ op }\leqq 1$ for all $p \in \mathbb {Z}^3$. We define the vacuum vector $\Omega = ( 1, 0, 0, \dots ) \in \mathcal {F}$ and the number-of-fermions operator $\mathcal {N}= \sum _{p \in \mathbb {Z}^3} a_p^* a_p$. We extend the Hamiltonian (1.1) to the full Fock space $\mathcal {F}$ setting

2.2 $$\begin{aligned} \mathcal {H}_N := \sum _{p \in \mathbb {Z}^3} \hbar ^2 p^2 a_p^* a_p + \frac{1}{2N} \sum _{k,p,q \in \mathbb {Z}^3} \hat{V} (k) a_{p+k}^* a_{q-k}^* a_q a_p \;.\end{aligned}$$

The restriction of $\mathcal {H}_N$ to the *N*-particle sector $L^2_\text{ a } (\mathbb {T}^{3N}) \subset \mathcal {F}$ coincides with (1.1).

To analyse the correlation energy $E_N - E_N^\text{ HF }$, it is convenient to factor out the Fermi ball (1.2) and focus on its excitations. This is achieved through a *particle–hole transformation* $R_\text{ F }: \mathcal {F}\rightarrow \mathcal {F}$ defined by

2.3 $$\begin{aligned} R_\text{ F}^* a_p^* R_\text{ F }:= \left\{ \begin{array}{ll} a_p^* \quad \text {if } p \in B_\text{ F}^c \\ a_p \quad \text {if } p \in B_\text{ F }\end{array} \right. \qquad \text{ and } \qquad R_\text{ F }\Omega := \bigwedge _{p \in B_\text{ F }} f_p = \psi _\text{ F }\;. \end{aligned}$$

One has $R_\text{ F }= R_\text{ F}^* = R_\text{ F}^{-1}$. With (2.3) we find that

$$\begin{aligned} R_\text{ F}^* \mathcal {N}R_\text{ F }= \sum _{p \in B_\text{ F }} a_p a_p^* + \sum _{p \in B_\text{ F}^c} a_p^* a_p = N - \sum _{p\in B_\text{ F }} a_p^* a_p + \sum _{p \in B_\text{ F}^c} a_p^* a_p = N - \mathcal {N}_\text{ h } + \mathcal {N}_\text{ p }, \end{aligned}$$

where we defined the number-of-holes operator $\mathcal {N}_\text{ h } := \sum _{h \in B_\text{ F }} a_h^* a_h$ and the number-of-particles operator $\mathcal {N}_\text{ p } := \sum _{p \in B_\text{ F}^c} a_p^* a_p$. This shows that the *N*-particle sector $L^2_\text{ a } (\mathbb {T}^{3N}) \subset \mathcal {F}$ is the image under $R_\text{ F }$ of the eigenspace of $\mathcal {N}_\text{ h } - \mathcal {N}_\text{ p }$ associated with the eigenvalue 0 (and thus $R_\text{ F }$ defines a unitary map from the eigenspace $\chi (\mathcal {N}_\text{ h } - \mathcal {N}_\text{ p } = 0) \mathcal {F}$ to $L^2_\text{ a } (\mathbb {T}^{3N})$).

We introduce the correlation Hamiltonian $\mathcal {H}_\text{ corr }$ by conjugating $\mathcal {H}_N$ with $R_\text{ F }$ and subtracting the energy of the Fermi ball (which, as already noted in [5, Theorem A.1], in our scaling limit and with $\hat{V} \geqq 0$ equals the Hartree–Fock ground state energy). With (2.3) and the canonical anticommutation relations eqcrefeq:CAR, a lengthy but straightforward computation leads to the *correlation Hamiltonian*

2.4 $$\begin{aligned} \mathcal {H}_\text{ corr } := R_\text{ F}^* \mathcal {H}_N R_\text{ F }- E_N^\text{ HF } = \mathbb {H}_0 + Q_\text{ B } + \mathcal {E}_1 + \mathcal {E}_2 + \mathbb {X}\end{aligned}$$

with the main terms

2.5 $$\begin{aligned} \begin{aligned} \mathbb {H}_0&:= \sum _{p \in \mathbb {Z}^3} e(p) \, a_p^* a_p \; , \qquad \text {with } e(p) := |\hbar ^2 p^2 - \kappa ^2| \; , \\ Q_\text{ B }&:= \frac{1}{N} \sum _{k \in \mathbb {Z}^3} \hat{V} (k) \left( b^* (k) b (k) + \frac{1}{2} \left( b^* (k) b^* (-k) + b(-k) b (k) \right) \right) \end{aligned} \end{aligned}$$

and the error terms

2.6 $$\begin{aligned} \begin{aligned} \mathbb {X}&:= - \frac{1}{2N} \sum _{k \in \mathbb {Z}^3} \hat{V}(k) \bigg ( \sum _{p \in B_\text{ F}^c\cap (B_\text{ F }+k)} a^*_p a_p + \sum _{h \in B_\text{ F }\cap (B_\text{ F}^c-k)} a^*_{h} a_{h}\bigg )\;, \\ \mathcal {E}_1&:= \frac{1}{2N} \sum _{k \in \mathbb {Z}^3} \hat{V} (k) d^* (k) d(k) \;, \\ \mathcal {E}_2&:= \frac{1}{2N} \sum _{k \in \mathbb {Z}^3} \hat{V} (k) \left[ d^* (k) b (-k) + \text {h.c.} \right] \;. \end{aligned} \end{aligned}$$

Here we defined the delocalized particle–hole pair creation and annihilation operators

2.7 $$\begin{aligned} b^* (k) := \sum _{p \in B_\text{ F}^c \cap (B_\text{ F }+ k)} a_p^* a_{p-k}^* , \qquad b (k) := \sum _{p \in B_\text{ F}^c \cap (B_\text{ F }+ k)} a_{p-k} a_{p} \end{aligned}$$

and the non-bosonizable operators

2.8 $$\begin{aligned} d^* (k) := \sum _{p \in B_\text{ F}^c \cap (B_\text{ F}^c + k)} a_p^* a_{p-k} - \sum _{h \in B_\text{ F }\cap (B_\text{ F }-k)} a_h^* a_{h+k} \;, \end{aligned}$$

satisfying $d^* (k) = d (-k)$ for all $k \in \mathbb {Z}^3$.

To prove Theorem 1.1, we improve the bosonization method introduced in [4] for the upper bound and show that

$$\begin{aligned} \inf _{\begin{array}{c} \psi \in \mathcal {F}: \Vert \psi \Vert =1\\ (\mathcal {N}_\text{ p }-\mathcal {N}_\text{ h})\psi = 0 \end{array}} \langle \psi , \mathcal {H}_\text{ corr } \psi \rangle = E^\text{ RPA}_N + \mathcal {O}(N^{-1/3-\alpha })\;.\end{aligned}$$

## Strategy of the Proof: Approximate Bosonization

The key idea is to derive, from the fermionic correlation Hamiltonian (2.4), a quadratic, approximately bosonic^1^, Hamiltonian which can be approximately diagonalized by a Bogoliubov transformation to obtain the ground state energy.

**Fig. 1:**
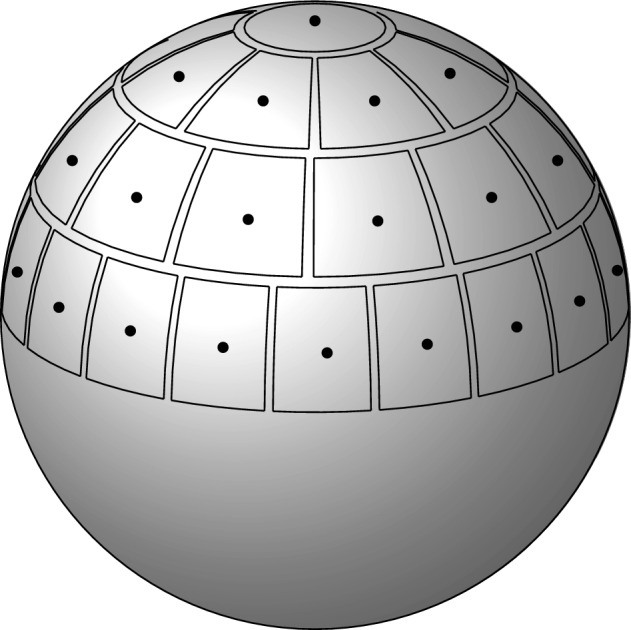
Decomposition of (a shell around) the Fermi surface into patches. The vectors $\hat{\omega }_\alpha$ (marked with dots) are the patch centers. The decomposition of the southern half sphere is obtained through reflection by the origin; see [4] for the details of the construction

The starting point is the observation that the particle–hole pair operators behave approximately as bosonic creation and annihilation operators, that is, they approximately satisfy canonical commutator relations:

$$\begin{aligned} {[}b^*(k),b^*(l)] = 0 = [b(k),b(l)]\;, \ [b(k),b^*(l)] = \text{ const. }\times (\delta _{k,l} + \text{ lower } \text{ order})\;.\end{aligned}$$

Thus $Q_\text{ B }$ can be understood as an approximately bosonic quadratic Hamiltonian. The terms $\mathbb {X}$, $\mathcal {E}_1$, and $\mathcal {E}_2$ do not have a bosonic interpretation and are going to be estimated as smaller errors. It remains to bosonize the kinetic energy $\mathbb {H}_0$. Because this step requires us to linearize the dispersion relation, we need to localize of the pair operators to patches $B_\alpha$, that is, to *M* small regions covering a shell around the Fermi sphere in momentum space (see Fig. 1 for an illustration of the patch decomposition we have in mind; eventually the number of patches *M* will be chosen to tend to infinity as $N\rightarrow \infty$)

3.1 $$\begin{aligned} b^*(k) \simeq \sum _{\alpha =1}^M n_\alpha (k) b^*_\alpha (k)\;, \qquad b^*_\alpha (k) := \frac{1}{n_\alpha (k)} \sum _{\begin{array}{c} p:p \in B_\text{ F}^c \cap B_\alpha \\ p-k\in B_\text{ F }\cap B_\alpha \end{array}} a^*_p a^*_{p-k}\;, \end{aligned}$$

with a normalization constant $n_\alpha (k)$ so that the one-pair states $b^*_\alpha (k)\Omega$ have norm one. There is a catch here: the sum over pairs in (3.1) is only non-empty if the relative momentum *k* is pointing outward from the Fermi ball, so for about half of the possible values of $\alpha$ the operators $b^*_\alpha (k)$ vanish. To be sure that many particle–hole pairs contribute to the sum defining $b^*_\alpha (k)$, we introduce a cutoff by defining the index set

$$\begin{aligned} \mathcal {I}_k^+ := \left\{ \alpha \in \{1,2,\ldots ,M\}: k \cdot \hat{\omega }_\alpha \geqq N^{-\delta } \right\} \end{aligned}$$

(with $\delta > 0$ to be optimized at the end) and combine the retained $b^*_\alpha (k)$-operators into

$$\begin{aligned} c^*_\alpha (k) := \left\{ \begin{array}{ll} b^*_\alpha (k) &{} \text{ for } \alpha \in \mathcal {I}_k^+ \\ b^*_\alpha (-k) &{} \text{ for } \alpha \in \mathcal {I}_{-k}^+ \;. \end{array} \right. \end{aligned}$$

These operators again behave approximately bosonic in the sense that

3.2 $$\begin{aligned} {[}c^*_\alpha (k), c^*_\beta (l)] = 0 = [c_\alpha (k),c_\beta (l)]\;, \quad [c_\alpha (k),c^*_\beta (l)] = \delta _{\alpha ,\beta } \left( \delta _{k,l} + \mathcal {O}\left( \frac{\mathcal {N}}{n_\alpha (k)^2} \right) \right) \;. \end{aligned}$$

This provides important intuition on how to make the approximate bosonization rigorous: because $n_\alpha (k)^2$ counts the number of particle–hole pairs of relative momentum *k* in patch $B_\alpha$, we need the size of the patches to be sufficiently big and we need to bound the number of excitations counted by $\mathcal {N}$ in states close to the ground state.

By virtue of the localization to patches we can linearize the dispersion relation *e*(*p*) locally in every patch, and thus find (the computation here shown for the case $\alpha \in \mathcal {I}_{k}^{+}$)

3.3 $$\begin{aligned} \begin{aligned} {[}\mathbb {H}_0, c^*_\alpha (k)]&= \frac{1}{n_\alpha (k)} \sum _{\begin{array}{c} p:p \in B_\text{ F}^c \cap B_\alpha \\ p-k\in B_\text{ F }\cap B_\alpha \end{array}} \left( e(p) - e(p-k) \right) a^*_p a^*_{p-k}\\&= \frac{1}{n_\alpha (k)} \sum _{\begin{array}{c} p:p \in B_\text{ F}^c \cap B_\alpha \\ p-k\in B_\text{ F }\cap B_\alpha \end{array}} \hbar ^2 \left( 2 p\cdot k - |k|^2 \right) a^*_p a^*_{p-k} \\&\simeq \frac{1}{n_\alpha (k)} \sum _{\begin{array}{c} p:p \in B_\text{ F}^c \cap B_\alpha \\ p-k\in B_\text{ F }\cap B_\alpha \end{array}} 2 \hbar ^2 \, \omega _\alpha \cdot k\, a^*_p a^*_{p-k}\\&\simeq [\mathbb {D}_\text{ B }, c^*_\alpha (k)] \end{aligned}\end{aligned}$$

if we introduce the quadratic approximately bosonic operator

$$\begin{aligned} \mathbb {D}_\text{ B }= 2 \kappa \hbar \sum _{k \in \Gamma ^{\text{ nor }}} \sum _{\alpha =1}^M |k \cdot \hat{\omega }_\alpha | \, c_\alpha ^* (k) c_\alpha (k)\;.\end{aligned}$$

While the substitution of $\mathbb {H}_0$ by $\mathbb {D}_\text{ B }$ has here been motivated only in commutators with almost bosonic operators, a key step of our analysis is to justify this step also on general states close to the ground state. This step is explained in (3.8) to (3.11).

Our further goal is to approximately (to order $\hbar$, the dominant contribution of the correlation energy) diagonalize the bosonic quadratic Hamiltonian $\mathbb {D}_\text{ B }+ Q_\text{ B }$ by an approximately bosonic Bogoliubov transformation *T*, allowing us to read off the correlation energy. Given a state $\psi \in \mathcal {F}$ such that $(\mathcal {N}_{\text {p}} - \mathcal {N}_{\text {h}}) \psi = 0$ (think of the ground state of $\mathcal {H}_\text {corr}$), and setting $\xi := T^{*} \psi$, we write

3.4 $$\begin{aligned} \begin{aligned} \langle \psi , \mathcal {H}_{\text {corr}} \psi \rangle&= \langle T \xi , \mathcal {H}_{\text {corr}} T \xi \rangle \\&= \langle T\xi , ( \mathbb {D}_{\text {B}} + Q_{\text {B}} ) T \xi \rangle + \langle T\xi , ( \mathbb {H}_{0} - \mathbb {D}_{\text {B}} ) T\xi \rangle \\&\quad + \langle T\xi , ( \mathbb {X} + \mathcal {E}_{1} + \mathcal {E}_{2} ) T\xi \rangle \;. \end{aligned} \end{aligned}$$

Through a suitable choice of the Bogoliubov kernel *K*(*k*) (a matrix indexed by the patch labels), the approximate Bogoliubov transformation

3.5 $$\begin{aligned} T = \exp \Bigg ( \frac{1}{2} \sum _{k \in \Gamma ^{\text{ nor }}} \sum _{\alpha ,\beta \in \mathcal {I}^{+}_k \cup \mathcal {I}^{+}_{-k}} K (k)_{\alpha ,\beta } \, c_\alpha ^* (k) c_\beta ^* (k) - \text {h.c.} \Bigg ) \end{aligned}$$

diagonalizes approximately the quadratic Hamiltonian $\mathbb {D}_{\text {B}} + Q_{\text {B}}$. On states with few particles (ie. with few excitations of the Fermi sea), we find as suggested by exact bosonic Bogoliubov theory that

3.6 $$\begin{aligned} \langle T\xi , ( \mathbb {D}_{\text {B}} + Q_{\text {B}} ) T \xi \rangle \simeq E^{\text {RPA}}_{N} + \langle \xi , \mathcal {H}^{\text {exc}}_{\text {B}} \xi \rangle \;, \end{aligned}$$

with the intended $E^{\text {RPA}}_{N}$ as in (1.5), and for the description of the possible bosonic excitation one obtains an effective Hamiltonian of the form

3.7 $$\begin{aligned} \mathcal {H}^{\text {exc}}_{\text {B}} = \sum _{k \in \Gamma ^{\text{ nor }}} \sum _{\alpha ,\beta \in \mathcal {I}^{+}_k \cup \mathcal {I}^{+}_{-k}} 2 \hbar \kappa |k|\mathfrak {K}(k)_{\alpha ,\beta } c^*_\alpha (k) c_\beta (k) \geqq 0 \;. \end{aligned}$$

To make these heuristics rigorous, apart from controlling the bosonic approximation (arising from the neglect of the error term in (3.2)) in the bosonic Bogoliubov diagonalization, we need to estimate the second and the third terms in (3.4). There are two obstacles. One is to give a meaning to the heuristics $\mathbb {H}_0 \simeq \mathbb {D}_\text{ B }$, which, a priori, holds only as in (3.3), at the level of commutators with the approximately bosonic operators. The other is to control the non-bosonizable term $\mathcal {E}_1$ and the term $\mathcal {E}_2$ which couples almost bosonic *c*-operators to non-bosonizable *d*-operators. (The exchange term $\mathbb {X}$ instead can be controlled by more elementary estimates.)

Both problems were solved in [5] under the assumption that the interaction potential *V* is small and compactly supported in Fourier space. In the present work we overcome these limitations and prove the validity of the random-phase approximation for a much larger class of interaction potentials. The main achievements of the present paper, compared to [4, 5], are the following:

- The combination $\mathbb {H}_{0} - \mathbb {D}_{\text {B}}$ is approximately invariant under conjugation with the approximately bosonic Bogoliubov transformation because its action can be expanded in commutators:
  3.8 $$\begin{aligned} \langle T\xi , ( \mathbb {H}_{0} - \mathbb {D}_{\text {B}} ) T\xi \rangle \simeq \langle \xi , ( \mathbb {H}_{0} - \mathbb {D}_{\text {B}} ) \xi \rangle \;. \end{aligned}$$
  In the proof of the upper bound for the correlation energy, the vector $\xi$ coincides with the vacuum, and the right-hand side is zero. For the lower bound this is not true, and we are left with controlling the negative term $-\mathbb {D}_{\text {B}}$. In [5], this was achieved by exploiting the positivity of $\mathcal {H}^{\text {exc}}_{\text {B}}$ in (3.6). More precisely, we proved that
  $$\begin{aligned} \langle \xi , \mathcal {H}^{\text {exc}}_{\text {B}} \xi \rangle \geqq \langle \xi , \mathbb {D}_\text {B} \xi \rangle - C \Vert {\hat{V}} \Vert _{1} \langle \xi , \mathbb {H}_{0} \xi \rangle , \end{aligned}$$
  which, for small potential, is enough to control the right-hand side of (3.8). In the present paper, we need a more refined analysis. In order to compare $\mathcal {H}_B^\text {exc}$ with $\mathbb {D}_\text {B}$, we need to diagonalize the matrix $\mathfrak {K}(k)_{\alpha ,\beta }$ appearing on the right-hand side of (3.7) (because $\mathbb {D}_\text {B}$ is already expressed through a diagonal matrix). This can be achieved through a second approximately bosonic Bogoliubov transformation having the form
  3.9 $$\begin{aligned} Z = \exp \Bigg ( \sum _{k \in \Gamma ^{\text{ nor }}} \sum _{\alpha ,\beta \in \mathcal {I}^{+}_k \cup \mathcal {I}^{+}_{-k}} L (k)_{\alpha ,\beta } \, c_\alpha ^* (k) c_\beta (k) \Bigg ) \end{aligned}$$
  for an antisymmetric matrix $L (k)_{\alpha ,\beta }$. If $c^*$ and *c* were bosonic operators, we could write $Z = \exp \big ({\sum _{k \in \Gamma ^{\text{ nor }}} {\text{ d }}\Gamma (L (k))}\big ) = \prod _{k \in \Gamma ^{\text{ nor }}} \Gamma (e^{L (k)})$ (where ${\text{ d }}\Gamma$ and $\Gamma$ are the operators of bosonic second quantization) and its action on (3.7) would be simply
  $$\begin{aligned} Z^* \mathcal {H}_B^\text {exc} Z = \sum _{k \in \Gamma ^{\text{ nor }}} {\text{ d }}\Gamma (e^{-L (k)} \mathfrak {K} (k) e^{L (k)}) \;, \end{aligned}$$
  that is, conjugation of $\mathfrak {K}(k)$ by the one-boson unitary $e^{L(k)}$. This would allow us to diagonalize the matrix $\mathfrak {K} (k)$ by an appropriate choice of *L*(*k*). Even though *c* and $c^*$ are not exactly bosonic operators, this remains approximately true on states with few excitations. After this diagonalization, it is simple to compare with $\mathbb {D}_B$ and conclude that (up to subleading error terms)
  3.10 $$\begin{aligned} Z^* \mathcal {H}^{\text {exc}}_{\text {B}} Z \gtrsim \mathbb {D}_{\text {B}}\;. \end{aligned}$$
  Since, similarly to (3.8), also *Z* leaves the difference $\mathbb {H}_0 - \mathbb {D}_\text {B}$ almost invariant (the fact that *Z* can be expressed in terms of almost bosonic operators by (3.3) implies $[Z, \mathbb {H}_0 - \mathbb {D}_\text {B}] \simeq 0$), we obtain, with (3.10), the desired lower bound
  3.11 $$\begin{aligned} \begin{aligned}&\langle T Z \xi , (\mathbb {D}_\text {B} + Q_\text {B}) T Z \xi \rangle + \langle TZ \xi , (\mathbb {H}_0 - \mathbb {D}_\text {B}) TZ \xi \rangle \\&\quad \simeq E_N^\text {RPA} + \langle Z \xi , \mathcal {H}_\text {B}^\text {exc} Z \xi \rangle + \langle \xi , (\mathbb {H}_0 - \mathbb {D}_B) \xi \rangle \gtrsim E_N^\text {RPA} \, . \end{aligned} \end{aligned}$$
- In [5], we controlled the non-bosonizable error terms as, informally stated, $T^*(\mathcal {E}_1 + \mathcal {E}_2)T \gtrsim - C \Vert \hat{V}\Vert _{\ell ^1} \mathbb {H}_0$, explaining the necessity of the interaction potential being small to control this term by a positive $\mathbb {H}_0$. In the present paper instead we control $\mathcal {E}_{1}$ more precisely. In particular, we prove that on states $\xi$ close to the ground state of the correlation Hamiltonian, the following improved bound holds true (see Lemma 4.8):
  3.12 $$\begin{aligned} \langle T\xi , \mathcal {E}_{1} T\xi \rangle \ll C\hbar \;. \end{aligned}$$
  This means that the contribution of the non-bosonizable term $\mathcal {E}_{1}$ to the energy is subleading with respect to $E^{\text {RPA}}_{N}$, which is of order $\hbar$. Concerning $\mathcal {E}_{2}$, by the Cauchy–Schwarz inequality we get (see Corollary 4.9)
  $$\begin{aligned} \pm \mathcal {E}_{2} \leqq C N^{\alpha } \mathcal {E}_{1} + C\Vert {\hat{V}} \Vert _{1}N^{-\alpha }\mathbb {H}_{0} \;.\end{aligned}$$
  The first term in the bound is controlled by the improved bound (3.12), while the second term is controlled by positivity of $\langle T\xi , \mathbb {H}_{0} T\xi \rangle$ in (3.4), for *N* large enough without any smallness assumption on *V*.
- Furthermore, to implement this strategy, we improve the a-priori bounds on the number and the energy of excitations: our Lemma 4.1 and Corollary 4.2 generalize estimates of [5] to interaction potentials with $\hat{V} \geqq 0$ and $|\cdot | \hat{V} \in \ell ^1 (\mathbb {Z}^3)$. Moreover, Lemma 4.3 now holds uniformly in *k*.

The rigorous implementation is the subject of all remaining sections.

## A-Priori Estimates on Excitations of the Fermi Ball

The following lemma shows that vectors with total energy close to the ground state energy contain also only a small amount of kinetic energy:

### Lemma 4.1

(A-priori bound on kinetic energy) Assume $\sum _{k \in \mathbb {Z}^3} |\hat{V} (k)||k| < \infty$ and $\hat{V}\geqq 0$. Then there exists a $C >0$ such that we have

$$\begin{aligned} \mathcal {H}_\text{ corr } = R_\text{ F}^* H_N R_\text{ F }- E_N^\text{ HF } \geqq \mathbb {H}_0 - C \hbar \;. \end{aligned}$$

Hence, for every $\psi \in L^2_\text{ a } (\mathbb {T}^{3N})$ with $\Vert \psi \Vert = 1$ and $\langle \psi , H_N \psi \rangle \leqq E_N^\text{ HF } + C \hbar$ the excitation vector $\xi = R_\text{ F}^* \psi \in \mathcal {F}$ satisfies

$$\begin{aligned} \langle \xi , \mathbb {H}_0 \xi \rangle \leqq C \hbar \;. \end{aligned}$$

### Remark

In the present paper we will apply Lemma 4.1 to the ground state $\psi _\text{ gs }$, which by the variational principle even satisfies $\langle \psi _\text{ gs } , H_N \psi _\text{ gs } \rangle \leqq E_N^\text{ HF }$.

### Proof of Lemma 4.1

From $\hat{V} \geqq 0$ we get

$$\begin{aligned} \begin{aligned} 0&\leqq \int _{\mathbb {T}^3 \times \mathbb {T}^3} V (x-y) \Bigg ( \sum _{j=1}^N \delta (x_j - x) - N \Bigg ) \Bigg ( \sum _{i=1}^N \delta (x_i - y) - N \Bigg ) {\text{ d }}x {\text{ d }}y \\&= 2 \sum _{i<j}^N V(x_i - x_j) + N V (0) - N^2 \hat{V} (0) \;. \end{aligned} \end{aligned}$$

Thus

$$\begin{aligned} H_N \geqq \sum _{j=1}^N -\hbar ^2 \Delta _{x_j} + \frac{N}{2} \hat{V} (0) - \frac{V(0)}{2}\;. \end{aligned}$$

Switching to Fock space $\mathcal {F}$ and conjugating with $R_\text{ F }$, we conclude that

4.1 $$\begin{aligned} R_\text{ F}^* \mathcal {H}_N R_\text{ F }\geqq \sum _{p \in \mathbb {Z}^3} \hbar ^2 p^2 R_\text{ F}^* a_p^* a_p R_\text{ F }+ \frac{N}{2} \hat{V} (0) - \frac{V(0)}{2} = \mathbb {H}_0 + \sum _{p \in B_\text{ F }} \hbar ^2 p^2 + \frac{N}{2} \hat{V} (0) - \frac{V(0)}{2} \;. \end{aligned}$$

We compare the right-hand side of (4.1) with the Hartree–Fock energy (1.3). We have

$$\begin{aligned} \frac{1}{2N} \sum _{k,k' \in B_\text{ F }} \hat{V} (k-k') = \frac{V(0)}{2} - \frac{1}{2N} \sum _{k \in B_\text{ F }} \sum _{k' \in B_\text{ F}^c} \hat{V} (k-k') \;. \end{aligned}$$

Setting $q = k-k'$ and noting that $| B_\text{ F }\cap (B_\text{ F}^c + q)| \leqq C |q| N^{2/3}$, we estimate

$$\begin{aligned} \begin{aligned} \frac{1}{2N} \sum _{k \in B_\text{ F }} \sum _{k' \in B_\text{ F}^c} \hat{V} (k-k')&= \frac{1}{2N} \sum _{k \in B_\text{ F }} \sum _{q \in B_\text{ F}^c +k} \hat{V} (q) \\ {}&= \frac{1}{2N} \sum _{q \in \mathbb {Z}^3} \hat{V} (q) \sum _{k \in B_\text{ F }\cap (B_\text{ F}^c +q)} 1 \leqq C \hbar \sum _{q \in \mathbb {Z}^3} \hat{V} (q) |q| \;. \end{aligned} \end{aligned}$$

By assumption on *V*, this implies that

$$\begin{aligned} \frac{1}{2N} \sum _{k,k' \in B_\text{ F }} \hat{V} (k-k') \geqq \frac{V(0)}{2} - C \hbar \;. \end{aligned}$$

With (1.3) and (4.1) we conclude that $R_\text{ F}^* \mathcal {H}_N R_\text{ F }\geqq E_N^\text{ HF } + \mathbb {H}_0 - C \hbar$. $\square$

The a-priori bound from Lemma 4.1 for the kinetic energy $\mathbb {H}_0$ has several consequences. First of all, it gives control on the number of excitations of the Slater determinant. Here, it is useful to introduce gapped number-of-fermions operators which are easier to control than $\mathcal {N}$. For $\varepsilon > 0$, we define the gapped number operator

4.2 $$\begin{aligned} \mathcal {N}_\varepsilon := \sum _{p \in \mathbb {Z}^3 :\, ||p| - k_\text{ F }| > N^{-\varepsilon }} a_p^* a_p \end{aligned}$$

measuring the number of excitations with momenta further than a distance $N^{-\varepsilon }$ from the Fermi sphere. (The Definition (4.2) differs slightly from the definition used in [5] but that is merely a matter of convenience.)

### Corollary 4.2

(A-priori bounds on particle number) There exists a constant $C > 0$ such that, on $\chi (\mathcal {N}_\text{ p } - \mathcal {N}_\text{ h } = 0) \mathcal {F}$, we have

4.3 $$\begin{aligned} \begin{aligned} \mathcal {N}\leqq C N^{2/3} \mathbb {H}_0 \quad \text {and} \quad \mathcal {N}_\varepsilon \leqq C N^{1/3+\varepsilon } \mathbb {H}_0 \quad \text {for every}\, \varepsilon > 0. \end{aligned} \end{aligned}$$

Assume furthermore that $\sum _{k \in \mathbb {Z}^3} |\hat{V} (k)||k| < \infty$ and $\hat{V}\geqq 0$. Then, for $\psi \in L^2_\text{ a } (\mathbb {T}^{3N})$ with $\Vert \psi \Vert = 1$ and $\langle \psi , H_N \psi \rangle \leqq E_N^\text{ HF } + C \hbar$, the excitation vector $\xi = R_\text{ F}^* \psi \in \mathcal {F}$ satisfies

4.4 $$\begin{aligned} \langle \xi , \mathcal {N}\xi \rangle \leqq C N^{1/3} \quad \text {and} \quad \langle \xi , \mathcal {N}_\varepsilon \xi \rangle \leqq C N^\varepsilon \quad \text {for every}\, \varepsilon > 0.\end{aligned}$$

### Proof

To prove (4.3) for $\mathcal {N}_\varepsilon$, observe that $||p| - k_\text{ F }| > N^{-\varepsilon }$ implies $|\hbar |p| - \kappa | > \hbar N^{-\varepsilon }$ and thus

$$\begin{aligned} |\hbar ^2 p^2 - \kappa ^2 | \geqq \kappa \hbar N^{-\varepsilon } \;.\end{aligned}$$

Thus

$$\begin{aligned} \mathbb {H}_0 \geqq \sum _{p \in \mathbb {Z}^3 :||p| - k_\text{ F }| > N^{-\varepsilon }} |\hbar ^2 p^2 - \kappa ^2 | a_p^* a_p \geqq \kappa \hbar N^{-\varepsilon } \mathcal {N}_\varepsilon \;.\end{aligned}$$

The bound for $\mathcal {N}$ is proven in [5, Lemma 2.4]; (4.4) follows using Lemma 4.1. $\square$

Furthermore, the estimate for $\mathbb {H}_0$ from Lemma 4.1 allows us to bound the particle–hole pair operators *b*(*k*) and $b^* (k)$ introduced in (2.7).

### Lemma 4.3

(Kinetic bound on particle–hole pairs) There exists a constant $C > 0$ such that, for all $k \in \mathbb {Z}^3$,

4.5 $$\begin{aligned} \sum _{p \in B_\text{ F}^c \cap (B_\text{ F }+ k)} \Vert a_p a_{p-k} \psi \Vert \leqq C N^{1/2} \Vert \mathbb {H}_0^{1/2} \psi \Vert \; \end{aligned}$$

and moreover

4.6 $$\begin{aligned} \sum _{\begin{array}{c} p \in B_\text{ F}^c \cap (B_\text{ F }+ k) : \\ e(p ) + e(p-k) \leqq C N^{-1/3-\delta } \end{array}} \Vert a_p a_{p-k} \psi \Vert \leqq C N^{1/2-\delta /2} \Vert \mathbb {H}_0^{1/2} \psi \Vert \;.\end{aligned}$$

The bounds (4.5) and (4.6) have been established in [5, Appendix B] (and previously in [20, Lemma 4.7]) for fixed *k* (which was sufficient since there only *k* in the compact support of $\hat{V}$ was relevant). Here, we improve the proof given in [5] to obtain uniformity in *k*. We use the following number theoretic result:

### Proposition 4.4

(Lattice points in convex bodies, [22]) Let $K\subset \mathbb {R}^{2}$ be a smooth convex body and let *RK* be its dilation by a factor $R>0$, $R K := \{ x\in \mathbb {R}^{2} \mid x / R \in K \}$. Consider the number of points of $\mathbb {Z}^{2}$ belonging to *RK*,

4.7 $$\begin{aligned} \mathfrak {N}_{K}(R) := \big | \{ n \in \mathbb {Z}^{2} \mid \frac{n}{R} \in K \} \big |\;. \end{aligned}$$

Let

4.8 $$\begin{aligned} \mathcal {E}_{K}(R) := \mathfrak {N}_{K}(R) - R^{2} |K|\;. \end{aligned}$$

Then, for any $\gamma > 131/208$, there exists $C_{K,\gamma }>0$ independent of *R* such that

4.9 $$\begin{aligned} | \mathcal {E}_{K}(R) | \leqq C_{K,\gamma } R^{\gamma }\;. \end{aligned}$$

### Remark

The constant $C_{K,\gamma }$ in the estimate (4.9) depends on the curvature of the boundary of *K*. In particular, $C_{K,\gamma }$ is finite as long as the curvature is strictly positive. For us it is sufficient that (4.9) holds for some $\gamma < 1$. A simple proof for $2/3< \gamma < 1$ is given in [21, Theorem 7.7.16] (the condition $0 \in K$ given there can always be achieved by a translation).

### Proof of Lemma 4.3

We first prove (4.5). Proceeding as in [20, Lemma 4.7] by the Cauchy–Schwarz inequality we get

$$\begin{aligned} \sum _{p \in B_\text{ F}^c\cap (B_\text{ F }+k)} \Vert a_p a_{p-k} \psi \Vert&\leqq \Bigg (\sum _{p \in B_\text{ F}^c\cap (B_\text{ F }+k)} \frac{1}{e(p)+e(p-k)} \Bigg )^{1/2} \\&\quad \times \Bigg (\sum _{p \in B_\text{ F}^c\cap (B_\text{ F }+k)}\left( e(p)+e(p-k) \right) \Vert a_p a_{p-k} \psi \Vert ^2 \Bigg )^{1/2}\;. \end{aligned}$$

The second factor is bounded by the kinetic energy as claimed,

$$\begin{aligned}&\sum _{p \in B_\text{ F}^c\cap (B_\text{ F }+k)}\left( e(p)+e(p-k) \right) \Vert a_p a_{p-k} \psi \Vert ^2 \\&\quad \leqq \sum _{p \in B_\text{ F}^c\cap (B_\text{ F }+k)} e(p) \Vert a_p \psi \Vert ^2 + \sum _{p \in B_\text{ F}^c\cap (B_\text{ F }+k)} e(p-k) \Vert a_{p-k} \psi \Vert ^2 \leqq \langle \psi , \mathbb {H}_0 \psi \rangle \;. \end{aligned}$$

Therefore it is enough to show that

4.10 $$\begin{aligned} \sum _{p \in B_\text{ F}^c \cap (B_\text{ F }+k)} \frac{1}{p^2 - (p-k)^2} \leqq C N^{1/3} \;. \end{aligned}$$

If $|k| > C_0 N^{1/3}$ (for a $C_0 > 0$ large enough), we have $p^2 - (p-k)^2 > C_1 N^{2/3}$ for all $p \in B_\text{ F}^c \cap (B_\text{ F }+ k)$ (with a different constant $C_1 > 0$) and (4.10) is clear. Thus we can assume that from now on

$$\begin{aligned} |k|\leqq C_0 N^{1/3} \;. \end{aligned}$$

We need to further distinguish the cases $p^2 - (p-k)^2 \geqq 4 N^{1/3}$ and $p^2 - (p-k)^2 < 4 N^{1/3}$.

*The case* $p^2 - (p-k)^2 \geqq 4 N^{1/3}$. We apply the argument used in [16, Eq. (5.13)]. If $\eta \in (0, \frac{3}{2C_0})$ then for $q \in B_\eta (p)$ we have

$$\begin{aligned} \begin{aligned} |q^2 - (q-k)^2 |&\geqq \big ||p^2 - (p-k)^2 |- |2(p-q) \cdot k|\big |\geqq 4 N^{1/3} - 2 \eta C_0 N^{1/3}\geqq N^{1/3} \;. \end{aligned} \end{aligned}$$

With

$$\begin{aligned} \nabla _q \frac{1}{q^2 - (q-k)^2} = \frac{2k}{q^2 - (q-k)^2} \frac{1}{q^2 - (q-k)^2} \end{aligned}$$

we conclude that

$$\begin{aligned} \Big | \frac{1}{p^2 - (p-k)^2} - \frac{1}{\widetilde{p}^2 - (\widetilde{p}-k)^2} \Big | \leqq \eta 2 C_0 \sup _{q \in B_\eta (p)} \frac{1}{q^2 - (q-k)^2} \end{aligned}$$

for all $\widetilde{p} \in B_\eta (p)$. Hence, if $\eta > 0$ is small enough, we get

$$\begin{aligned} \sup _{q \in B_\eta (p)} \frac{1}{q^2 - (q-k)^2} \leqq \frac{2}{p^2 - (p-k)^2} \end{aligned}$$

and

$$\begin{aligned} \frac{1}{p^2 - (p-k)^2} \leqq 2 \inf _{q \in B_\eta (p)} \frac{1}{q^2 - (q-k)^2} \;. \end{aligned}$$

Possibly choosing $\eta >0$ still smaller, the balls $B_\eta (p)$ are disjoint for different *p*, and we obtain

$$\begin{aligned} \begin{aligned} \sum _{p \in B_\text{ F}^c \cap (B_\text{ F }+k)} \frac{\chi (p^2 - (p-k)^2 \geqq 4N^{1/3})}{p^2 - (p-k)^2}&\leqq C \int _{p \in B_\text{ F}^c \cap (B_\text{ F }+k)} \frac{1}{p^2 - (p-k)^2} {\text{ d }}p \\ {}&\leqq C N^{1/3} \int _{|p| > 1, |p-k'| < 1} \frac{1}{p^2 - (p-k')^2} {\text{ d }}p \end{aligned} \end{aligned}$$

where we defined $k' := k/ k_\text{ F }$. With

$$\begin{aligned} p^2 - (p-k)^2 = (p^2 - 1) + (1- (p-k)^2) \geqq 2 \big ( p^2 - 1 \big )^{1/2} \big (1- (p-k)^2 \big )^{1/2} \end{aligned}$$

we conclude that

$$\begin{aligned} \begin{aligned}&\sum _{p \in B_\text{ F}^c \cap (B_\text{ F }+k)} \frac{\chi (p^2 - (p-k)^2 \geqq 4N^{1/3})}{p^2 - (p-k)^2} \\&\quad \leqq C N^{1/3} \int _{\begin{array}{c} |p|> 1,\\ |p-k'|< 1 \end{array}} \frac{1}{(p^2-1)^{1/2} (1 - (p-k')^2)^{1/2}} {\text{ d }}p\\&\quad \leqq C N^{1/3} \end{aligned} \end{aligned}$$

uniformly in *k*, as shown in [16, Lemma 3.4].

*The case* $p^2 - (p-k)^2 < 4 N^{1/3}$. We observe that $p \in B_\text{ F}^c$ and $p-k \in B_\text{ F }$ together imply the lower bound (recall that all momenta are elements of $\mathbb {Z}^3$)

$$\begin{aligned} 1 \leqq p^2 - (p-k)^2 = 2p \cdot k - k^2 =: m \in \mathbb {N}. \end{aligned}$$

Since, moreover, $p^2 > k_\text{ F}^2$ and $(p-k)^2 = p^2 - m \leqq k_\text{ F}^2$, we find that

$$\begin{aligned} k_\text{ F}^2 < p^2 \leqq k_\text{ F}^2 + m\;.\end{aligned}$$

We obtain

4.11 $$\begin{aligned} \sum _{p \in B_\text{ F}^c \cap (B_\text{ F }+k)} \frac{\chi (p^2 - (p-k)^2 \leqq 4N^{1/3})}{p^2 - (p-k)^2} \leqq \sum _{m=1}^{4N^{1/3}} \frac{1}{m} |B_m (k)| \end{aligned}$$

with

$$\begin{aligned} B_m (k) := \Big \{ p \in \mathbb {Z}^3 : k_\text{ F}^2 < |p|^2 \leqq k_\text{ F}^2 +m \text { and } 2 p\cdot k - |k|^2 = m \Big \} \;. \end{aligned}$$

Without loss of generality $|k_1| \geqq |k_2|$ and $|k_1| \geqq |k_3|$ (in particular, since $k \not = 0$, we have $k_1 \not = 0$). Then, for $p = (p_1 , p_2, p_3) \in B_m (k)$, the condition $2p\cdot k - |k|^2 =m$ is solved by

4.12 $$\begin{aligned} p_1 = \frac{m+k^2}{2k_1} - p_2 \frac{k_2}{k_1} - p_3 \frac{k_3}{k_1} \;. \end{aligned}$$

Thus $|B_m (k)|$ is bounded by the number of points $(p_2, p_3) \in \mathbb {Z}^2$ with

4.13 $$\begin{aligned} k_\text{ F}^2 \leqq \left( \frac{m+k^2}{2k_1} - p_2 \frac{k_2}{k_1} - p_3 \frac{k_3}{k_1} \right) ^2 + p_2^2 + p_3^2 \leqq k_\text{ F}^2 + m \;.\end{aligned}$$

(This is only an upper bound because $(p_2, p_3) \in \mathbb {Z}^2$ for which the right-hand side of (4.12) is not integer do not contribute to $B_m (k)$). On the $(p_2, p_3)$-plane, we define new variables $(q_2, q_3)$ by

4.14 $$\begin{aligned} \begin{aligned} p_2&:= \frac{k_2}{\sqrt{k_2^2+k_3^2}} q_2 - \frac{k_3}{\sqrt{k_2^2+ k_3^2}} q_3 + \frac{k^2 +m}{2|k|} \frac{\sqrt{k_2^2+ k_3^2}}{|k|} \;, \\ p_3&:= \frac{k_3}{\sqrt{k_2^2+ k_3^2}} q_2 + \frac{k_2}{\sqrt{k_2^2+k_3^2}} q_3 \;.\end{aligned} \end{aligned}$$

In terms of these new variables, we can rewrite (4.13) as

4.15 $$\begin{aligned} k_\text{ F}^2 - \left( \frac{k^2+m}{2|k|} \right) ^2 \leqq \frac{k^2}{k_1^2} q_2^2 + q_3^2 \leqq k_\text{ F}^2 +m - \left( \frac{k^2+m}{2|k|} \right) ^2 \;.\end{aligned}$$

We can therefore apply Proposition 4.4 to estimate the number of points $(p_2, p_3) \in \mathbb {Z}^2$ contained between the two ellipses described by (4.15). (From the assumptions $|k_1| \geqq |k_2|$ and $|k_1| \geqq |k_3|$ we have $1 \leqq |k| / |k_1| \leqq 3$, which implies that the error term in (4.9) is uniform in *k*.) We conclude that

$$\begin{aligned} |B_m (k)| \leqq \pi \frac{k_1}{|k|} m + C k_\text{ F}^\gamma \leqq C (m+N^{\gamma /3}) \qquad \text{ for } \text{ a } \gamma > \frac{131}{208}. \end{aligned}$$

Inserting this bound in (4.11) and choosing $\gamma < 1$ we arrive at

$$\begin{aligned} \sum _{p \in B_\text{ F}^c \cap (B_\text{ F }+k)} \frac{\chi (p^2 - (p-k)^2 \leqq 4N^{1/3})}{p^2 - (p-k)^2} \leqq C \sum _{m=1}^{4N^{1/3}} \frac{1}{m} (m+N^{\gamma /3}) \leqq C N^{1/3} \;. \end{aligned}$$

To show (4.6), we proceed analogously. The only difference is that now the sum in (4.11) can be restricted to $m \leqq C N^{1/3 - \delta }$ (here, the case $p^2 - (p-k)^2 \geqq 4N^{1/3}$ is not relevant). $\square$

From Lemma 4.3, we immediately obtain a bound on the operators *b*(*k*) and $b^* (k)$. For details, see [5, Lemma 2.3].

### Corollary 4.5

(Kinetic bound on pair operators) There exists a $C > 0$ such that for all $k \in \mathbb {Z}^3$ we have

$$\begin{aligned} b^* (k) b (k) \leqq C N \mathbb {H}_0\;, \qquad b (k) b^* (k) \leqq C N (\mathbb {H}_0 + \hbar ) \;. \end{aligned}$$

Using the last corollary, we obtain an a-priori bound for the bosonizable interaction $Q_\text{ B }$.

### Corollary 4.6

(Bosonizable interaction) Assume $\Vert \hat{V} \Vert _1 < \infty$. Then there exists $C > 0$ such that

$$\begin{aligned} -C (\mathbb {H}_0 + \hbar ) \leqq Q_\text{ B } \leqq C (\mathbb {H}_0 + \hbar ) \;.\end{aligned}$$

### Proof

We observe that, for any $k \in \mathbb {Z}^3$, by Corollary 4.5,

$$\begin{aligned} \begin{aligned} 0&\leqq (b^* (k) \pm b (-k)) (b (k) \pm b (-k)) \\&= b^* (k) b (k) + b (-k) b^* (-k) \pm \left[ b^* (k) b^* (-k) + b(-k) b(k) \right] \\ {}&\leqq C N (\mathbb {H}_0 + \hbar ) \pm \left[ b^* (k) b^* (-k) + b(-k) b(k) \right] \;.\end{aligned} \end{aligned}$$

Hence

$$\begin{aligned} -C N (\mathbb {H}_0 + \hbar ) \leqq b^* (k) b^* (-k) + b(-k) b(k) \leqq C N (\mathbb {H}_0 + \hbar ) \, . \end{aligned}$$

After summing over *k*, this implies the desired estimate for $Q_\text{ B }$. $\square$

Finally, the a-priori bound for $\mathbb {H}_0$ (and the resulting estimates on $\mathcal {N}$ and $\mathcal {N}_\varepsilon$ from Corollary 4.2) imply that the error terms in (2.6) are negligible. First of all, the exchange operator $\mathbb {X}$ can be bounded with the following lemma, taken from [5, Lemma 2.5]:

### Lemma 4.7

(Exchange term) Assume $\Vert \hat{V} \Vert _1 < C$. Then there exists a $C >0$ such that for all $\xi \in \chi (\mathcal {N}^p - \mathcal {N}^h = 0) \mathcal {F}$ we have

$$\begin{aligned} |\langle \xi , \mathbb {X}\xi \rangle | \leqq C N^{-1/3} \langle \xi , \mathbb {H}_0 \xi \rangle \;.\end{aligned}$$

The next lemma provides control on the error term $\mathcal {E}_1$ in (2.6). It is one of the key achievements of the present paper.

### Lemma 4.8

(Non-bosonizable interaction) Assume $\Vert \hat{V} \Vert _1 < \infty$. Fix $0< \varepsilon < 1/3$ and $131/208< \gamma < 1$. Then there exists $C > 0$ such that for all $\xi \in \chi (\mathcal {N}_\text{ h } - \mathcal {N}_\text{ p } = 0) \mathcal {F}$ we have

4.16 $$\begin{aligned} \begin{aligned} \langle \xi , \mathcal {E}_1 \xi \rangle \leqq \;&C N^{-1} \Vert (\mathcal {N}+1)^{3/2} \xi \Vert \Vert \mathcal {N}_{1/3-\varepsilon }^{1/2} \xi \Vert +C N^{\varepsilon -1} (N^\varepsilon + N^{\gamma /3}) \Vert \mathcal {N}^{1/2} \xi \Vert ^2 \;. \end{aligned} \end{aligned}$$

### Remark

With a localization argument, we will be able to restrict our attention to states for which $\mathcal {N}\leqq C N^{1/3}$ and $\mathcal {N}_\delta \leqq C N^\delta$ (for the expectation value as stated in Corollary 4.2, but also for higher moments). Applying (4.16) for such states, choosing $\gamma < 1$ and $\varepsilon > 0$ small enough, we conclude that $\mathcal {E}_1 \ll N^{-1/3}$ and therefore that $\mathcal {E}_1$ does not contribute to the correlation energy, to leading order.

### Proof of Lemma 4.8

Recall the Definition (2.8) of the operators $d^* (k)$ and *d*(*k*). Since $d(0) = d^* (0) = 0$ on $\chi (\mathcal {N}_\text{ h } - \mathcal {N}_\text{ p } = 0) \mathcal {F}$, we find that

$$\begin{aligned} \begin{aligned} \langle \xi , \mathcal {E}_1 \xi \rangle = \frac{1}{2N} \sum _{k \in \mathbb {Z}^3 \backslash \{ 0 \}} \hat{V} (k) \sum _{q_1,q_2 \in [B_\text{ F}^c \cap (B_\text{ F}^c + k)] \cup [B_\text{ F }\cap (B_\text{ F }-k)]} \sigma _{q_1} \sigma _{q_2} \langle \xi , a_{q_1}^* a_{q_1 - \sigma _{q_1} k} a^*_{q_2 -\sigma _2 k} a_{q_2} \xi \rangle , \end{aligned} \end{aligned}$$

where we introduced the notation $\sigma _q = 1$, if $q \in B_\text{ F}^c \cap (B_\text{ F}^c + k)$, and $\sigma _q = -1$, if $q \in B_\text{ F }\cap (B_\text{ F }+k)$. With the canonical anticommutation relations (2.1), we obtain

4.17 $$\begin{aligned} \langle \xi , \mathcal {E}_1 \xi \rangle =&- \frac{1}{2N} \sum _{k \in \mathbb {Z}^3 \backslash \{ 0 \}} \hat{V} (k) \sum _{q_1,q_2 \in [B_\text{ F}^c \cap (B_\text{ F}^c + k)] \cup [B_\text{ F }\cap (B_\text{ F }-k)]} \sigma _{q_1} \sigma _{q_2} \langle \xi , a_{q_1}^* a^*_{q_2 -\sigma _2 k} a_{q_1 - \sigma _{q_1} k} a_{q_2} \xi \rangle \nonumber \\&+ \frac{1}{2N} \sum _{k \in \mathbb {Z}^3 \backslash \{ 0 \}} \hat{V} (k) \sum _{q_1 \in [B_\text{ F}^c \cap (B_\text{ F}^c + k)] \cup [B_\text{ F }\cap (B_\text{ F }-k)]} \langle \xi , a_{q_1}^* a_{q_1} \xi \rangle \;. \end{aligned}$$

The second term can be estimated by

$$\begin{aligned} \frac{1}{2N} \sum _{k \in \mathbb {Z}^3 \backslash \{ 0 \}} \hat{V} (k) \sum _{q_1 \in [B_\text{ F}^c \cap (B_\text{ F}^c + k)] \cup [B_\text{ F }\cap (B_\text{ F }-k)]} \Vert a_{q_1} \xi \Vert ^2 \leqq C N^{-1} \Vert \mathcal {N}^{1/2} \xi \Vert ^2 \;.\end{aligned}$$

Let us focus on the first term on the right-hand side of (4.17). The first observation is that contributions with at least one of the four momenta $q_1$, $q_1 - \sigma _1 k$, $q_2$, $q_2 - \sigma _2 k$ at distances larger than $N^{-1/3 + \varepsilon }$ from the Fermi sphere, for an $0< \varepsilon < 1/3$ to be chosen later, can be bounded using a combination of $\mathcal {N}$ and of the gapped number operator $\mathcal {N}_{1/3-\varepsilon }$ defined in (4.2). In fact, considering for example the case $||q_1| - k_\text{ F }| > N^{-1/3 + \varepsilon }$ (and dropping, for an upper bound, all other restrictions on $q_1$ and $q_2$), we have

$$\begin{aligned} \begin{aligned}&\frac{1}{N} \sum _{k \in \mathbb {Z}^3 \backslash \{ 0 \}} \hat{V} (k) \sum _{q_1, q_2 \in \mathbb {Z}^3 : ||q_1| - k_\text{ F }|> N^{-1/3 + \varepsilon }} | \langle \xi , a_{q_1}^* a^*_{q_2 - \sigma _2 k} a_{q_1 - \sigma _{q_1} k} a_{q_2} \xi \rangle | \\&\quad \leqq \; \frac{1}{N} \sum _{k \in \mathbb {Z}^3 \backslash \{ 0 \}} \hat{V} (k) \left( \sum _{q_1, q_2 \in \mathbb {Z}^3 : ||q_1| - k_\text{ F }| > N^{-1/3 + \varepsilon }} \Vert a_{q_1} a_{q_2 - \sigma _2 k} (\mathcal {N}+ 1)^{-1/2} \xi \Vert ^2 \right) ^{1/2} \\&\qquad \times \left( \sum _{q_1, q_2 \in \mathbb {Z}^3} \Vert a_{q_1 - \sigma _{q_1} k} a_{q_2} (\mathcal {N}+1)^{1/2} \xi \Vert ^2 \right) ^{1/2} \\&\quad \leqq \; C N^{-1} \Vert \mathcal {N}_{1/3 - \varepsilon }^{1/2} \xi \Vert \Vert (\mathcal {N}+1)^{3/2} \xi \Vert \end{aligned} \end{aligned}$$

where we used $a^*_p \mathcal {N}= (\mathcal {N}-1) a^*_p$ for all $p\in \mathbb {Z}^3$. Thus

4.18 $$\begin{aligned} \begin{aligned} \langle \xi , \mathcal {E}_1 \xi \rangle \leqq&\; C N^{-1} \Vert \mathcal {N}^{1/2} \xi \Vert ^2 + C N^{-1} \Vert \mathcal {N}_{1/3 - \varepsilon }^{1/2} \xi \Vert \Vert (\mathcal {N}+1)^{3/2} \xi \Vert \\&+ \frac{1}{N} \sum _{k \in \mathbb {Z}^3 \backslash \{ 0 \}} \hat{V} (k) \sum _{q_1,q_2 \in A^\text{ p}_k \cup A^\text{ h}_k} | \langle \xi , a_{q_1}^* a_{q_2 - \sigma _2 k}^* a_{q_1 -\sigma _1 k} a_{q_2} \xi \rangle | \end{aligned} \end{aligned}$$

where we defined the momentum sets

$$\begin{aligned} \begin{aligned} A^\text{ p}_k&:= \left\{ q \in \mathbb {Z}^3 : k_\text{ F }< |q|< k_\text{ F }+ N^{-1/3 + \varepsilon } \quad \text{ and } \quad k_\text{ F }< |q-k|< k_\text{ F }+ N^{-1/3 + \varepsilon } \right\} \;, \\ A^\text{ h}_k&:= \left\{ q \in \mathbb {Z}^3 : k_\text{ F }- N^{-1/3 + \varepsilon }< |q| \leqq k_\text{ F }\quad \text{ and } \quad k_\text{ F }- N^{-1/3 + \varepsilon } < |q + k| \leqq k_\text{ F }\right\} \;. \end{aligned} \end{aligned}$$

Note that for $q_1 \in A^\text{ p}_k$ we have

$$\begin{aligned} \begin{aligned} k_\text{ F}^2&\leqq (q_1 - k)^2 = q_1^2 + k^2 -2 q_1 \cdot k \\ {}&\leqq (k_\text{ F }+ N^{-1/3+ \varepsilon })^2 + k^2 -2 q_1 \cdot k \leqq k_\text{ F}^2 + C N^\varepsilon + k^2 -2 q_1 \cdot k \end{aligned} \end{aligned}$$

and thus $2 q_1 \cdot k - k^2 \leqq C N^{\varepsilon }$. Inverting the roles of $q_1$ and $q_1 - k$, we also obtain $2 q_1 \cdot k -k^2 \geqq - C N^\varepsilon$. Arguing similarly for $q_1 \in A^\text{ h}_k$, we conclude that

4.19 $$\begin{aligned} -C N^\varepsilon \leqq 2 q_1 \cdot k - k^2 \leqq C N^\varepsilon \end{aligned}$$

for all $q_1 \in A^\text{ p}_k \cup A^\text{ h}_k$ (which means that the set $A^\text{ p}_k \cup A^\text{ h}_k$ is localized close to the equator of the Fermi sphere, thinking of the direction of *k* as defining the north pole).

Using the Cauchy–Schwarz inequality and $\Vert a_{q_1} \Vert _\text{ op }\leqq 1$, $\Vert a_{q_1 - \sigma _1 k} \Vert _\text{ op }\leqq 1$, we conclude that the last term on the right-hand side of (4.18) can be bounded by

4.20 $$\begin{aligned} \begin{aligned}&\frac{1}{N} \sum _{k \in \mathbb {Z}^3 \backslash \{ 0 \}} \hat{V} (k) \sum _{q_1,q_2 \in A^\text{ p}_k \cup A^\text{ h}_k} | \langle \xi , a_{q_1}^* a_{q_2 - \sigma _2 k}^* a_{q_1 -\sigma _1 k} a_{q_2} \xi \rangle | \\&\quad \leqq \frac{1}{N} \sum _{k \in \mathbb {Z}^3 \backslash \{ 0 \}} \hat{V} (k) |A^\text{ p}_k \cup A^\text{ h}_k| \Vert \mathcal {N}^{1/2} \xi \Vert ^2 \leqq \frac{\Vert \mathcal {N}^{1/2} \xi \Vert ^2}{N} \sum _{k \in \mathbb {Z}^3 \backslash \{ 0 \}} \hat{V} (k) \sum _{m=-CN^\varepsilon }^{CN^\varepsilon } |B_{m,k}| \end{aligned} \end{aligned}$$

where we defined

4.21 $$\begin{aligned} \widetilde{B}_{m}(k) := \{ q \in \mathbb {Z}^3 : k_\text{ F }- N^{-1/3 + \varepsilon } \leqq |q| \leqq k_\text{ F }+ N^{-1/3+ \varepsilon } \text { and } 2 q \cdot k - k^2 = m \} \; . \end{aligned}$$

Proceeding as in the proof of Lemma 4.3 following (4.11), we find, for $131/208< \gamma < 1$,

$$\begin{aligned} |\widetilde{B}_{m}(k)| \leqq C (N^{\varepsilon } + N^{\gamma /3}) \;. \end{aligned}$$

Inserting in (4.20) and using $\Vert \hat{V} \Vert _1 < \infty$, we obtain

$$\begin{aligned} \begin{aligned} \frac{1}{N}&\sum _{k \in \mathbb {Z}^3 \backslash \{ 0 \}} \hat{V} (k) \sum _{q_1,q_2 \in A^\text{ p}_k \cup A^\text{ h}_k} | \langle \xi , a_{q_1}^* a_{q_2 - \sigma _2 k}^* a_{q_1 -\sigma _1 k} a_{q_2} \xi \rangle | \leqq \frac{C}{N} N^{\varepsilon } (N^\varepsilon + N^{\gamma /3}) \Vert \mathcal {N}^{1/2} \xi \Vert ^2 \;. \end{aligned} \end{aligned}$$

With (4.18) this concludes the proof of Lemma 4.8. $\square$

Lemma 4.8 proves that the error term $\mathcal {E}_1$ is negligible (in the ground state and, more generally, on low-energy states with correlation energy of order $\hbar$). Together with Corollary 4.5, it also allows us to neglect the term $\mathcal {E}_2$ in (2.6). The following corollary improves [5, Lemma 9.1] in not requiring smallness of *V*, and is also simpler to prove.

### Corollary 4.9

(Coupling of bosonizable and non-bosonizable terms) Assume $\Vert \hat{V} \Vert _1 < \infty$ and $\hat{V} \geqq 0$. With the error terms $\mathcal {E}_1$, $\mathcal {E}_2$ defined as in (2.6), we have

4.22 $$\begin{aligned} \pm \mathcal {E}_2 \leqq N^\alpha \mathcal {E}_1 + C N^{-\alpha } \mathbb {H}_0 \qquad \text {for every}\, \alpha \geqq 0.\end{aligned}$$

With Lemma 4.8, we conclude that for $131/208< \gamma < 1$ and $\varepsilon > 0$ small enough (choosing $\alpha = \varepsilon /4$ in (4.22)), there exists a constant $C > 0$ such that

4.23 $$\begin{aligned} \begin{aligned} \langle \xi , (\mathcal {E}_1 + \mathcal {E}_2) \xi \rangle \geqq \;&- C N^{-1+ \varepsilon /4} \Vert (\mathcal {N}+1)^{3/2} \xi \Vert \Vert \mathcal {N}^{1/2}_{1/3-\varepsilon } \xi \Vert - C N^{5\varepsilon /4 + \gamma /3 - 1} \Vert \mathcal {N}^{1/2} \xi \Vert ^2 \\ {}&- C N^{-\varepsilon /4} \Vert \mathbb {H}_0^{1/2} \xi \Vert _2^2 \end{aligned} \end{aligned}$$

for all $$\xi \in \chi (\mathcal {N}_\text{ h } - \mathcal {N}_\text{ p } = 0) \mathcal {F}$$.

### Remark

The choice $\alpha = \varepsilon /4$ optimizes the sum of the first and the last term on the right-hand side of (4.23), counting (following the argument in the remark after Lemma 4.8) $\Vert (\mathcal {N}+1)^{3/2} \xi \Vert \lesssim N^{1/2}$, $\Vert \mathcal {N}^{1/2}_{1/3-\varepsilon } \xi \Vert \lesssim N^{1/6-\varepsilon /2}$, and $\Vert \mathbb {H}^{1/2}_0 \xi \Vert ^2 \lesssim N^{-1/3}$. The second term on the right-hand side of (4.23) is of lower order if $\gamma$ is chosen small enough.

### Proof of Corollary 4.9

By Cauchy–Schwarz, Corollary 4.5, and $\Vert \hat{V} \Vert _1 < \infty$, we find

$$\begin{aligned} \pm \mathcal {E}_2 \leqq N^\alpha \mathcal {E}_1 + N^{-\alpha - 1} \sum _{k \in \mathbb {Z}^3} \hat{V} (k) b^* (k) b(k) \leqq N^\alpha \mathcal {E}_1 + C N^{-\alpha } \mathbb {H}_0 \; . \end{aligned}$$

$$\square $$

## Patch Decomposition and Almost Bosonic Operators

The bounds in last section allow us to approximate the correlation Hamiltonian (2.4) by $\mathbb {H}_0 + Q_\text{ B }$, with $\mathbb {H}_0$ and $Q_\text{ B }$ defined in (2.5). The term $Q_\text{ B }$, arising from the interaction, is quadratic in the particle–hole pair creation and annihilation operators $b^* (k)$, *b*(*k*). It turns out that, on states with few excitations of the Fermi ball, the operators $b^* (k)$ and *b*(*k*) satisfy approximately bosonic commutation relations.

In order to express also the kinetic energy $\mathbb {H}_0$ in terms of almost bosonic creation and annihilation operators, we have to decompose a layer around the Fermi sphere $\partial B_\text{ F }$ into *M* patches $\{ B_\alpha \}_{\alpha =1}^M$, for the number of patches $M \in \mathbb {N}$ to be chosen as a function of *N* at the end of the paper. Such a decomposition has been constructed in [4]. One starts by decomposing a half sphere in *M*/2 patches. The sidelengths of the patches are comparable (they are both of order $N^{1/3} / M^{1/2}$). The patches have thickness

$$\begin{aligned} 1 \ll 2R \ll N^{1/3}\end{aligned}$$

in the radial direction (later we will impose stronger conditions). Furthermore, the patches are disjoint and separated by corridors, larger than *R*. We denote by $\omega _\alpha$ the center of the patch $B_\alpha$. Finally, the patch decomposition of the first half sphere is mirrored by the map $k \mapsto -k$ onto the other half sphere. The construction is so that the area of the radial projection $p_\alpha$ of the patch $B_\alpha$ on the unit sphere $\mathbb {S}_2$ has area $4\pi / M$, up to corrections of order $N^{-1/3} M^{-1/2}$, and diameter bounded by $C/\sqrt{M}$, for all $\alpha = 1, \dots , M$; see [4, Section 3.2] for the details.

For fixed $k \in \mathbb {Z}^3$ with $|k| < R$, we are going to exclude patches in a small strip around the equator (thinking of the direction of *k* as defining the north direction) of the Fermi sphere. More precisely, for $0< \delta < 1/6$, we define $\mathcal {I}_k := \mathcal {I}_k^+ \cup \mathcal {I}_k^-$, with

5.1 $$\begin{aligned} \begin{aligned} \mathcal {I}_k^+&:= \{ \alpha \in \{ 1, \dots , M \} : k \cdot \hat{\omega }_\alpha \geqq N^{-\delta } \} \;,\\ \mathcal {I}_k^-&:= \{ \alpha \in \{ 1, \dots , M \} : k \cdot \hat{\omega }_\alpha \leqq - N^{-\delta } \} \;. \end{aligned} \end{aligned}$$

Given $k \in \mathbb {Z}^3$, $|k| < R$ and $\alpha \in \mathcal {I}_k^+$, we introduce the particle–hole pair creation operator

5.2 $$\begin{aligned} b^*_\alpha (k) := \frac{1}{n_\alpha (k)} \sum _{\begin{array}{c} p:p \in B_\text{ F}^c \cap B_\alpha \\ p-k\in B_\text{ F }\cap B_\alpha \end{array}} a^*_p a^*_{p-k} \end{aligned}$$

with the normalization constant

$$\begin{aligned} n_\alpha (k)^2 := \sum _{\begin{array}{c} p:p \in B_\text{ F}^c \cap B_\alpha \\ p-k\in B_\text{ F }\cap B_\alpha \end{array}} 1 \end{aligned}$$

counting the number of particle–hole pairs of relative momentum *k* in $B_\alpha$. The normalization constant $n_\alpha (k)$ should be large (the more summands contribute to (5.2), the less the $b^*$-operators are affected by the Pauli principle, and the more bosonic they behave). The following lemma is a variation of [4, Prop. 3.1] and [5, Lemma 5.1].

### Lemma 5.1

(Number of pairs per patch) Assume that $N^{2\delta } R^2 \ll M \ll N^{\frac{2}{3}-2\delta } R^{-4}$. Then for all $k \in \mathbb {Z}^3$ with $|k| < R$ and $\alpha \in \mathcal {I}_k$, we have

$$\begin{aligned} n_\alpha (k)^2 = \frac{4\pi k_\text{ F}^2}{M} |k \cdot {\hat{\omega }}_\alpha |\left( 1 + o(1) \right) \;. \end{aligned}$$

### Proof

The proof follows the argument given in [4, Section 6]; only the control of the error terms needs to be refined in two respects.

**Fig. 2:**
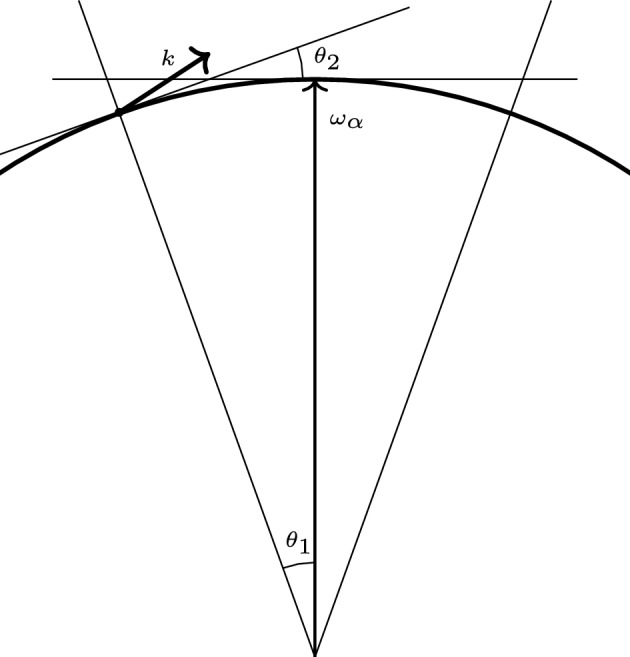
Illustration for the condition $N^{2\delta }R^2 \ll M$ of Lemma 5.1. The angle between patch center and patch boundary is $\theta _1 \sim 1/\sqrt{M}$. The angle between the tangent at the center and at the boundary is $\theta _2 = \theta _1$ by elementary geometry. We know $k\cdot \hat{\omega }_\alpha \geqq N^{-\delta }$ by definition of $\mathcal {I}_k$. This means that the angle between *k* and the tangent at the center (being perpendicular to $\omega _\alpha$) is at least of order $\sim N^{-\delta }/R$. To have *k* pointing from the inside to the outside of the Fermi ball even at the boundary we need $N^{-\delta }/R \gg 1/\sqrt{M}$

First, in order for the vector *k* to point from inside the Fermi ball to outside the Fermi ball even at the boundaries of the patch, we need $N^{2\delta } R^2 \ll M$, as can be verified by elementary geometry. This condition is illustrated in Fig. 2.

Second, the error term arising from the loss of particle–hole pairs near the boundary of the patch (thus proportional to the number of pairs in the patch of thickness $|k|\leqq R$ not more than a distance $|k|\leqq R$ from the patch boundary on the Fermi sphere) implies

5.3 $$\begin{aligned} n_\alpha (k)^2 = \frac{4\pi k_{\text{ F }}^2}{M} |k \cdot \hat{\omega }_\alpha |+ \mathcal {O}\left( \frac{N^{1/3}}{\sqrt{M}} |k|^2 \right) = \frac{4\pi k_{\text{ F }}^2}{M} |k \cdot \hat{\omega }_\alpha |\left( 1 + \mathcal {O}\left( \frac{\sqrt{M} |k|^2}{N^{1/3} |k \cdot \hat{\omega }_\alpha |}\right) \right) \;. \end{aligned}$$

The error term becomes *o*(1) since by assumption $\sqrt{M} R^2 N^{-1/3} N^\delta \ll 1$. $\square$

It will be convenient to combine modes associated with *k* and $-k$. To this end, we set

5.4 $$\begin{aligned} c_\alpha ^* (k) := \left\{ \begin{array}{ll} b^*_\alpha (k) &{}\quad \text {for}\, \alpha \in \mathcal {I}_k^+ \\ b_\alpha ^* (-k) &{}\quad \text {for}\, \alpha \in \mathcal {I}_k^- \end{array} \right. \end{aligned}$$

for every $k \in \Gamma ^{\text{ nor }}$. Here, we introduce the notation

5.5 $$\begin{aligned} \Gamma ^{\text{ nor }}&:=\Big \{ k = (k_1, k_2, k_3) \in \mathbb {Z}^3 \text { with } |k| < R: \; k_3> 0 \text { or } (k_3 = 0 \text { and } k_2> 0) \nonumber \\&\quad \text { or } (k_3 = k_2= 0 \text { and } k_1 > 0) \Big \} \end{aligned}$$

so that $\Gamma ^{\text{ nor }}\cap (-\Gamma ^{\text{ nor }}) = \emptyset$ and $\Gamma ^{\text{ nor }}\cup (-\Gamma ^{\text{ nor }}) = B_R (0) \backslash \{ 0 \}$. Note that compared to [5], in the definition of $\Gamma ^{\text{ nor }}$ we replaced the restriction $k \in {\text {supp}}\hat{V}$ by $|k|< R$, with the parameter *R* to be optimized at the end.

Our analysis is based on the observation that the pair operators $c_\alpha ^* (k)$ and $c_\alpha (k)$ behave approximately as bosonic creation and annihilation operators, on states with few excitations. This is established by the following lemma, taken from [4, Lemma 4.1] and [5, Lemma 5.2].

### Lemma 5.2

(Approximate bosonic CCR) Let $k, \ell \in \Gamma ^{\text{ nor }}$. Let $\alpha \in \mathcal {I}_k$ and $\beta \in \mathcal {I}_\ell$. Then

5.6 $$\begin{aligned} {[}c_\alpha (k),c_\beta ( \ell )] = 0 = [c^*_\alpha (k),c^*_\beta (\ell )]\;, \quad [c_\alpha (k),c^*_\beta (\ell )] = \delta _{\alpha ,\beta }\big ( \delta _{k,\ell } + \mathcal {E}_\alpha (k,\ell ) \big )\;, \end{aligned}$$

where the error operator $\mathcal {E}_\alpha (k,\ell )$ is controlled by the bounds

5.7 $$\begin{aligned} \sum _{\alpha \in \mathcal {I}_k \cap \mathcal {I}_\ell } |\mathcal {E}_\alpha (k,\ell ) |^2 \leqq C (M N^{-\frac{2}{3}+\delta } \mathcal {N})^2 \end{aligned}$$

and

5.8 $$\begin{aligned} \sum _{\alpha \in \mathcal {I}_k \cap \mathcal {I}_\ell } \Vert \mathcal {E}_\alpha (k,\ell ) \psi \Vert \leqq C M^{\frac{3}{2}}N^{-\frac{2}{3}+\delta } \Vert \mathcal {N}\psi \Vert \qquad \text {for all}\, \psi \in \mathcal {F}. \end{aligned}$$

Another important property of the operators $c_\alpha ^* (k)$ and $c_\alpha (k)$ is that they can be controlled in terms of the gapped number of particles operator $\mathcal {N}_\delta$ introduced in (4.2), with $\delta > 0$ the parameter introduced in (5.1) to exclude a strip around the equator of the Fermi sphere in the definition of the sets $\mathcal {I}_k$. The point is that, since we are away from the equator, *k* has a component orthogonal to the Fermi sphere, which makes sure that the momentum of either the particle or of the hole annihilated by $c_\alpha (k)$ is at least at distance $N^{-\delta }$ from the Fermi sphere. More precisely, we have the following lemma, whose proof can be found in [5, Lemmas 5.3 and 5.4] (the first estimate in (5.10) and in (5.12) are not stated explicitly in [5, Lemmas 5.3 and 5.4] but can be proven like the second bounds):

### Lemma 5.3

(Bounds on pair operators) Assume $M \gg R^2 N^{2\delta }$ and $R \ll N^{1/6-\delta /2}$. For all $k \in \Gamma ^{\text{ nor }}$ we have

5.9 $$\begin{aligned} \sum _{\alpha \in \mathcal {I}_k} c_\alpha ^* (k) c_\alpha (k) \leqq \mathcal {N}_\delta \;. \end{aligned}$$

Moreover, for any $f \in \ell ^2 (\mathcal {I}_k)$,

5.10 $$\begin{aligned} \Big \Vert \sum _{\alpha \in \mathcal {I}_k} f_\alpha c_\alpha (k) \psi \Big \Vert \leqq \Vert f \Vert _2 \Vert \mathcal {N}_\delta ^{1/2} \psi \Vert \; , \ \Big \Vert \sum _{\alpha \in \mathcal {I}_k} f_\alpha c^*_\alpha (k) \psi \Big \Vert \leqq \Vert f \Vert _2 \Vert (\mathcal {N}_\delta +1)^{1/2} \psi \Vert \;. \end{aligned}$$

For $k \in \Gamma ^{\text{ nor }}$, $\alpha \in \mathcal {I}_k$ and $g : \mathbb {Z}^3 \times \mathbb {Z}^3 \rightarrow \mathbb {R}$, we define the weighted pair operator

$$\begin{aligned} c_\alpha ^g (k) := \frac{1}{n_\alpha (k)} \sum _{\begin{array}{c} p : p \in B_\text{ F}^c \cap B_\alpha \\ p- \sigma _\alpha k \in B_\text{ F }\cap B_\alpha \end{array}} g(p,k) a_{p- \sigma _\alpha k} a_p \end{aligned}$$

with $\sigma _\alpha = 1$ if $\alpha \in \mathcal {I}_k^+$, and $\sigma _\alpha = -1$ if $\alpha \in \mathcal {I}_k^-$. Similarly to (5.9) and (5.10), we find that

$$\begin{aligned} \sum _{\alpha \in \mathcal {I}_k} c_\alpha ^{g*} (k) c^g_\alpha (k) \leqq \Vert g \Vert _\infty ^2 \mathcal {N}_\delta \;.\end{aligned}$$

Furthermore,

5.11 $$\begin{aligned} \begin{aligned} \sum _{\alpha \in \mathcal {I}_k} \Big \Vert c^g_\alpha (k) \psi \Big \Vert&\leqq CM^{1/2} \Vert g \Vert _\infty \Vert \mathcal {N}_\delta ^{1/2} \psi \Vert ^2 \;, \\ \sum _{\alpha \in \mathcal {I}_k} \Big \Vert c^{g*}_\alpha (k) \psi \Big \Vert&\leqq C M^{1/2} \Vert g \Vert _\infty \Vert (\mathcal {N}_\delta +M)^{1/2} \psi \Vert ^2 \end{aligned} \end{aligned}$$

and, for $$f \in \ell ^2(\mathcal {I}_{k})$$,

5.12 $$\begin{aligned} \begin{aligned} \Big \Vert \sum _{\alpha \in \mathcal {I}_k} f_\alpha c^g_\alpha (k) \psi \Big \Vert&\leqq \Vert f \Vert _2 \Vert g \Vert _\infty \Vert \mathcal {N}_\delta ^{1/2} \psi \Vert ^2 \;, \\ \Big \Vert \sum _{\alpha \in \mathcal {I}_k} f_\alpha c^{g*}_\alpha (k) \psi \Big \Vert&\leqq \Vert f \Vert _2 \Vert g \Vert _\infty \Vert (\mathcal {N}_\delta +1)^{1/2} \psi \Vert ^2 \;. \end{aligned} \end{aligned}$$

## Reduction to an Almost Bosonic Quadratic Hamiltonian

Comparing (2.7) with (5.4), we find that

$$\begin{aligned} b^* (k) \simeq \sum _{\alpha \in \mathcal {I}_k^+} n_\alpha (k) c^*_\alpha (k)\; , \qquad b^*(-k) \simeq \sum _{\alpha \in \mathcal {I}_k^-} n_\alpha (k) c^*_\alpha (k) \end{aligned}$$

for all $k \in \Gamma ^{\text{ nor }}$ (these are only approximate decompositions since, on the r. h. s., pairs in corridors and close to the equator are missing). Inserting this decomposition in (2.5) we find the following approximation for $Q_\text{ B }$, quadratic in *c*- and $c^*$-operators:

6.1 $$\begin{aligned} \begin{aligned} Q_\text{ B}^R =&\frac{1}{N} \sum _{k \in \Gamma ^{\text{ nor }}} \hat{V} (k) \Bigg ( \sum _{\alpha , \beta \in \mathcal {I}_k^+} n_\alpha (k) n_\beta (k) c_\alpha ^* (k) c_\beta (k) + \sum _{\alpha , \beta \in \mathcal {I}_k^-} n_\alpha (k) n_\beta (k) c_\alpha ^* (k) c_\beta (k) \\&+ \sum _{\alpha \in \mathcal {I}_k^+ , \beta \in \mathcal {I}_k^-} n_\alpha (k) n_\beta (k) c^*_\alpha (k) c^*_\beta (k) + \sum _{\alpha \in \mathcal {I}_k^-, \beta \in \mathcal {I}_k^+} n_\alpha (k) n_\beta (k) c_\alpha (k) c_\beta (k) \Bigg )\,. \end{aligned} \end{aligned}$$

The difference between $Q_\text{ B }$ and $Q_\text{ B}^R$ is estimated in the following lemma, which we take from [5, Lemma 4.1]. Compared to [5], here we only need to compare $Q_\text{ B }$ with $Q_\text{ B}^R$ since we already controlled $\mathcal {E}_2$ in Corollary 4.9; therefore the bound also does not use $\mathcal {E}_1$.

### Lemma 6.1

(Removing corridors and removing patches near the equator) Assume that $\sum _{k \in \mathbb {Z}^3} |\hat{V} (k)||k| < \infty$. Then there exists $C > 0$ such that for all $\psi \in \mathcal {F}$ we have

$$\begin{aligned} \begin{aligned} |\langle \psi , \left( Q_\text{ B } - Q_\text{ B}^R \right) \psi \rangle |\leqq C (N^{-\delta /2} + R^{1/2} M^{1/4} N^{-1/6+ \delta /2} + R^{-1 /2}) \langle \psi ,(\mathbb {H}_0 + \hbar ) \psi \rangle \;. \end{aligned} \end{aligned}$$

### Proof

We consider the difference

$$\begin{aligned} b(k) - \sum _{\alpha \in \mathcal {I}_k^+} n_\alpha (k) c_\alpha (k) = \sum _{p \in U_k} a_{p-k} a_p \end{aligned}$$

where $U_k$ consists of all momenta $p \in B_\text{ F}^c$ with $p-k \in B_\text{ F }$ that do not belong to any patch. For $|k| < R$, we bound

$$\begin{aligned} \Big \Vert \Big ( b(k) - \sum _{\alpha \in \mathcal {I}_k^+} n_\alpha (k) c_\alpha (k) \Big ) \psi \Big \Vert \leqq \sum _{p \in Y_k} \Vert a_{p-k} a_p \psi \Vert + \sum _{p \in U_k \backslash Y_k} \Vert a_{p-k} a_p \psi \Vert \end{aligned}$$

with

$$\begin{aligned} Y_k := \{ p \in U_k : e(p ) + e(p-k) \leqq 4 N^{-1/3-\delta } \} \end{aligned}$$

containing pairs close to the equator. Proceeding as in the proof of [5, Lemma 4.1] and using (4.6), we obtain

$$\begin{aligned} \sum _{p \in Y_k} \Vert a_{p-k} a_p \psi \Vert \leqq C N^{1/2-\delta /2} \Vert \mathbb {H}_0^{1/2} \psi \Vert \end{aligned}$$

and (again under the assumption that $|k| < R$)

$$\begin{aligned} \sum _{p \in U_k \backslash Y_k} \Vert a_{p-k} a_p \psi \Vert \leqq C |k|^{1/2} R^{1/2} M^{1/4} N^{1/3+\delta /2} \Vert \mathbb {H}_0^{1/2} \psi \Vert \;. \end{aligned}$$

Here we estimated $|U_k \backslash Y_k| \leqq C R |k| N^{1/3} M^{1/2}$ (for $|k| < R$, the set $U_k \backslash Y_k$ contains momenta $p \in \mathbb {Z}^3$ localized in a shell of thickness |*k*| around the Fermi sphere, so that either the projection of *p* or the projection of $p-k$ onto the Fermi sphere falls in corridors of size *R* between patches). For $|k| > R$, on the other hand, we use Corollary 4.5. We conclude that

$$\begin{aligned} \begin{aligned}&\Big \Vert \Big ( b(k) - \sum _{\alpha \in \mathcal {I}_k^+} n_\alpha (k) c_\alpha (k) \Big ) \psi \Big \Vert \\ {}&\quad \leqq C \left( N^{1/2-\delta /2} + |k|^{1/2} R^{1/2} M^{1/4} N^{1/3+\delta /2} + \chi (|k| > R) N^{1/2} \right) \Vert \mathbb {H}_0^{1/2} \psi \Vert \;. \end{aligned} \end{aligned}$$

Proceeding as in the last part of the proof of [5, Lemma 4.1], using Corollary 4.5 and the assumption $\sum _{k \in \mathbb {Z}^3} \hat{V} (k) |k| < \infty$, we arrive at the intended bound. $\square$

To understand how the kinetic energy $\mathbb {H}_0$, defined in (2.5), can be expressed through the patch-wise particle–hole creation and annihilation operators, we compute the commutator

$$\begin{aligned} \begin{aligned} {[}\mathbb {H}_0 , c_\alpha ^* (k)]&= \left[ \sum _{q \in \mathbb {Z}^3} e(q) a_q^* a_q , \frac{1}{n_\alpha (k)} \sum _{p \in B_\text{ F}^c \cap (B_\text{ F }+k) \cap B_\alpha } a_p^* a_{p-k}^* \right] \\ {}&= \frac{1}{n_\alpha (k)} \sum _{ p \in B_\text{ F}^c \cap (B_\text{ F }+k) \cap B_\alpha } (e(p) + e(p-k)) a_p^* a_{p-k}^* \;. \end{aligned} \end{aligned}$$

With $e(p) + e(p-k) = \hbar ^2 p^2 - \hbar ^2 (p-k)^2 \simeq 2 \hbar \kappa |k \cdot \hat{\omega }_\alpha |$ (with $\hat{\omega }_\alpha = \omega _\alpha / |\omega _\alpha |$ the normalized vector pointing to the center of the $\alpha$-th patch), we obtain that

6.2 $$\begin{aligned} {[}\mathbb {H}_0 , c_\alpha ^* (k)] \simeq 2\hbar \kappa \, |k \cdot \hat{\omega }_\alpha | c_\alpha ^* (k), \end{aligned}$$

which suggests that, in a sense to be made precise,

6.3 $$\begin{aligned} \mathbb {H}_0 \simeq 2 \kappa \hbar \sum _{k \in \Gamma ^{\text{ nor }}} \sum _{\alpha =1}^M |k \cdot \hat{\omega }_\alpha | \, c_\alpha ^* (k) c_\alpha (k) = : \mathbb {D}_\text{ B } \;.\end{aligned}$$

Based on this heuristic observation, we expect that the correlation Hamiltonian (2.4) can be approximated by

6.4 $$\begin{aligned} \mathcal {H}_\text{ corr } \simeq \mathbb {D}_\text{ B } + Q^R_B = \sum _{k \in \Gamma ^{\text{ nor }}} 2\hbar \kappa |k| h_\text {eff} (k) \end{aligned}$$

with the quadratic (in *c*- and $c^*$-operators) expression

6.5 $$\begin{aligned} h_\text {eff} (k) = \sum _{\alpha ,\beta \in \mathcal {I}_k} \left( (D(k) + W(k))_{\alpha ,\beta } c_\alpha ^* (k) c_\beta (k) + \frac{1}{2} \widetilde{W} (k)_{\alpha ,\beta } \big ( c_\alpha ^* (k) c_\beta ^* (k) + c_\beta (k) c_\alpha (k) \big ) \right) \end{aligned}$$

where *D*(*k*), *W*(*k*), and $\widetilde{W} (k)$ are $|\mathcal {I}_k| \times |\mathcal {I}_k|$ real symmetric matrices with entries

6.6 $$\begin{aligned} \begin{aligned} D(k)_{\alpha ,\beta }&= \delta _{\alpha ,\beta } |\hat{k} \cdot \hat{\omega }_\alpha | \;, \quad \text {for all } \alpha , \beta \in \mathcal {I}_k \\ W(k)_{\alpha , \beta }&= \frac{\hat{V} (k)}{2\hbar \kappa N |k|} \times \left\{ \begin{array}{ll} n_\alpha (k) n_\beta (k) \quad &{}\text { if}\, \alpha , \beta \in \mathcal {I}_k^+\, \text {or}\, \alpha ,\beta \in \mathcal {I}_k^-\\ 0 \quad &{}\text { otherwise}\,, \end{array} \right. \\ \widetilde{W} (k)_{\alpha ,\beta }&= \frac{\hat{V} (k)}{2\hbar \kappa N |k|} \times \left\{ \begin{array}{ll} 0 \quad &{}\text { if}\, \alpha , \beta \in \mathcal {I}_k^+\, \text {or}\, \alpha ,\beta \in \mathcal {I}_k^- \\ n_\alpha (k) n_\beta (k) \quad &{}\text { otherwise}\,. \end{array} \right. \end{aligned} \end{aligned}$$

## Approximate Bogoliubov Transformations

If the *c*- and $c^*$-operators were exactly bosonic, we could write

$$\begin{aligned} h_\text {eff} (k) = \mathbb {H}- \frac{1}{2} {\text {tr}}\, (D (k) +W (k)) \end{aligned}$$

with the quadratic Hamiltonian (in the following discussion we omit the fixed argument *k*)

7.1 $$\begin{aligned} \mathbb {H}:= \frac{1}{2} ((c^*)^T , c^T) \left( \begin{array}{ll} D+W &{} \widetilde{W} \\ \widetilde{W} &{} D+W \end{array} \right) \left( \begin{array}{c} c \\ c^* \end{array} \right) \;. \end{aligned}$$

Introducing the $|\mathcal {I}_k | \times |\mathcal {I}_k |$ matrix

$$\begin{aligned} E := \left[ (D + W - \widetilde{W})^{1/2} (D + W + \widetilde{W}) (D + W -\widetilde{W})^{1/2} \right] ^{1/2} \, \end{aligned}$$

and setting $S_1 := (D+W-\widetilde{W})^{1/2} E^{-1/2}$, $S_2 := (D+W-\widetilde{W})^{-1/2} E^{1/2}$ (so that $S_1 S_2^T = S_2 S_1^T = 1$) and

7.2 $$\begin{aligned} S := \left( \begin{array}{ll} S_1 &{} 0 \\ 0 &{} S_2 \end{array} \right) \end{aligned}$$

we can decompose

7.3 $$\begin{aligned} \left( \begin{array}{ll} D+W &{} \widetilde{W} \\ \widetilde{W} &{} D+W \end{array} \right) = \left( \begin{array}{ll} \frac{S_1 + S_2}{2} &{} \frac{S_1 - S_2}{2} \\ \frac{S_ 1 - S_2}{2} &{} \frac{S_1 + S_2}{2} \end{array} \right) ^T \left( \begin{array}{ll} E &{} 0 \\ 0 &{} E \end{array} \right) \left( \begin{array}{ll} \frac{S_1 + S_2}{2} &{} \frac{S_1 - S_2}{2} \\ \frac{S_ 1 - S_2}{2} &{} \frac{S_1 + S_2}{2} \end{array} \right) \;.\end{aligned}$$

Using the polar decomposition $S_1 = O |S_1|$ with an orthogonal matrix *O* and the positive matrix $|S_1| = (S_1^T S_1)^{1/2}$ we obtain $S_2 = O |S_1|^{-1}$ from $S_2 S_1^T = 1$. Moreover, $|S_1^T| = O |S_1| O^T$ and thus $S_1 = |S_1^T| O$, $S_2 = |S_1^T| O$ and, from (7.3),

$$\begin{aligned} \begin{aligned} \left( \begin{array}{ll} D+W &{} \widetilde{W} \\ \widetilde{W} &{} D+W \end{array} \right)&= \left( \begin{array}{ll} \frac{|S_1^T| + |S_1^T|^{-1}}{2} &{} \frac{|S^T_1| - |S_1^T|^{-1}}{2} \\ \frac{|S^T_ 1| - |S^T_1|^{-1}}{2} &{} \frac{|S_1^T| + |S_1^T|^{-1}}{2} \end{array} \right) \left( \begin{array}{ll} O &{} 0 \\ 0 &{} O \end{array} \right) \left( \begin{array}{ll} E &{} 0 \\ 0 &{} E \end{array} \right) \\&\quad \times \left( \begin{array}{ll} O &{} 0 \\ 0 &{} O \end{array} \right) ^T \left( \begin{array}{ll} \frac{|S_1^T| + |S_1^T|^{-1}}{2} &{} \frac{|S^T_1| - |S_1^T|^{-1}}{2} \\ \frac{|S^T_ 1| - |S^T_1|^{-1}}{2} &{} \frac{|S_1^T| + |S_1^T|^{-1}}{2} \end{array} \right) \;. \end{aligned} \end{aligned}$$

Defining

$$\begin{aligned} K := \log |S_1^T|\end{aligned}$$

we obtain

7.4 $$\begin{aligned} \begin{aligned} \left( \begin{array}{ll} D+W &{} \widetilde{W} \\ \widetilde{W} &{} D+W \end{array} \right)&= \left( \begin{array}{ll} \cosh (K) &{} \sinh (K) \\ \sinh (K) &{} \cosh (K) \end{array} \right) \left( \begin{array}{ll} O &{} 0 \\ 0 &{} O \end{array} \right) \left( \begin{array}{ll} E &{} 0 \\ 0 &{} E \end{array} \right) \\&\quad \times \left( \begin{array}{ll} O &{} 0 \\ 0 &{} O \end{array} \right) ^T \left( \begin{array}{ll} \cosh (K) &{} \sinh (K) \\ \sinh (K) &{} \cosh (K) \end{array} \right) \;. \end{aligned} \end{aligned}$$

Hence, a symplectic conjugation of the $2|\mathcal {I}_k| \times 2 |\mathcal {I}_k|$ matrix defining the quadratic Hamiltonian (7.1) is sufficient to obtain a block-diagonal matrix (with $|\mathcal {I}_k| \times |\mathcal {I}_k|$ blocks $O E O^T$) corresponding to a “diagonal” quadratic Hamiltonian in the sense of containing only terms of the form $c^* c$ and none of the form $c^* c^*$ or *cc*.

However, it will be important to further transform the block-diagonal matrix as to make the resulting quadratic Hamiltonian comparable with the bosonic kinetic energy $\mathbb {D}_\text{ B }$, defined in (6.3). To reach this goal we have to look more closely at *E*, decomposing it further into blocks associated to the index sets $\mathcal {I}_k^+$ and $\mathcal {I}_k^-$ (associated with patches in the north and south hemisphere, respectively). Note that $I = |\mathcal {I}_k^+| = |\mathcal {I}_k^-| = |\mathcal {I}_k| /2$. With (6.6) we write

7.5 $$\begin{aligned} D = \left( \begin{array}{ll} d &{} 0 \\ 0 &{} d \end{array} \right) , \quad W = \left( \begin{array}{ll} b &{} 0 \\ 0 &{} b \end{array} \right) , \quad \widetilde{W} = \left( \begin{array}{ll} 0 &{} b \\ b &{} 0 \end{array} \right) \end{aligned}$$

where $d = \text {diag} \{ u_\alpha ^2 , \alpha = 1, \dots , I \}$ and $b = g |v \rangle \langle v |$. Here we introduced

$$\begin{aligned} g = \frac{\kappa }{2} \hat{V} (k)\; , \quad u_\alpha = |\hat{k} \cdot \hat{\omega }_\alpha |^{1/2}\;, \quad v_\alpha = \frac{\hbar }{\kappa \sqrt{|k|}} n_\alpha (k) \qquad \text{ for } \alpha = 1, \dots , I\;. \end{aligned}$$

It will play an important role in the proof of Lemma 7.2 that, as a consequence of (5.1) and Lemma 5.1, we have

7.6 $$\begin{aligned} N^{-\delta } \leqq u_\alpha ^2 \leqq 1\;, \qquad |v_\alpha | \leqq C \frac{u_\alpha }{M^{1/2}} \end{aligned}$$

which implies $\Vert v\Vert \leqq C$ and $\Vert d^{-1/2} v \Vert \leqq C$.

To block-diagonalize *E* (with respect to the decomposition $\mathcal {I}_k = \mathcal {I}_k^+ \cup \mathcal {I}_k^-$), we introduce

7.7 $$\begin{aligned} U := \frac{1}{\sqrt{2}} \left( \begin{array}{ll} \mathbb {I}&{} \mathbb {I}\\ \mathbb {I}&{} -\mathbb {I}\end{array} \right) \end{aligned}$$

(where $\mathbb {I}$ is the $I\times I$ identity matrix) and observe that

$$\begin{aligned} U^T (D+W+\widetilde{W}) U = \left( \begin{array}{ll} d+2b &{} 0 \\ 0 &{} d \end{array} \right) \; , \qquad U^T (D+W - \widetilde{W} ) U = \left( \begin{array}{ll} d &{} 0 \\ 0 &{} d+ 2b \end{array} \right) \;. \end{aligned}$$

This implies that

7.8 $$\begin{aligned} U^T E U = \left( \begin{array}{ll} [d^{1/2} (d+2b) d^{1/2} ]^{1/2} &{} 0 \\ 0 &{} [ (d+2b)^{1/2} d (d+2b)^{1/2} ]^{1/2} \end{array} \right) \;. \end{aligned}$$

The upper-left entry is clearly larger than the operator *d*. It seems more difficult to compare the lower-right entry with *d* (thus, it seems difficult to compare $U^T E U$ with *D*). To solve this problem, we define the $I \times I$ matrix $X := (d+2b)^{1/2} d^{1/2}$ and consider its polar decomposition $X = A P$, with *A* orthogonal and $P := (X^* X)^{1/2}$. Then, from (7.8), we have

$$\begin{aligned} \begin{aligned} U^T E U&= \left( \begin{array}{ll} (X^* X)^{1/2} &{} 0 \\ 0 &{} (X X^*)^{1/2} \end{array} \right) \\ {}&= \left( \begin{array}{ll} P &{} 0 \\ 0 &{} A P A^T \end{array} \right) = \left( \begin{array}{ll} 1 &{} 0 \\ 0 &{} A \end{array} \right) \left( \begin{array}{ll} P &{} 0 \\ 0 &{} P \end{array} \right) \left( \begin{array}{ll} 1 &{} 0 \\ 0 &{} A^T \end{array} \right) \;. \end{aligned} \end{aligned}$$

Using the easily-checked invariance of the matrix with blocks *P* on the diagonal with respect to conjugation with *U* we conclude that

$$\begin{aligned} E = \widetilde{O} \widetilde{P} \widetilde{O}^T\;, \end{aligned}$$

where we defined

7.9 $$\begin{aligned} \widetilde{O} := U \left( \begin{array}{ll} 1 &{} 0 \\ 0 &{} A \end{array} \right) U^T \;, \qquad \widetilde{P} := \left( \begin{array}{ll} P &{} 0 \\ 0 &{} P \end{array} \right) \;.\end{aligned}$$

Inserting in (7.4), we arrive at

7.10 $$\begin{aligned} \left( \begin{array}{ll} D+W &{} \widetilde{W} \\ \widetilde{W} &{} D+W \end{array} \right)&= \left( \begin{array}{ll} \cosh (K) &{} \sinh (K) \\ \sinh (K) &{} \cosh (K) \end{array} \right) \left( \begin{array}{ll} O &{} 0 \\ 0 &{} O \end{array} \right) \left( \begin{array}{ll} \widetilde{O} &{} 0 \\ 0 &{} \widetilde{O} \end{array} \right) \left( \begin{array}{ll} \widetilde{P} &{} 0 \\ 0 &{} \widetilde{P} \end{array} \right) \nonumber \\&\quad \times \left( \begin{array}{ll} \widetilde{O} &{} 0 \\ 0 &{} \widetilde{O} \end{array} \right) ^T \left( \begin{array}{ll} O &{} 0 \\ 0 &{} O \end{array} \right) ^T \left( \begin{array}{ll} \cosh (K) &{} \sinh (K) \\ \sinh (K) &{} \cosh (K) \end{array} \right) \;. \end{aligned}$$

If the *c*- and $c^*$-operators were exactly bosonic we could therefore bring the quadratic operator (7.1) into a diagonal form comparable to the bosonic kinetic energy $\mathbb {D}_\text{ B }$ by means of the two Bogoliubov transformations^2^

7.11 $$\begin{aligned} \begin{aligned} T&= \exp \left( \frac{1}{2} \sum _{k \in \Gamma ^{\text{ nor }}} \sum _{\alpha ,\beta \in \mathcal {I}_k} K (k)_{\alpha ,\beta } \, c_\alpha ^* (k) c_\beta ^* (k) - \text {h.c.} \right) \;, \\ Z&= \exp \left( \sum _{k \in \Gamma ^{\text{ nor }}} \sum _{\alpha ,\beta \in \mathcal {I}_k} L_{\alpha ,\beta } (k) \, c_\alpha ^* (k) c_\beta (k) \right) \;, \end{aligned} \end{aligned}$$

where (re-inserting now the dependence on *k* in the notation) we introduced the matrix

7.12 $$\begin{aligned} L(k) := \log \left( O(k) \widetilde{O}(k) \right) \;. \end{aligned}$$

Recall that *O*(*k*) and $\widetilde{O} (k)$ are orthogonal matrices, that is, all their eigenvalues are on the unit circle. The function $\log$ denotes an arbitrary branch of the complex logarithm with $\text {Im}\,\log 1 = 0$. The matrix *L*(*k*) is by definition antisymmetric, so that *Z* is a unitary operator on Fock space. If the *c*- and $c^*$-operators were exactly bosonic, we would find

7.13 $$\begin{aligned} Z^* T^* \mathbb {H}T Z = \frac{1}{2} \sum _{\alpha , \beta \in \mathcal {I}_k} \widetilde{P}_{\alpha , \beta } \left( c_\alpha ^* (k) c_\beta (k) + \delta _{\alpha ,\beta } \right) \;. \end{aligned}$$

Recall that ${\text {tr}}\tilde{P} = {\text {tr}}E$. Since $P = (X^* X)^{1/2} = [ d^{1/2} (d+2b) d^{1/2} ]^{1/2} \geqq d$, we could use $\widetilde{P} \geqq D$ to conclude that

7.14 $$\begin{aligned} Z^* T^* \mathbb {H}T Z \geqq \sum _{\alpha \in \mathcal {I}_k} u_\alpha ^2 (k) c_\alpha ^* (k) c_\alpha (k) + \frac{1}{2}{\text {tr}}E = \mathbb {D}_\text{ B } + \frac{1}{2}{\text {tr}}E\;. \end{aligned}$$

This comparison is not surprising in view of the discussion of the spectrum of *E*(*k*) in [1]. There the problem is reduced to a rank-one perturbation of the matrix *D*(*k*); the perturbed eigenvalues are all larger than the corresponding unperturbed eigenvalues. However, *E*(*k*) and *D*(*k*) cannot be simultaneously diagonalized, so we do not have an operator inequality between *E*(*k*) and *D*(*k*). This problem is overcome here noting that *E*(*k*) can be diagonalized by a Bogoliubov transformation which leaves $\mathbb {H}_0 - \mathbb {D}_\text{ B }$ (though not $\mathbb {D}_\text{ B }$ alone) invariant.

Since the *c*- and $c^*$-operators are not exactly bosonic, we can expect (7.13) to hold only approximatively, on states with few excitations of the Fermi ball. To prove that this is indeed the case, we need some estimates on the kernels *K*(*k*) and *L*(*k*). The following bound for *K*(*k*) has already been shown in [6, Lemma 2.5].

### Lemma 7.1

(Bogoliubov kernel) There exists a $C >0$ such that for all $k \in \Gamma ^{\text{ nor }}$ we have

$$\begin{aligned} |K(k)_{\alpha ,\beta }| \leqq C \frac{\hat{V} (k)}{M} \qquad \text {for all}\, \alpha ,\beta \in \mathcal {I}_k.\end{aligned}$$

In particular $$\Vert K (k) \Vert _\text{ HS } \leqq C \hat{V} (k)$$.

The following bounds for the antisymmetric matrix *L*(*k*) are new.

### Lemma 7.2

(Kernel of one-particle transformation) Suppose that the parameters $\delta , M, R$ used to define the patch decomposition in Sect. 5 are such that $M \gg R^2 N^{2\delta }$. Then there exists a $C >0$ such that for all $k \in \Gamma ^{\text{ nor }}$ we have

7.15 $$\begin{aligned} \begin{aligned} \Vert L (k) \Vert _{\text{ HS }}&\leqq C \hat{V} (k) \, . \end{aligned} \end{aligned}$$

### Remark

Since *L*(*k*) is the logarithm of an orthogonal matrix, we always have $\Vert L(k) \Vert _\text{ op }\leqq 2\pi$. From Lemma 7.2, we also have $\Vert L(k) \Vert _\text{ op }\leqq C \hat{V} (k)$, which improves the bound if $\hat{V} (k)$ is small.

### Proof

All matrices depend on *k* but in this proof we do not indicate this dependence explicitly. We split the bound in two parts by

$$\begin{aligned} \begin{aligned} \Vert L\Vert _\text{ HS }&= \Vert \log (O\widetilde{O}) \Vert _{\text{ HS }}\leqq C \Vert O\widetilde{O}-1 \Vert _{\text{ HS }}\leqq C \Vert O \Vert _\text{ op }\Vert \widetilde{O}-1 \Vert _{\text{ HS }}+ C \Vert O - 1 \Vert _{{\text{ HS }}}. \end{aligned} \end{aligned}$$

Since *O* is orthogonal we have $\Vert O \Vert _\text{ op }=1$ and we only need to estimate $\Vert \widetilde{O}-1 \Vert _{\text{ HS }}$ and $\Vert O - 1 \Vert _{\text{ HS }}$. The same applies for the operator norm.

**Bound for** $\Vert \widetilde{O}-1 \Vert _{\text{ HS }}$. From the Definition (7.9), we get

7.16 $$\begin{aligned} \Vert \widetilde{O} - 1 \Vert _{\text{ HS }}= \Vert A - 1 \Vert _{\text{ HS }}\end{aligned}$$

with *A* the orthogonal matrix arising from the polar decomposition of $X = (d+2b)^{1/2} d^{1/2}$, that is, $A = X (X^* X)^{-1/2}$. We have

7.17 $$\begin{aligned} \Vert A - 1 \Vert _{\text{ HS }}&= \left\| X \frac{1}{\sqrt{X^* X}} - 1 \right\| _{\text{ HS }}\leqq \left\| X \left( \frac{1}{\sqrt{X^* X}} - \frac{1}{d} \right) \right\| _{\text{ HS }}+ \left\| X \frac{1}{d} - 1 \right\| _{\text{ HS }}\;.\nonumber \\ \end{aligned}$$

To bound the second term on the right-hand side of the last equation, we use the representation

7.18 $$\begin{aligned} \sqrt{z} = \frac{1}{\pi } \int _0^\infty \frac{{\text{ d }}s}{\sqrt{s}} \left( 1 - \frac{s}{s+z} \right) \end{aligned}$$

to write by means of a resolvent identity

7.19 $$\begin{aligned} \begin{aligned} X \frac{1}{d} - 1&= \left( (d+2b)^{1/2} - d^{1/2} \right) \frac{1}{d^{1/2}}\\ {}&= -\frac{1}{\pi } \int _0^\infty {\text{ d }}s \, \sqrt{s} \, \left( \frac{1}{s+d+2b} - \frac{1}{s+d} \right) \frac{1}{d^{1/2}} \\ {}&= \frac{2}{\pi } \int _0^\infty {\text{ d }}s \sqrt{s}\, \frac{1}{s+d+2b} \, b \, \frac{1}{s+d} \, \frac{1}{d^{1/2}} \;.\end{aligned} \end{aligned}$$

Recalling that $b = g |v \rangle \langle v |$ with $g = \kappa \hat{V} (k) /2$ we find that

7.20 $$\begin{aligned} \big \Vert X \frac{1}{d} - 1 \big \Vert _\text {HS} \leqq C \hat{V} (k) \int _0^\infty {\text{ d }}s \, \sqrt{s} \, \Big \Vert \frac{1}{s+d+2b} v \Big \Vert \, \Big \Vert \frac{1}{s+d} \frac{1}{d^{1/2}} v \Big \Vert \;. \end{aligned}$$

To control the norms in this integral (and similar norms that will arise in the rest of the proof), we use (7.6) so that, for $j=1,2$ and $-1/2 \leqq k \leqq j-1$, we have

7.21 $$\begin{aligned} \Big \Vert \frac{1}{s+d^j} d^k v \Big \Vert ^2 = \Big \langle v , \frac{d^{2k}}{(s+d^j)^2} v \Big \rangle = \sum _{\alpha \in \mathcal {I}_{k}^{+}} \frac{v_\alpha ^2 u_\alpha ^{4k}}{(s+u_\alpha ^{2j})^2} \leqq \frac{C}{M} \sum _\alpha \frac{u_\alpha ^{4k+2}}{(s+u_\alpha ^{2j})^2} \;. \end{aligned}$$

Recall that $u_\alpha ^2 = |\hat{k} \cdot \hat{\omega }_\alpha | = \cos \theta _\alpha$ where $\theta _\alpha \in (0;\pi /2)$ is the inclination angle of the center $\omega _\alpha$ of the patch $B_\alpha$, measured with respect to the vector *k*. We consider then the sum on the right-hand side of (7.21) as a Riemann sum for a surface integral on the northern hemisphere of the unit sphere, parametrized by the angles $\theta \in (0,\pi /2)$ and $\varphi \in (0,2\pi )$. To estimate the error in going from the Riemann sum to the integral, we set

$$\begin{aligned} f(\theta ) = \frac{\cos ^{2k+1} \theta }{(s+\cos ^j \theta )^2} \end{aligned}$$

and compute its derivative, finding that

$$\begin{aligned} f' (\theta ) = f(\theta ) \left( (2k+1) \frac{\sin \theta }{\cos \theta } - 2j \frac{\cos ^{j-1} \theta \sin \theta }{(s+\cos ^j \theta )} \right) \;. \end{aligned}$$

Let $p_\alpha$ denote the surface area on the unit sphere $\mathbb {S}_2$ covered by the patch $B_\alpha$. With slight abuse of notation, let us also write $p_\alpha$ for the set of inclination angles $\theta \in (0,\pi /2)$ corresponding to points in $p_\alpha$. For all $\theta , {\tilde{\theta }} \in p_\alpha$ we have $|\theta - {\tilde{\theta }}| \leqq C M^{-1/2}$ (this being the order of the diameter of the patch). According to the Definition (5.1) of the index set, for $\alpha \in \mathcal {I}_{k}^{+}$ we have $\cos \theta _\alpha \geqq R^{-1} N^{-\delta }$. Thus for all $\theta \in p_\alpha$ we have

$$\begin{aligned} \cos \theta \geqq \cos \theta _\alpha - |\cos \theta - \cos \theta _\alpha |\geqq R^{-1} N^{-\delta } - CM^{-1/2} \geqq \frac{1}{2} R^{-1} N^{-\delta }\,, \end{aligned}$$

where we recall the assumption $M \gg R^2 N^{2\delta }$. Moreover, by the mean value theorem (if necessary enlarging the set of angles $p_\alpha$ to its convex hull in all the following supremuma to make sure that $\theta _0$ is contained)

$$\begin{aligned} | f(\theta ) - f({\tilde{\theta }})| \leqq \sup _{\theta _0 \in p_\alpha } |f'(\theta _0)||\theta - {\tilde{\theta }} |\leqq C \frac{R N^\delta }{\sqrt{M}} \sup _{\theta _0 \in p_\alpha } f(\theta _0) \;. \end{aligned}$$

This implies $| f(\theta ) - f({\tilde{\theta }}_\alpha )| \leqq 2^{-1} \sup _{\theta _0 \in p_\alpha } f(\theta _0)$. Thus for all $\theta \in p_\alpha$ we have

$$\begin{aligned}\sup _{{\tilde{\theta }} \in p_\alpha } f({\tilde{\theta }}) \leqq \sup _{{\tilde{\theta }} \in p_\alpha } |f({\tilde{\theta }}) - f(\theta ) | + f(\theta ) \leqq \frac{1}{2} \sup _{{\tilde{\theta }} \in p_\alpha } f({\tilde{\theta }}) + f(\theta )\;;\end{aligned}$$

in particular $f(\theta _\alpha ) \leqq 2 f(\theta )$ for all $\theta \in p_\alpha$. Therefore

$$\begin{aligned} \Big \Vert \frac{1}{s+d^j} d^k v \Big \Vert ^2 \leqq C \sum _{\alpha \in \mathcal {I}_{k}^{+}} \int _{p_\alpha } \frac{ \cos ^{2k+1} \theta }{(s+\cos ^j \theta )^2} \sin \theta {\text{ d }}\theta {\text{ d }}\varphi \leqq C \int _0^1 \frac{t^{2k+1}}{(s+t^j)^2} {\text{ d }}t \;. \end{aligned}$$

We conclude that

7.22 $$\begin{aligned} \Big \Vert \frac{1}{s+d^j} d^k v \Big \Vert \leqq C \left\{ \begin{array}{ll} \min \{ s^{-1} , s^{- 1+ (1+k)/j} \} \; &{}\text {if } \; 1+k < j \\ \min \{ s^{-1} , |\log s|^{1/2} \} \; &{}\text {if } \; 1+k = j \;. \end{array} \right. \end{aligned}$$

In particular, with $j=1$, $k=-1/2$, we find that

$$\begin{aligned} \begin{aligned} \Big \Vert \frac{1}{s+d} \frac{1}{d^{1/2}} v \Big \Vert \leqq C \min \{ s^{-1} ; s^{-1/2} \} \;. \end{aligned} \end{aligned}$$

To bound the other norm in the integral in (7.20), we write that

$$\begin{aligned} \frac{1}{s+d+2b} v&= \frac{1}{s+d} v - 2 \frac{1}{s+d+2b} b \frac{1}{s+d} v \\&= \frac{1}{s+d} v - 2\Big \langle v, \frac{1}{s+d} v \Big \rangle \, \frac{1}{s+d+2b} v, \end{aligned}$$

which implies, applying (7.22) with $j=1$ and $k=0$, that

$$\begin{aligned} \Big \Vert \frac{1}{s+d+2b} v \Big \Vert \leqq \Big \Vert \frac{1}{s+d} v \Big \Vert \leqq C \min \{ s^{-1}, |\log s|^{1/2} \} \;. \end{aligned}$$

Inserting this bound in (7.20) and integrating the variable *s* separately over the intervals [0, 1] and $[1,\infty )$, we conclude that

$$\begin{aligned} \big \Vert X \frac{1}{d} - 1 \big \Vert _\text {HS} \leqq C \hat{V} (k) \;. \end{aligned}$$

As for the first term on the right-hand side of (7.17), we proceed analogously, writing

$$\begin{aligned} \begin{aligned}&X \left( \frac{1}{\sqrt{X^* X}} - \frac{1}{d} \right) = \frac{1}{\pi } \int _0^\infty \frac{{\text{ d }}s}{\sqrt{s}} \, X \left( \frac{1}{s+d^{1/2} (d+2b) d^{1/2}} - \frac{1}{s+d^2} \right) \\&\quad = -\frac{2}{\pi } \int _0^\infty \frac{{\text{ d }}s}{\sqrt{s}} \, (d+2b)^{1/2} d^{1/2} \, \frac{1}{s+d^{1/2} (d+2b) d^{1/2}} d^{1/2} \, b \, d^{1/2} \frac{1}{s+d^2} \;. \end{aligned} \end{aligned}$$

We write $b = g |v \rangle \langle v |$. We can bound $\Vert d^{-1/2} v \Vert \leqq C$, as well as

$$\begin{aligned} \begin{aligned} \left\| (d+2b)^{1/2} d^{1/2} \, \frac{1}{s+d^{1/2} (d+2b) d^{1/2}} \, d^{1/2} (d+2b)^{1/2} \right\| _\text{ op }&\leqq 1 \;,\\ \Vert (d+2b)^{-1/2} d^{1/2} \Vert _\text{ op }&\leqq 1 \;, \end{aligned} \end{aligned}$$

and, using (7.22) with $j=2$ and $k=1/2$,

$$\begin{aligned} \Big \Vert \frac{1}{s+d^2} d^{1/2} v \Big \Vert \leqq C \min \{ s^{-1} , s^{-1/4} \} \;. \end{aligned}$$

We conclude that

$$\begin{aligned} \left\| X \left( \frac{1}{\sqrt{X^* X}} - \frac{1}{d} \right) \right\| _{\text{ HS }}\leqq C \hat{V} (k) \, . \end{aligned}$$

Combined with (7.17) and (7.20), this implies that

$$\begin{aligned} \Vert A - 1 \Vert _{\text{ HS }}\leqq C \hat{V} (k) \;. \end{aligned}$$

**Bound for** $\Vert O-1\Vert _{\text{ HS }}$. Recall that *O* arises from the polar decomposition (7.2) of $S_1$, that is,

$$\begin{aligned} O = S_1 |S_1|^{-1} = (D+W-\widetilde{W})^{1/2} E^{-1/2} \frac{1}{\sqrt{E^{-1/2} (D+W-\widetilde{W}) E^{-1/2}}}\;. \end{aligned}$$

Using the orthogonal matrix *U* defined in (7.7) and the fact that $O-1$ and $U^T (O-1) U$ have the same spectrum we obtain

7.23 $$\begin{aligned} \begin{aligned} \Vert O-1 \Vert _{\text{ HS }}\leqq \;&\Big \Vert d^{1/2} (X^* X)^{-1/4} \frac{1}{\sqrt{(X^* X)^{-1/4} d (X^* X)^{-1/4}}} -1 \Big \Vert _{\text{ HS }}\\&+ \Big \Vert (d+2b)^{1/2} (X X^*)^{-1/4} \frac{1}{\sqrt{(X X^*)^{-1/4} (d+2b) (X X^*)^{-1/4}}} -1 \Big \Vert _{\text{ HS }}\;. \end{aligned} \end{aligned}$$

To estimate the first norm on the right-hand side of (7.23) we decompose

7.24 $$\begin{aligned} \begin{aligned}&d^{1/2} (X^* X)^{-1/4} \frac{1}{\sqrt{(X^* X)^{-1/4} d (X^* X)^{-1/4}}} -1 \\&\quad = d^{1/2} \left( (X^* X)^{-1/4} - d^{-1/2} \right) \frac{1}{\sqrt{(X^* X)^{-1/4} d (X^* X)^{-1/4}}} \\&\qquad + \frac{1}{\sqrt{(X^* X)^{-1/4} d (X^* X)^{-1/4}}} -1 \;. \end{aligned} \end{aligned}$$

We start with the first summand on the right-hand side of (7.24). With an integral representation similar to (7.18) and using $X^* X - d^2 = 2d^{1/2} b d^{1/2}$, we write it as

7.25 $$\begin{aligned} \begin{aligned}&d^{1/2} \left( (X^* X)^{-1/4} - d^{-1/2} \right) \frac{1}{\sqrt{(X^* X)^{-1/4} d (X^* X)^{-1/4}}} \\&\quad = C \int _0^\infty \frac{{\text{ d }}s}{s^{1/4}} \, d^{1/2} \frac{1}{s+d^2} \, d^{1/2} \, b \, d^{1/2} \frac{1}{s+ X^* X} \frac{1}{\sqrt{(X^* X)^{-1/4} d (X^* X)^{-1/4}}}\;. \end{aligned} \end{aligned}$$

We estimate $\Vert d^{-1/2} v \Vert \leqq C$ and

$$\begin{aligned} \begin{aligned}&\Big \Vert d \frac{1}{s+X^* X} \frac{1}{\sqrt{(X^* X)^{-1/4} d (X^* X)^{-1/4}}} \Big \Vert _\text{ op}^2 \\&\quad \leqq \Big \Vert d \frac{1}{s+X^* X} \frac{1}{(X^*X)^{-1/4} d (X^*X)^{-1/4}} \frac{1}{s+X^* X} \, d \, \Big \Vert _\text{ op }\\&\quad \leqq \Vert d (X^* X)^{-1/2} \Vert _\text{ op }\Big \Vert \frac{(X^* X)^{1/4}}{s+X^* X} \Big \Vert _\text{ op }\Vert (X^* X)^{1/2} d^{-1} \Vert _\text{ op }\Big \Vert \frac{(X^* X)^{3/4}}{s+X^* X} \Big \Vert _\text{ op }\Vert (X^* X)^{-1/2} d \Vert _\text{ op }\\ {}&\leqq C \min \{ s^{-2} ,s^{-1} \} \;. \end{aligned} \end{aligned}$$

Here we used (recalling $X^* X = d^{1/2} (d+2b) d^{1/2}$) that $\Vert d (X^* X)^{-1/2} \Vert _\text{ op }\leqq 1$ and also

7.26 $$\begin{aligned} \Vert (X^* X)^{1/2} d^{-1} \Vert _\text{ op}^2 = \Vert 1 + d^{-1/2} b d^{-1/2} \Vert _\text{ op }\leqq C\;. \end{aligned}$$

Using (7.22) with $j=2$, $k=1$, we obtain

$$\begin{aligned} \Big \Vert \frac{1}{s+d^2} d v \Big \Vert \leqq C \min \{ s^{-1} , |\log s |^{1/2} \} \;. \end{aligned}$$

We conclude therefore that

7.27 $$\begin{aligned} \Big \Vert d^{1/2} \left( (X^* X)^{-1/4} - d^{-1/2} \right) \frac{1}{\sqrt{(X^* X)^{-1/4} d (X^* X)^{-1/4}}} \Big \Vert _{\text{ HS }}\leqq C\hat{V} (k)\;. \end{aligned}$$

Let us now consider the second summand on the right-hand side of (7.24). Since $X^* X = d^{1/2} (d+2b) d^{1/2} \geqq d^2$, we observe that

$$\begin{aligned} d^{1/2} (X^*X)^{-1/2} d^{1/2} \leqq 1\;. \end{aligned}$$

From $d^{-1/2} b d^{-1/2} \leqq C$ (uniformly in *N* and in *k*, since $\hat{V}$ is bounded), we also have $X^* X \leqq C d^2$ and thus

$$\begin{aligned} d^{1/2} (X^*X)^{-1/2} d^{1/2} \geqq c \end{aligned}$$

for a constant $c > 0$, independent of *N* and *k*. The last two bounds imply that $c \leqq (X^* X)^{-1/4} d (X^* X)^{-1/4} \leqq 1$ and therefore that with

$$\begin{aligned} J := 1 - (X^* X)^{-1/4} d (X^* X)^{-1/4} \end{aligned}$$

we have

$$\begin{aligned} 0 \leqq J \leqq 1-c < 1\;.\end{aligned}$$

We write

$$\begin{aligned} \frac{1}{\sqrt{(X^* X)^{-1/4} d (X^* X)^{-1/4}}} -1 = \frac{1}{\sqrt{1 - J}} - 1 = \frac{1}{\pi } \int _0^\infty \frac{{\text{ d }}s}{\sqrt{s}} \frac{1}{s + 1 - J} J \frac{1}{s+1} \;. \end{aligned}$$

With $1-J \geqq c > 0$, we conclude that

7.28 $$\begin{aligned} \Big \Vert \frac{1}{\sqrt{(X^* X)^{-1/4} d (X^* X)^{-1/4}}} -1 \Big \Vert _{\text{ HS }}\leqq C \Vert J \Vert _{\text{ HS }}\;. \end{aligned}$$

To estimate the Hilbert–Schmidt norm of *J*, we expand, similarly as we did in (7.19),

$$\begin{aligned} \begin{aligned} J&= (X^* X)^{-1/4} ((X^*X)^{1/2} - d) (X^* X)^{-1/4} \\ {}&= \frac{1}{\pi } \int _0^\infty {\text{ d }}s \, \sqrt{s} \, (X^* X)^{-1/4} \frac{1}{s+X^*X} \, d^{1/2} \, b \, d^{1/2} \, \frac{1}{s+d^2}\, (X^*X)^{-1/4} \;. \end{aligned} \end{aligned}$$

Writing again $b = g |v \rangle \langle v |$ and using the bounds $\Vert d^{-1/2} v \Vert \leqq C$, $\Vert (X^*X)^{-1/4} d^{1/2} \Vert _\text{ op }\leqq C$, and $\Vert d (X^*X)^{-1/2} \Vert _\text{ op }\leqq C$ (the latter two bounds are simple consequences of $X^* X \geqq d^2$),

$$\begin{aligned} \begin{aligned} \Vert (X^*X)^{1/4} (s+ X^*X)^{-1} \Vert _\text{ op }&\leqq \min \big \{ s^{-3/4} , s^{-1} \big \} \end{aligned} \end{aligned}$$

and also (7.22) with $j=2$, $k=0$ to bound

$$\begin{aligned} \Big \Vert \frac{1}{s+d^2} v \Big \Vert \leqq \min \{ s^{-1}, s^{-1/2} \} \, , \end{aligned}$$

we arrive at $\Vert J \Vert _{\text{ HS }}\leqq C \hat{V} (k)$. Inserting in (7.28) and combining the resulting bound with (7.27), we conclude that

7.29 $$\begin{aligned} \Big \Vert d^{1/2} (X^* X)^{-1/4} \frac{1}{\sqrt{(X^* X)^{-1/4} d (X^* X)^{-1/4}}} -1 \Big \Vert _{\text{ HS }}\leqq C \hat{V} (k) \;. \end{aligned}$$

We turn to the second term on the right-hand side of (7.23). Similarly as for the first term

7.30 $$\begin{aligned}&(d+2b)^{1/2} (X X^*)^{-1/4} \frac{1}{\sqrt{(X X^*)^{-1/4} (d+2b) (X X^*)^{-1/4}}} -1 \nonumber \\&\quad = (d+2b)^{1/2} \left( (X X^*)^{-1/4} - (d+2b)^{-1/2} \right) \frac{1}{\sqrt{(X X^*)^{-1/4} (d+2b) (X X^*)^{-1/4}}} \nonumber \\&\qquad + \frac{1}{\sqrt{(X X^*)^{-1/4} (d+2b) (X X^*)^{-1/4}}} -1\;. \end{aligned}$$

The term on the first line can be bounded analogously as we did with the first term on the right-hand side of (7.24). With $XX^* - (d+2b)^2 = - 2 (d+2b)^{1/2} \, b \, (d+2b)^{1/2}$ we find that

7.31 $$\begin{aligned} \begin{aligned}&(d+2b)^{1/2} \left( (X X^*)^{-1/4} - (d+2b)^{-1/2} \right) \frac{1}{\sqrt{(X X^*)^{-1/4} (d+2b) (X X^*)^{-1/4}}} \\&\quad = C \int _0^\infty \frac{{\text{ d }}s}{s^{1/4}} \, (d+2b)^{1/2} \, \frac{1}{s+ (d+2b)^2} (d+2b)^{1/2} \, b \, (d+2b)^{1/2} \, \frac{1}{s+XX^*} \\&\qquad \times \frac{1}{\sqrt{(X X^*)^{-1/4} (d+2b) (X X^*)^{-1/4}}} \;. \end{aligned} \end{aligned}$$

From $\Vert d^{1/2} (d+2b)^{-1/2} \Vert _\text{ op }\leqq C$ and $\Vert d^{-1/2} v \Vert \leqq C$, we obtain $\Vert (d+2b)^{-1/2} v \Vert \leqq C$. Moreover, we find that

$$\begin{aligned} \begin{aligned}&\Big \Vert (d+2b) \frac{1}{s+XX^*} \frac{1}{\sqrt{(X X^*)^{-1/4} (d+2b) (X X^*)^{-1/4}}} \Big \Vert _\text{ op}^2 \\ {}&\quad = \Big \Vert (d+2b) \frac{1}{s+XX^*} \frac{1}{(X X^*)^{-1/4} (d+2b) (X X^*)^{-1/4}} \frac{1}{s+XX^*} (d+2b) \Big \Vert _\text{ op }\\&\quad \leqq \Vert (d+2b) (XX^*)^{-1/2} \Vert _\text{ op }\Big \Vert \frac{(XX^*)^{1/4}}{s + XX^*} \Big \Vert _\text{ op }\Vert (XX^*)^{1/2} (d+2b)^{-1} \Vert _\text{ op }\\ {}&\qquad \times \Big \Vert \frac{(XX)^{3/4}}{s + XX^*} \Big \Vert _\text{ op }\Vert (XX^*)^{-1/2} (d+2b) \Vert _\text{ op }\\&\quad \leqq C \min \big \{ s^{-2}, s^{-1} \big \} \;. \end{aligned} \end{aligned}$$

Here we used, analogously to (7.26), the bounds $\Vert (XX^*)^{1/2} (d+2b)^{-1} \Vert _\text{ op }\leqq 1$ and

7.32 $$\begin{aligned} \begin{aligned}&\Vert (d+2b) (XX^*)^{-1/2} \Vert ^2_\text{ op }\\&\quad = \Vert (d+2b) (XX^*)^{-1} (d+2b) \Vert _\text{ op }\\&\quad = \Vert (d+2b)^{1/2} d^{-1} (d+2b)^{1/2} \Vert _\text{ op }\\ {}&= \Vert d^{-1/2} (d+2b) d^{-1/2} \Vert _\text{ op }= \Vert 1 + 2 d^{-1/2} b d^{-1/2} \Vert _\text{ op }\leqq C \; . \end{aligned} \end{aligned}$$

On the other hand, we can bound

$$\begin{aligned} \Big \Vert \frac{1}{s+(d+2b)^2} (d+2b) v \Big \Vert ^2 \leqq \left\langle v, \frac{1}{s+(d+2b)^2 } v \right\rangle \;. \end{aligned}$$

With

$$\begin{aligned} \frac{1}{s+(d+2b)^2 } = \frac{1}{s+d^2} - \frac{1}{s+(d+2b)^2} \left[ (d+ 2b) 2b + 2b d \right] \frac{1}{s+d^2} \end{aligned}$$

and using again $b = g |v \rangle \langle v |$, we get

$$\begin{aligned} \begin{aligned} \Big \langle v, \frac{1}{s+(d+2b)^2 } v \Big \rangle =\;&\big \langle v, \frac{1}{s+d^2} v \big \rangle - 2g \big \langle v , \frac{(d+2b)}{s+(d+2b)^2} v \big \rangle \big \langle v, \frac{1}{s+d^2} v \big \rangle \\ {}&- 2g \big \langle v , \frac{1}{s+(d+2b)^2} v \big \rangle \big \langle v, \frac{d}{s+d^2} v \big \rangle \end{aligned} \end{aligned}$$

and therefore (proceeding as in the proof of (7.22)) arrive at

7.33 $$\begin{aligned} \Big \langle v, \frac{1}{s+(d+2b)^2 } v \Big \rangle \leqq \big \langle v, \frac{1}{s+d^2} v \big \rangle \leqq \frac{C}{M} \sum _\alpha \frac{u_\alpha ^2}{s+u_\alpha ^4} \leqq C \min \{ s^{-1} , |\log s| \} \, . \end{aligned}$$

This implies that

7.34 $$\begin{aligned} \Big \Vert \frac{1}{s+(d+2b)^2} (d+2b) v \Big \Vert \leqq C \min \{ s^{-1/2} , |\log s|^{1/2} \} \;.\end{aligned}$$

From (7.31), we conclude that

7.35 $$\begin{aligned} \begin{aligned}&\Big \Vert (d+2b)^{1/2} \left( (X X^*)^{-1/4} - (d+2b)^{-1/2} \right) \frac{1}{\sqrt{(X X^*)^{-1/4} (d+2b) (X X^*)^{-1/4}}} \Big \Vert _{\text{ HS }}\\&\quad \leqq C \hat{V} (k) \, . \end{aligned} \end{aligned}$$

Finally, let us consider the term on the second line of the right-hand side of (7.30). Since $XX^* \leqq (d+2b)^2$ (recall that $XX^* = (d+2b)^{1/2} d (d+2b)^{1/2}$), we have

7.36 $$\begin{aligned} (d+2b)^{1/2} (XX^*)^{-1/2} (d+2b)^{1/2} \geqq 1 \end{aligned}$$

which also implies that $(XX^*)^{-1/4} (d+2b) (XX^*)^{-1/4} \geqq 1$. We define therefore

$$\begin{aligned} W := (XX^*)^{-1/4} (d+2b) (XX^*)^{-1/4} - 1 \geqq 0 \;.\end{aligned}$$

Then we have

$$\begin{aligned}{} & {} \frac{1}{\sqrt{(XX^*)^{-1/4} (d+2b) (XX^*)^{-1/4}}}- 1 = \frac{1}{\sqrt{1+W}} - 1 \\{} & {} \quad = - \frac{1}{\pi } \int _0^\infty \frac{{\text{ d }}s}{\sqrt{s}} \frac{1}{s+1+W} W \frac{1}{s+1} \end{aligned}$$

and thus

7.37 $$\begin{aligned} \Big \Vert \frac{1}{\sqrt{(XX^*)^{-1/4} (d+2b) (XX^*)^{-1/4}}} - 1 \Big \Vert _{\text{ HS }}\leqq C \Vert W \Vert _{\text{ HS }}\;.\end{aligned}$$

To estimate the Hilbert-Schmidt norm of *W* we write that

$$\begin{aligned} W&= (XX^*)^{-1/4} [(d+2b) - (XX^*)^{1/2}] (XX^*)^{-1/4} \\&= \frac{1}{\pi } \int _0^\infty {\text{ d }}s \sqrt{s} \, (XX^*)^{-1/4} \frac{1}{s+(d+2b)^2} \, (d+2b)^{1/2} \, b \, (d+2b)^{1/2} \, \frac{1}{s+XX^*} (XX^*)^{-1/4} \\&= \frac{1}{\pi } \int _0^\infty {\text{ d }}s \, \sqrt{s} \, (XX^*)^{-1/4} (d+2b)^{1/2} \, \frac{(d+2b)^{1/2}}{s+(d+2b)^2} \, (d+2b)^{-1/2} \, b \, (d+2b)^{-1/2} \\&\quad \times (d+2b) \, (XX^*)^{-1/2} \, \frac{(XX^*)^{1/4}}{s+XX^*} \;. \end{aligned}$$

With the resolvent identity, we obtain

$$\begin{aligned}{} & {} \left( 1+ \big \langle v, \frac{d}{s+d^2} v \big \rangle \right) \frac{1}{s + (d+2b)^2} v = \frac{1}{s+d^2} v - \big \langle v, \frac{1}{s+d^2} v \big \rangle \frac{(d+2b)}{s+(d+2b)^2} v \end{aligned}$$

and thus

$$\begin{aligned} \Big \Vert \frac{1}{s + (d+2b)^2} v \Big \Vert \leqq \Big \Vert \frac{1}{s+d^2} v \Big \Vert + \big \langle v, \frac{1}{s+d^2} v \big \rangle \Big \Vert \frac{(d+2b)}{s+(d+2b)^2} v \Big \Vert \;. \end{aligned}$$

Using (7.22) with $j=2$, $k=0$, (7.33), and (7.34) we arrive at

$$\begin{aligned} \Big \Vert \frac{1}{s + (d+2b)^2} v \Big \Vert \leqq C \min \{ s^{-1/2} , s^{-1} \} \;. \end{aligned}$$

Applying also (7.32), $\Vert (d+2b)^{-1/2} v \Vert \leqq C$ and

$$\begin{aligned} \Big \Vert (XX^*)^{1/4} \frac{1}{s+XX^*} \Big \Vert _\text{ op }\leqq C \min \{ s^{-1} , s^{-3/4} \} \end{aligned}$$

we conclude that

7.38 $$\begin{aligned} \Vert W \Vert _{\text{ HS }}\leqq C \Vert (XX^*)^{-1/4} (d+2b)^{1/2} \Vert _\text{ op }\hat{V} (k) \;. \end{aligned}$$

Since

$$\begin{aligned} \begin{aligned} \Vert (XX^*)^{-1/4} (d+2b)^{1/2} \Vert _\text{ op}^2&= \Vert (XX^*)^{-1/4} (d+2b) (XX^*)^{-1/4} \Vert _\text{ op }\\&= \Vert 1 + W \Vert _\text{ op }\leqq 1 + \Vert W \Vert _{\text{ HS }}\end{aligned} \end{aligned}$$

we arrive at

$$\begin{aligned} \Vert W \Vert _{\text{ HS }}\leqq C \hat{V} (k) \;. \end{aligned}$$

Inserting this bound in (7.37) and combining it with (7.35), we can bound (7.30) by

$$\begin{aligned} \Big \Vert (d+2b)^{1/2} (X X^*)^{-1/4} \frac{1}{\sqrt{(X X^*)^{-1/4} (d+2b) (X X^*)^{-1/4}}} -1 \Big \Vert _{\text{ HS }}\leqq C \hat{V} (k)\,.\end{aligned}$$

Together with (7.29) and with (7.23), we obtain

$$\begin{aligned} \Vert O-1 \Vert _{\text{ HS }}\leqq C \hat{V} (k) \;. \end{aligned}$$

$$\square $$

Using the bounds on the kernels *K*(*k*) and *L*(*k*), our next goal is to show that the unitary transformations *T* and *Z* defined in (7.11) act on the *c*- and $c^*$-operators as bosonic Bogoliubov transformations, up to errors that are small on states with few excitations. (This will allow us to show that conjugation of the right-hand side of (6.4) by *T* and *Z* produces approximately the right-hand side of (7.13).) To reach this goal, we need to show first that conjugation with *T* and *Z* does not change the number operator $\mathcal {N}$ and the gapped number operators $\mathcal {N}_\delta$ substantially. We generalize the Definition (7.11) for $\lambda \in \mathbb {R}$ to

7.39 $$\begin{aligned} \begin{aligned} T_\lambda&:= \exp \left( \frac{\lambda }{2} \sum _{k \in \Gamma ^{\text{ nor }}} \sum _{\alpha ,\beta \in \mathcal {I}_k} K (k)_{\alpha ,\beta } c_\alpha ^* (k) c_\beta ^* (k) - \text {h.c.} \right) , \\ Z_\lambda&:= \exp \left( \lambda \sum _{k \in \Gamma ^{\text{ nor }}} \sum _{\alpha ,\beta \in \mathcal {I}_k} L (k)_{\alpha ,\beta } c_\alpha ^* (k) c_\beta (k) \right) \;, \end{aligned} \end{aligned}$$

so that $T = T_1$ and $Z = Z_1$.

### Lemma 7.3

(Stability of number operators) Assume $\Vert \hat{V} \Vert _1 < \infty$ and $M \gg N^{2\delta } R^2$. Then for every $m \in \mathbb {N}$ there exists $C > 0$ such that for all $\lambda \in [-1,1]$ we have

7.40 $$\begin{aligned} \begin{aligned} T_\lambda ^* \mathcal {N}^m T_\lambda \leqq C (\mathcal {N}+1)^m \quad \text {and} \quad T_\lambda ^* \mathcal {N}_\delta \mathcal {N}^m T_\lambda&\leqq C (\mathcal {N}_\delta + 1)( \mathcal {N}+1)^m \;. \end{aligned} \end{aligned}$$

Conjugation with $Z_\lambda$ leaves the total number of particles constant,

$$\begin{aligned} Z_\lambda ^* \mathcal {N}^m Z_\lambda = \mathcal {N}^m\;. \end{aligned}$$

Moreover, for every $m \in \mathbb {N}$ there exists $C > 0$ such that, for all $\lambda \in [-1,1]$, we have

7.41 $$\begin{aligned} \begin{aligned} Z_\lambda ^* \mathcal {N}_\delta \mathcal {N}^m Z_\lambda&\leqq C \mathcal {N}_\delta \mathcal {N}^m \;. \end{aligned} \end{aligned}$$

### Proof

The proof of (7.40) can be found in [5, Lemma 7.2] where it is stated under the additional assumption that $\hat{V}$ has a compact support; however, using Lemma 7.1 it easily extends to $\Vert \hat{V} \Vert _1 < \infty$.

The invariance of $\mathcal {N}$ with respect to $Z_\lambda$ follows since the exponent commutes with $\mathcal {N}$ (the $c^*$-operator creates two fermions while the *c*-operator annihilates two fermions).

We still have to show (7.41). We consider the case $m=0$; the extension to $m > 0$ is straightforward. We compute that

7.42 $$\begin{aligned} \frac{{\text{ d }}}{{\text{ d }}\lambda } \langle \psi , Z_\lambda ^* \mathcal {N}_\delta Z_\lambda \psi \rangle = \sum _{k \in \Gamma ^{\text{ nor }}} \sum _{\alpha , \beta \in \mathcal {I}_k} L_{\alpha ,\beta } (k) \langle \psi , Z_\lambda ^* \left[ c_\alpha ^* (k) c_\beta (k) , \mathcal {N}_\delta \right] Z_\lambda \psi \rangle \;. \end{aligned}$$

Using the weighted pairs operators introduced in Lemma 5.3 we have

$$\begin{aligned} {[}c_\alpha ^* (k) , \mathcal {N}_\delta ] = c_\alpha ^{g*} (k)\; , \qquad [ c_\beta (k) , \mathcal {N}_\delta ] = - c_\beta ^g (k) \end{aligned}$$

for a weight function *g* with values in $\{ 0,1,2 \}$. Thus

$$\begin{aligned} \frac{{\text{ d }}}{{\text{ d }}\lambda } \langle \psi , Z_\lambda ^* \mathcal {N}_\delta Z_\lambda \psi \rangle = \sum _{k \in \Gamma ^{\text{ nor }}} \sum _{\alpha , \beta \in \mathcal {I}_k} L_{\alpha ,\beta } (k) \left\langle \psi , Z_\lambda ^* \left[ c_\alpha ^{g*} (k) c_\beta (k) + c_\alpha ^* (k) c_\beta ^g (k) \right] Z_\lambda \psi \right\rangle , \end{aligned}$$

and by Cauchy–Schwarz,

$$\begin{aligned} \left| \frac{{\text{ d }}}{{\text{ d }}\lambda } \langle \psi , Z_\lambda ^* \mathcal {N}_\delta Z_\lambda \psi \rangle \right| \leqq \sum _{k \in \Gamma ^{\text{ nor }}} \left( \sum _{\beta \in \mathcal {I}_k}\Big \Vert \sum _{\alpha \in \mathcal {I}_k} L_{\alpha , \beta } (k) \, c_\alpha ^g (k) Z_\lambda \psi \Big \Vert ^2 \right) ^{\frac{1}{2}} \left( \sum _{\beta \in \mathcal {I}_k} \Vert c_\beta (k) Z_\lambda \psi \Vert ^2 \right) ^{\frac{1}{2}} \;. \end{aligned}$$

Observe that

$$\begin{aligned} \begin{aligned}&\sum _{\beta \in \mathcal {I}_k} \Big \Vert \sum _{\alpha \in \mathcal {I}_k} L_{\alpha , \beta } (k) c_\alpha ^g (k) Z_\lambda \psi \Big \Vert ^2 \\ {}&\quad = \sum _{\beta , \alpha , \alpha ' \in \mathcal {I}_k} L_{\alpha ,\beta } (k) \overline{L_{\alpha ' , \beta }} (k) \, \langle c_\alpha ^g (k) Z_\lambda \psi , c_{\alpha '}^g (k) Z_\lambda \psi \rangle \\&\quad = \sum _{\alpha ,\alpha ' \in \mathcal {I}_k} |L (k)|^2_{\alpha ,\alpha '} \langle c_\alpha ^g (k) Z_\lambda \psi , c_{\alpha '}^g (k) Z_\lambda \psi \rangle = {\text {tr}}\, |L (k)|^2 C_g \end{aligned} \end{aligned}$$

with the $|\mathcal {I}_k| \times |\mathcal {I}_k|$ matrix $C_g$ having entries $(C_g)_{\alpha ,\alpha '} = \langle c_\alpha ^g (k) Z_\lambda \psi , c_{\alpha '}^g (k) Z_\lambda \psi \rangle$. Since $C_g$ is a positive matrix, we can use (7.15) to estimate that

$$\begin{aligned} \begin{aligned} \sum _{\beta \in \mathcal {I}_k} \Big \Vert \sum _{\alpha \in \mathcal {I}_k} L_{\alpha , \beta } (k) c_\alpha ^g (k) Z_\lambda \psi \Big \Vert ^2&\leqq C \hat{V} (k)^2 \, {\text {tr}}C_g = C \hat{V} (k)^2 \sum _{\alpha \in \mathcal {I}_k} \Vert c_\alpha ^g (k) Z_\lambda \psi \Vert ^2\;. \end{aligned} \end{aligned}$$

Applying Lemma 5.3 and using $\Vert \hat{V} \Vert _1 < \infty$, we find that

$$\begin{aligned} \left| \frac{{\text{ d }}}{{\text{ d }}\lambda } \langle \psi , Z_\lambda ^* \mathcal {N}_\delta Z_\lambda \psi \rangle \right| \leqq C \langle \psi , Z_\lambda ^* \mathcal {N}_\delta Z_\lambda \psi \rangle \;. \end{aligned}$$

By Grönwall’s lemma, we conclude that for all $\lambda \in [-1,1]$ we have

$$\begin{aligned} \langle \psi , Z_\lambda ^* \mathcal {N}_\delta Z_\lambda \psi \rangle \leqq C \langle \psi , \mathcal {N}_\delta \psi \rangle \;. \end{aligned}$$

$$\square $$

We can now show that the unitary operators *T* and *Z* approximately act on *c*- and $c^*$-operators as bosonic Bogoliubov transformations, up to errors that are negligible on states with few excitations. The action of *T* is described in the next lemma, whose proof can be found in [5, Lemma 7.1].

### Lemma 7.4

(Approximate bosonic Bogoliubov transformation) For all $\lambda \in [-1,1]$, $k \in \Gamma ^{\text{ nor }}$, and $\gamma \in \mathcal {I}_k$, we have

7.43 $$\begin{aligned} T_\lambda ^* c_\gamma (k) T_\lambda = \sum _{\alpha \in \mathcal {I}_k} \cosh (\lambda K (k))_{\alpha ,\gamma } c_\alpha (k) + \sum _{\alpha \in \mathcal {I}_k} \sinh (\lambda K(k))_{\alpha , \gamma } c_\alpha ^* (k) + \mathfrak {E}_\gamma (\lambda , k) \end{aligned}$$

where for the error term $\mathfrak {E}_\gamma (\lambda , k)$ there exists a $C > 0$ such that for all $\psi \in \mathcal {F}$ we have

$$\begin{aligned} \sum _{\gamma \in \mathcal {I}_k} \Vert \mathfrak {E}_{\gamma } (\lambda , k) \psi \Vert \leqq C M N^{-2/3+\delta } \Vert (\mathcal {N}_\delta + M)^{1/2} (\mathcal {N}+ 1) \psi \Vert \;. \end{aligned}$$

The same bound holds if we replace $\mathfrak {E}_\gamma (\lambda ,k)$ with $\mathfrak {E}_\gamma ^* (\lambda ,k)$.

In the next lemma, we control the action of *Z* in an analogous fashion.

### Lemma 7.5

(Approximate bosonic one-particle unitary) Assume $\Vert \hat{V} \Vert _1 < \infty$. Let $M \gg R^2 N^{2\delta }$. Then for every $\ell \in \Gamma ^{\text{ nor }}$, $\gamma \in \mathcal {I}_\ell$, and $\lambda \in [-1,1]$ we have

7.44 $$\begin{aligned} \begin{aligned} Z_{\lambda }^* c_\gamma (\ell ) Z_{\lambda }&= \sum _{\beta \in \mathcal {I}_\ell } \exp (\lambda L (\ell ))_{\gamma ,\beta } c_\beta (\ell ) + \mathfrak {F}_\gamma (\lambda , \ell ) \end{aligned} \end{aligned}$$

where there exists a $C > 0$ such that for all $\psi \in \mathcal {F}$ we have

7.45 $$\begin{aligned} \begin{aligned} \sum _{\gamma \in \mathcal {I}_\ell } \Vert \mathfrak {F}_\gamma (\lambda , \ell ) \psi \Vert&\leqq C N^{-2/3+ \delta } M^{3/2} \Vert \mathcal {N}_\delta ^{1/2} \mathcal {N}\psi \Vert \;. \end{aligned} \end{aligned}$$

### Proof

Recall that *L* is antisymmetric; hence $Z_{\lambda }^*$ has the same form as $Z_{\lambda }$, but with *L* replaced by $-L$. For $\lambda \in [-1,1]$ we compute that

$$\begin{aligned} \begin{aligned} \frac{{\text{ d }}}{{\text{ d }}\lambda } \, Z_{\lambda }^* c_\gamma (\ell ) Z_{\lambda }&= \sum _{\beta \in \mathcal {I}_\ell } L (\ell )_{\gamma ,\beta } Z_{\lambda }^* c_\beta (\ell ) Z_{\lambda }\\&\quad + \sum _{k \in \Gamma ^{\text{ nor }}: \gamma \in \mathcal {I}_k} \sum _{\beta \in \mathcal {I}_k} L (k)_{\gamma ,\beta } \, Z_{\lambda }^* \mathcal {E}_\gamma (\ell , k) c_\beta (k) Z_{\lambda } \end{aligned} \end{aligned}$$

with the error operator $\mathcal {E}_\gamma (\ell , k)$ introduced in (5.6). In integral form, we obtain

$$\begin{aligned} \begin{aligned} Z_{\lambda }^* c_\gamma (\ell ) Z_{\lambda } = \;&c_\gamma (\ell ) + \sum _{\beta \in \mathcal {I}_\ell } L (\ell )_{\gamma ,\beta } \int _0^\lambda {\text{ d }}\tau \, Z_{\tau }^* c_\beta (\ell ) Z_{\tau } \\ {}&+ \sum _{k \in \Gamma ^{\text{ nor }}: \gamma \in \mathcal {I}_k} \sum _{\beta \in \mathcal {I}_k} L (k)_{\gamma ,\beta } \int _0^\lambda {\text{ d }}\tau \, Z_{\tau }^* \mathcal {E}_\gamma (\ell , k) c_\beta (k) Z_{\tau }\;. \end{aligned} \end{aligned}$$

Iterating $n_0$ times, we find (with $L (\ell )^n_{\gamma ,\beta } = \left( L (\ell )^n\right) _{\gamma ,\beta }$)

$$\begin{aligned} \begin{aligned} Z_{\lambda }^* c_\gamma (\ell ) Z_{\lambda } = \;&\sum _{n=0}^{n_0} \frac{\lambda ^n}{n!} \sum _{\beta \in \mathcal {I}_\ell } L (\ell )^n_{\gamma ,\beta } \, c_\beta (\ell ) + \sum _{\beta \in \mathcal {I}_\ell } L (\ell )^{n_0+1}_{\gamma ,\beta } \int _0^\lambda {\text{ d }}\tau \frac{(\lambda - \tau )^{n_0}}{n_0!} \, Z_{\tau }^* c_\beta (\ell ) Z_{\tau }\\&+ \sum _{n=0}^{n_0} \sum _{k \in \Gamma ^{\text{ nor }}} \sum _{\beta \in \mathcal {I}_k \cap \mathcal {I}_\ell } \sum _{\alpha \in \mathcal {I}_k} L (\ell )^n_{\gamma ,\beta } L (k)_{\beta , \alpha } \int _0^\lambda {\text{ d }}\tau \frac{(\lambda -\tau )^n}{n!} \, Z_{\tau }^* \mathcal {E}_\beta (\ell ,k) c_\alpha (k) Z_{\tau } \end{aligned} \end{aligned}$$

where, in the last line, for $n=0$, we have $L(\ell )^0_{\gamma ,\beta } = \delta _{\gamma ,\beta }$. Thus, completing the first sum to reconstruct the exponential, we have

$$\begin{aligned} Z_{\lambda }^* c_\gamma (\ell ) Z_{\lambda } = \sum _{\beta \in \mathcal {I}_\ell } \exp (\lambda L (\ell ))_{\gamma ,\beta } \, c_\beta (\ell ) + \mathfrak {F}_{\gamma } (\lambda , \ell ) \end{aligned}$$

with error term

$$\begin{aligned} \begin{aligned} \mathfrak {F}_\gamma (\lambda ,\ell ) = \;&- \sum _{n=n_0+1}^\infty \frac{\lambda ^n}{n!} \sum _{\beta \in \mathcal {I}_\ell } L(\ell )^n_{\gamma ,\beta } \, c_\beta (\ell ) + \sum _{\beta \in \mathcal {I}_\ell } L (\ell )^{n_0+1}_{\gamma ,\beta } \int _0^\lambda {\text{ d }}\tau \frac{(\lambda - \tau )^{n_0}}{n_0!} \, Z_{\tau }^* c_\beta (\ell ) Z_{\tau } \\ {}&+ \sum _{n=0}^{n_0} \sum _{k \in \Gamma ^{\text{ nor }}} \sum _{\beta \in \mathcal {I}_\ell \cap \mathcal {I}_k} \sum _{\alpha \in \mathcal {I}_k} L (\ell )^n_{\gamma ,\beta } L (k)_{\beta , \alpha } \int _0^\lambda {\text{ d }}\tau \frac{(\lambda -\tau )^n}{n!} \, Z_{\tau }^* \mathcal {E}_\beta (\ell ,k) c_\alpha (k) Z_{\tau } \end{aligned} \end{aligned}$$

for an arbitrary $n_0 \in \mathbb {N}$. This error term can be estimated by

7.46 $$\begin{aligned}&\sum _{\gamma \in \mathcal {I}_\ell } \Vert \mathfrak {F}_\gamma (\lambda , \ell ) \psi \Vert \nonumber \\&\quad \leqq \; \sum _{n > n_0} \frac{\lambda ^n}{n!} \sum _{\gamma ,\beta \in \mathcal {I}_\ell } |L(\ell )^n_{\gamma ,\beta }| \Vert c_\beta (\ell ) \psi \Vert + \sum _{\gamma ,\beta \in \mathcal {I}_\ell } | L(\ell )^{n_0+1}_{\gamma ,\beta }| \int _0^\lambda {\text{ d }}\tau \frac{(\lambda -\tau )^{n_0}}{n_0!} \, \Vert c_\beta (\ell ) Z_{\tau } \psi \Vert \nonumber \\&\qquad + \sum _{n=0}^{n_0} \sum _{k \in \Gamma ^{\text{ nor }}} \sum _{\gamma \in \mathcal {I}_\ell , \beta \in \mathcal {I}_k \cap \mathcal {I}_\ell , \alpha \in \mathcal {I}_k} |L (\ell )^n_{\gamma ,\beta } | |L(k)_{\beta , \alpha }| \int _0^\lambda {\text{ d }}\tau \frac{(\lambda -\tau )^n}{n!} \, \Vert \mathcal {E}_\beta (\ell ,k) c_\alpha (k) Z_{\tau } \psi \Vert \nonumber \\&\quad =: \; \text {I} + \text {II} + \text {III} \;. \end{aligned}$$

We estimate that

$$\begin{aligned} \text {I} \leqq M^{1/2} \sum _{n > n_0} \frac{\lambda ^n}{n!} \Vert L (\ell )^n \Vert _{\text{ HS }}\bigg ( \sum _{\beta \in \mathcal {I}_\ell } \Vert c_\beta (\ell ) \psi \Vert ^2 \bigg )^{1/2}\;. \end{aligned}$$

With Lemma 7.2, we obtain $\Vert L(\ell )^n \Vert _{\text{ HS }}\leqq C^n$, uniformly in *N* and $\ell$. From Lemma 5.3,

7.47 $$\begin{aligned} \text {I} \leqq M^{1/2} \Vert \mathcal {N}_\delta ^{1/2} \psi \Vert \sum _{n > n_0} \frac{C^n}{n!} \;. \end{aligned}$$

Similarly, using the invariance of $\mathcal {N}$ with respect to conjugation with $Z_\tau$, we find that

7.48 $$\begin{aligned} \text {II} \leqq \frac{C^{n_0}}{n_0!} M^{1/2} \int _0^\lambda {\text{ d }}\tau \Vert \mathcal {N}_\delta ^{1/2} Z_\tau \psi \Vert \leqq \frac{C^{n_0}}{n_0!} M^{1/2} \Vert \mathcal {N}^{1/2} \psi \Vert \;. \end{aligned}$$

Let us finally consider the last term on the right-hand side of (7.46). We have

$$\begin{aligned} \begin{aligned}&\text{ III } \leqq \sum _{n=0}^\infty \frac{\lambda ^n}{n!} \sum _{k \in \Gamma ^{\text { nor }}} \Bigg ( \sum _{\begin{array}{c} \gamma \in \mathcal {I}_\ell ,\\ \alpha \in \mathcal {I}_k,\\ \beta \in \mathcal {I}_k \cap \mathcal {I}_\ell \end{array}} |L (\ell )^n_{\gamma ,\beta }|^2 |L(k)_{\beta , \alpha }|^2 \Bigg ) ^{1/2} \\ {}&\quad \times \int _0^\lambda {\text { d }}\tau \Bigg ( \sum _{\begin{array}{c} \gamma \in \mathcal {I}_\ell ,\\ \alpha \in \mathcal {I}_k,\\ \beta \in \mathcal {I}_k \cap \mathcal {I}_\ell \end{array}} \Vert \mathcal {E}_\beta (k,\ell ) c_\alpha (k) Z_{\tau } \psi \Vert ^2 \Bigg ) ^{1/2}\!\!.\end{aligned} \end{aligned}$$

Using

$$\begin{aligned}\begin{aligned} \Bigg ( \sum _{\begin{array}{c} \gamma \in \mathcal {I}_\ell ,\\ \alpha \in \mathcal {I}_k,\\ \beta \in \mathcal {I}_k \cap \mathcal {I}_\ell \end{array}} |L (\ell )^n_{\gamma ,\beta }|^2 |L(k)_{\beta , \alpha }|^2 \Bigg ) ^{1/2}&\leqq \Vert L (\ell )^n \Vert _{\text { HS }}\Vert L(k) \Vert _{\text { HS }}\leqq C^n \hat{V} (k) \;, \end{aligned} \end{aligned}$$

the bound (5.7), the relation $\mathcal {N}c_\alpha (k) = c_\alpha (k) (\mathcal {N}-2)$, and Lemma 5.3, we find that

$$\begin{aligned} \text {III} \leqq C \sum _{k \in \Gamma ^{\text{ nor }}} \hat{V} (k) N^{-2/3+ \delta } M^{3/2} \int _0^\lambda {\text{ d }}\tau \, \Vert \, \mathcal {N}^{1/2}_\delta \mathcal {N}Z_\tau \psi \Vert \; . \end{aligned}$$

With $\Vert \hat{V} \Vert _1 < \infty$ and Lemma 7.3, we conclude that

7.49 $$\begin{aligned} \text {III} \leqq C N^{-2/3+ \delta } M^{3/2} \Vert \mathcal {N}_\delta ^{1/2} \mathcal {N}\psi \Vert \; . \end{aligned}$$

Since the right-hand side of both (7.47) and (7.48) vanishes as $n_0 \rightarrow \infty$ (and since (7.49) does not depend on $n_0$), we arrive at (7.45). $\square$

## Linearization of the Kinetic Energy

We will use Lemma 7.5 to show that (7.13) and (7.14) hold approximately true on states with few excitations. What is still missing to conclude the argument explained in Sect. 2 is the invariance of $\mathbb {H}_0 - \mathbb {D}_\text{ B }$ with respect to the action of the approximate Bogoliubov transformations (7.11). The proof is based on the fact that the commutators of $\mathbb {H}_0$ and $\mathbb {D}_\text{ B }$ with the $c^*$-operators are approximately the same, as described by the following lemma:

### Lemma 8.1

(Kinetic commutators) Let $RM^{1/2} \leqq N^{1/3}$. For all $k \in \Gamma ^{\text{ nor }}$ and all $\alpha \in \mathcal {I}_k$, we have

8.1 $$\begin{aligned} \begin{aligned} {[}\mathbb {H}_0 , c_\alpha ^* (k) ]&= 2\hbar \kappa |k \cdot \hat{\omega }_\alpha |c_\alpha ^* (k) + \hbar \mathfrak {E}_\alpha ^\text{ lin } (k)^* \\ {[}\mathbb {D}_\text{ B } , c_\alpha ^* (k) ]&= 2\hbar \kappa |k \cdot \hat{\omega }_\alpha |c_\alpha ^* (k) + \hbar \mathfrak {E}^\text{ B}_\alpha (k)^* \end{aligned} \end{aligned}$$

where there exists a $C > 0$ such that for all $f \in \ell ^2({\mathcal {I}_{k}})$ and all $\psi \in \mathcal {F}$ we have

8.2 $$\begin{aligned} \begin{aligned} \sum _{\alpha \in \mathcal {I}_k} \Big \Vert \mathfrak {E}_\alpha ^\text{ lin } (k) \psi \Big \Vert&\leqq C |k|\Vert \mathcal {N}_\delta ^{1/2} \psi \Vert \;,\\ \Big \Vert \sum _{\alpha \in \mathcal {I}_k} f_\alpha \mathfrak {E}_\alpha ^\text{ lin } (k) \psi \Big \Vert&\leqq C |k|M^{-1/2} \Vert f \Vert _2 \Vert \mathcal {N}_\delta ^{1/2} \psi \Vert \;,\\ \sum _{\alpha \in \mathcal {I}_k} \Vert \mathfrak {E}_\alpha ^{\text{ B }} (k) \psi \Vert&\leqq C R^3 M^{3/2} N^{-2/3+\delta } \Vert \mathcal {N}^{1/2}_\delta \mathcal {N}\psi \Vert \;. \end{aligned} \end{aligned}$$

### Proof

The bounds for $\mathfrak {E}_\alpha ^\text{ lin }$ are shown as in [5, Lemma 8.2], keeping track of the *k*-dependence. From (2.1) we get

$$\begin{aligned} {[}\mathbb {H}_0,c^*_{\alpha }(k)]&= \frac{1}{n_{\alpha }(k)} \sum _{\begin{array}{c} p:p\in B_\text{ F}^c\cap B_\alpha \\ p-k \in B_\text{ F }\cap B_\alpha \end{array}} (e(p)+e(p-k))a^*_p a^*_{p-k} \\ {}&= 2\hbar \kappa |k\cdot \hat{\omega }_\alpha |c_\alpha ^*(k) + \hbar \mathfrak {E}^{\text{ lin }}_\alpha (k)^* \;, \end{aligned}$$

where, using the weighted pair operators as in Lemma 5.3, $\mathfrak {E}^{\text{ lin }}_\alpha (k) = c^{g}_{\alpha }(k)$ with

$$\begin{aligned} g(p,k) = \hbar ^{-1} \Big ( e(p)+e(p-k) - 2\hbar \kappa |k\cdot \hat{\omega }_\alpha |\Big ) = \hbar \Big ( 2 k\cdot (p - k_\text{ F }\hat{\omega }_\alpha ) -|k|^2 \Big )\;. \end{aligned}$$

Since $B_\alpha$ has diameter of order $N^{1/3} M^{-1/2}$ on the Fermi surface and since *p* can be at most at distance |*k*| from the Fermi surface, we can bound (using the assumption $|k| M^{1/2} \leqq R M^{1/2} \leqq N^{1/3}$)

$$\begin{aligned} |g(p,k) |\leqq C \hbar |k|\left( |p - k_\text{ F }\hat{\omega }_\alpha |+ |k|\right) \leqq C |k| M^{-1/2} \;. \end{aligned}$$

The first two estimates in (8.2) follow from (5.11) and (5.12).

The last bound in (8.2) is shown exactly as in [5, Eq. (8.6)], using the bound $|\Gamma ^{\text{ nor }}| \leqq C R^3$ to sum over $l \in \Gamma ^{\text{ nor }}$ there. $\square$

The invariance with respect to *T* is established in the next lemma. This lemma can be shown as [5, Lemma 8.1], replacing bounds for $\mathfrak {E}_\alpha ^\text{ lin }$ and $\mathfrak {E}_\alpha ^{\text{ B }}$ with those established in Lemma 8.1 (and using the assumption $\sum _{k} \hat{V} (k) |k| < \infty$). We skip any further details.

### Lemma 8.2

(Approximate *T*-invariance of $\mathbb {H}_0 - \mathbb {D}_\text{ B }$) Let $\sum _{k \in \mathbb {Z}^3} |\hat{V}(k)|\left( 1 + |k|\right) < \infty$. Then there exists a $C > 0$ such that for all $\psi \in \mathcal {F}$ we have

$$\begin{aligned} \begin{aligned}&|\langle T \psi , (\mathbb {H}_0 - \mathbb {D}_\text{ B}) T \psi \rangle - \langle \psi , (\mathbb {H}_0 - \mathbb {D}_\text{ B}) \psi \rangle |\\&\quad \leqq C \hbar \left( M^{-1/2} \Vert (\mathcal {N}_\delta +1)^{1/2} \psi \Vert ^2 + R^3 M N^{-2/3+\delta } \Vert \mathcal {N}_\delta ^{1/2} (\mathcal {N}+1) \psi \Vert \Vert (\mathcal {N}_\delta + 1)^{1/2} \psi \Vert \right) \,. \end{aligned} \end{aligned}$$

In the next lemma, we use (8.2) to show the approximate invariance of $\mathbb {H}_0 - \mathbb {D}_\text{ B }$ with respect to the action of the transformation *Z* defined in (7.11).

### Lemma 8.3

(Approximate *Z*-invariance of $\mathbb {H}_0 - \mathbb {D}_\text{ B }$) Let $\sum _{k \in \mathbb {Z}^3} |\hat{V}(k)|\left( 1 + |k|\right) < \infty$. Then there exists a $C >0$ such that for all $\psi \in \mathcal {F}$ we have

$$\begin{aligned} \begin{aligned}&|\langle Z \psi , (\mathbb {H}_0 - \mathbb {D}_\text{ B}) Z \psi \rangle - \langle \psi , (\mathbb {H}_0 - \mathbb {D}_\text{ B}) \psi \rangle |\\&\quad \leqq C \hbar \left( M^{-1/2} \Vert \mathcal {N}_\delta ^{1/2} \psi \Vert ^2 + R^3 M^{3/2} N^{-2/3+ \delta } \Vert \mathcal {N}_\delta ^{1/2} \mathcal {N}^{1/2} \psi \Vert \Vert \mathcal {N}_\delta ^{1/2} \psi \Vert \right) \,. \end{aligned} \end{aligned}$$

### Proof

Recalling the Definition (7.39) of the operators $Z_\lambda$, we compute that

$$\begin{aligned} \frac{{\text{ d }}}{{\text{ d }}\lambda } \langle Z_\lambda \psi , (\mathbb {H}_0 - \mathbb {D}_\text{ B}) Z_\lambda \psi \rangle = \sum _{k \in \Gamma ^{\text{ nor }}} \sum _{\alpha , \beta \in \mathcal {I}_k} L_{\alpha ,\beta } (k) \langle Z_\lambda \psi , \left[ c_\alpha ^* (k) c_\beta (k) , (\mathbb {H}_0 - \mathbb {D}_\text{ B}) \right] Z_\lambda \psi \rangle \;. \end{aligned}$$

With (8.1) we obtain

$$\begin{aligned} \begin{aligned}&\hbar ^{-1} \frac{{\text{ d }}}{{\text{ d }}\lambda } \langle Z_\lambda \psi , (\mathbb {H}_0 - \mathbb {D}_\text{ B}) Z_\lambda \psi \rangle \\&\quad = - \sum _{k \in \Gamma ^{\text{ nor }}} \sum _{\alpha , \beta \in \mathcal {I}_k} L_{\alpha ,\beta } (k) \langle Z_\lambda \psi , (\mathfrak {E}_\alpha ^{\text{ lin }} (k) - \mathfrak {E}_\alpha ^\text{ B } (k))^* c_\beta (k) Z_\lambda \psi \rangle \\&\qquad - \sum _{k \in \Gamma ^{\text{ nor }}} \sum _{\alpha , \beta \in \mathcal {I}_k} L_{\alpha ,\beta } (k) \langle Z_\lambda \psi , c^*_\alpha (k) (\mathfrak {E}_\beta ^\text{ lin } (k) - \mathfrak {E}_\beta ^\text{ B } (k)) Z_\lambda \psi \rangle \;. \end{aligned} \end{aligned}$$

Hence

$$\begin{aligned} \begin{aligned}&\left| \hbar ^{-1} \frac{{\text{ d }}}{{\text{ d }}\lambda } \langle Z_\lambda \psi , (\mathbb {H}_0 - \mathbb {D}_\text{ B}) Z_\lambda \psi \rangle \right| \leqq \; \sum _{k \in \Gamma ^{\text{ nor }}} \sum _{\beta \in \mathcal {I}_{k}} \Big \Vert \sum _{\alpha \in \mathcal {I}_{k}} L_{\alpha ,\beta } (k) \mathfrak {E}_\alpha ^\text{ lin } (k) Z_\lambda \psi \Big \Vert \Vert c_\beta (k) Z_\lambda \psi \Vert \\&\quad +\sum _{k \in \Gamma ^{\text{ nor }}} \sum _{\alpha \in \mathcal {I}_{k}} \Vert \mathfrak {E}_\alpha ^\text{ B } (k) Z_\lambda \psi \Vert \Big \Vert \sum _{\beta \in \mathcal {I}_{k}} L_{\alpha ,\beta } (k) c_\beta (k) Z_\lambda \psi \Big \Vert \;. \end{aligned} \end{aligned}$$

Using Lemma 8.1 (and $\Vert L_{\alpha ,\cdot }(k) \Vert _2 \leqq \Vert L(k) \Vert _{\text{ HS }}$ for all $\alpha \in \mathcal {I}_k$), we conclude that

$$\begin{aligned} \begin{aligned}&\Big |\hbar ^{-1} \frac{{\text{ d }}}{{\text{ d }}\lambda } \langle Z_\lambda \psi , (\mathbb {H}_0 - \mathbb {D}_\text{ B}) Z_\lambda \psi \rangle \Big | \\&\quad \leqq \; \sum _{k \in \Gamma ^{\text{ nor }}} C M^{-1/2} |k|\sum _{\beta \in \mathcal {I}_{k}} \Vert L_{\cdot , \beta } (k) \Vert _2 \Vert c_\beta (k) Z_\lambda \psi \Vert \Vert \mathcal {N}_\delta ^{1/2} Z_\lambda \psi \Vert \\&\qquad + \sum _{k \in \Gamma ^{\text{ nor }}} \sum _{\alpha \in \mathcal {I}_{k}} \Vert L_{\alpha , \cdot } (k) \Vert _2 \Vert \mathfrak {E}_\alpha ^\text {B} (k) Z_\lambda \psi \Vert \Vert \mathcal {N}_\delta ^{1/2} Z_\lambda \psi \Vert \\&\quad \leqq \; CM^{-1/2} \sum _{k \in \Gamma ^{\text{ nor }}} |k|\Vert L(k)\Vert _{\text{ HS }}\Vert \mathcal {N}_\delta ^{1/2} Z_\lambda \psi \Vert ^2 \\&\qquad + C R^3 M^{3/2} N^{-2/3+\delta }\sum _{k \in \Gamma ^{\text{ nor }}} \Vert L (k) \Vert _{\text{ HS }}\Vert \mathcal {N}^{1/2}_\delta \mathcal {N}Z_\lambda \psi \Vert \Vert \mathcal {N}_\delta ^{1/2} Z_\lambda \psi \Vert \;. \end{aligned} \end{aligned}$$

With Lemmas 7.2 and 7.3 we obtain (since $\sum _{k \in \mathbb {Z}^3} |\hat{V}(k)|\left( 1 + |k|\right) < \infty$)

$$\begin{aligned} \begin{aligned} \left| \hbar ^{-1} \frac{{\text{ d }}}{{\text{ d }}\lambda } \langle Z_\lambda \psi , (\mathbb {H}_0 - \mathbb {D}_\text{ B}) Z_\lambda \psi \rangle \right|&\leqq CM^{-1/2} \Vert \mathcal {N}_\delta ^{1/2} \psi \Vert ^2 \\&\quad + C R^ 3M^{3/2} N^{-2/3+\delta } \Vert \mathcal {N}_\delta ^{1/2} \mathcal {N}\psi \Vert \Vert \mathcal {N}_\delta ^{1/2} \psi \Vert \;. \end{aligned} \end{aligned}$$

Integrating over $\lambda \in [0,1]$ we arrive at the desired bound. $\square$

## Proof of Theorem 1.1

We use the next proposition for localization in particle number sectors of Fock space. It is taken from [23, Prop. 6.1] (given there for bosonic Fock space, but inspection of the proof shows that the symmetry/antisymmetry of the wave function does not play any role).

### Proposition 9.1

(Particle number localization) Let $\mathcal {A}$ be a non-negative operator on $\mathcal {F}$ with $P_i D(\mathcal {A}) \subset D(\mathcal {A})$ and $P_i \mathcal {A}P_j = 0$ if $|i-j| > \ell$, where $P_i = \chi (\mathcal {N}= i)$. Let $f,g : [0 , \infty ) \rightarrow [0,1]$ be smooth functions with $f^2 + g^2 = 1$, $f(x) = 1$ for $x \leqq 1/2$, and $f(x) = 0$ for $x \geqq 1$. For $L \geqq 1$, let $f_L := f (\mathcal {N}/ L)$ and $g_L := g (\mathcal {N}/L)$.

Then, there exists a $C > 0$ (one can take $C := 2 (\Vert f' \Vert _\infty ^2 + \Vert g' \Vert _\infty ^2)$) such that

$$\begin{aligned} - \frac{C \ell ^3}{L^2} \mathcal {A}_\text {diag} \leqq \mathcal {A}- f_L \mathcal {A}f_L - g_L \mathcal {A}g_L \leqq \frac{C \ell ^3}{L^2} \mathcal {A}_\text {diag} \end{aligned}$$

where $$\mathcal {A}_\text {diag} = \sum _{i=0}^\infty P_i \mathcal {A}P_i$$.

We turn to the proof of our main result.

### Proof of Theorem 1.1

The main work is for the proof of the lower bound; the upper bound follows from the same operator estimates but using a specific trial state, for which the errors are easier to control.

**Lower bound.** Let $\psi _\text {gs}$ be a normalized ground state vector for the Hamilton operator $H_N$ in (1.1). Since the Hartree–Fock energy arises from a restriction of the many-body variational problem to a smaller set, we have

$$\begin{aligned} \langle \psi _\text {gs} , H_N \psi _\text {gs} \rangle \leqq E_N^\text{ HF } \;.\end{aligned}$$

Let $\xi _\text {gs} = R^* \psi _\text {gs}$ denote the excitation vector associated with $\psi _\text {gs}$, defined through the unitary particle–hole transformation (2.3). From the Definition (2.4) of the correlation Hamiltonian we have $\langle \xi _\text {gs}, \mathcal {H}_\text{ corr } \xi _\text {gs} \rangle \leqq 0$. With Lemma 4.1 and Corollary 4.6, we find a $C > 0$ such that

9.1 $$\begin{aligned} \begin{aligned} \langle \xi _\text {gs} , \mathbb {H}_0 \xi _\text {gs} \rangle \leqq C \hbar \;, \quad \langle \xi _\text {gs}, Q_\text{ B } \xi _\text {gs} \rangle \leqq C \hbar \;, \quad \langle \xi _\text {gs} , \mathcal {E}_1 \xi _\text {gs} \rangle \leqq C \hbar \;. \end{aligned} \end{aligned}$$

The last bound follows because from Lemma 4.7 and Corollary 4.9 we get $\mathcal {E}_1 \leqq C (\mathcal {H}_\text{ corr } + \mathbb {H}_0 + \hbar )$. Furthermore, from Corollary 4.2, we have

9.2 $$\begin{aligned} \langle \xi _\text {gs} , \mathcal {N}\xi _\text {gs} \rangle \leqq C N^{1/3}\; , \quad \langle \xi _\text {gs} , \mathcal {N}_\varepsilon \xi _\text {gs} \rangle \leqq C N^\varepsilon \qquad \text{ for } \text{ every }\, \varepsilon > 0. \end{aligned}$$

Next we localize with respect to the number of particles. We choose smooth functions *f* and *g* as in Proposition 9.1 and set $f_N := f (\mathcal {N}/ C_0 N^{1/3})$, $g_N := g (\mathcal {N}/ C_0 N^{1/3})$ for a constant $C_0 > 0$ large enough, to be fixed below. We set $\mathcal {A}= \mathcal {H}_\text{ corr } + C \hbar$, with $C > 0$ large enough. From Lemma 4.1 we get $\mathcal {A}\geqq 0$. From the Definition (2.4) of $\mathcal {H}_\text{ corr }$, combined with the bounds in Corollary 4.6 for the operator $Q_\text{ B }$, in Lemma 4.7 for the exchange operator $\mathbb {X}$ and in Corollary 4.9 for the error term $\mathcal {E}_2$, we conclude that

$$\begin{aligned} \mathcal {A}\leqq C (\mathbb {H}_0 + \mathcal {E}_1 + \hbar ) \;. \end{aligned}$$

Since $\mathbb {H}_0$ and $\mathcal {E}_1$ both commute with $\mathcal {N}$, it also follows that $\mathcal {A}_\text {diag} \leqq C (\mathbb {H}_0 + \mathcal {E}_1 + \hbar )$. From Proposition 9.1 (since, with the notation introduced in the proposition, $P_i \mathcal {A}P_j = 0$ if $|i-j| > 4$), we find that

$$\begin{aligned} - C N^{-2/3} (\mathbb {H}_0 + \mathcal {E}_1 + \hbar )\leqq & {} \mathcal {H}_\text{ corr } - f_N \mathcal {H}_\text{ corr } f_N - g_N \mathcal {H}_\text{ corr } g_N\\ {}\leqq & {} C N^{-2/3} (\mathbb {H}_0 + \mathcal {E}_1 + \hbar ) \;. \end{aligned}$$

We apply this bound to the ground state $\xi _\text {gs}$. From the a-priori bounds in (9.1), we obtain

9.3 $$\begin{aligned} \langle \xi _\text {gs} , \mathcal {H}_\text{ corr } \xi _\text {gs} \rangle \geqq \langle \xi _\text {gs} , f_N \mathcal {H}_\text{ corr } f_N \xi _\text {gs} \rangle + \langle \xi _\text {gs} , g_N \mathcal {H}_\text{ corr } g_N \xi _\text {gs} \rangle - C N^{-1} \;. \end{aligned}$$

Since $\xi _\text {gs}$ is the ground state vector of $\mathcal {H}_\text{ corr }$, we can estimate

$$\begin{aligned} \langle \xi _\text {gs} , g_N \mathcal {H}_\text{ corr } g_N \xi _\text {gs} \rangle \geqq \Vert g_N \xi _\text {gs} \Vert ^2 \, \langle \xi _\text {gs}, \mathcal {H}_\text{ corr } \xi _\text {gs} \rangle \;. \end{aligned}$$

With (9.3) (and since $f^2 + g^2 = 1$), we arrive at

9.4 $$\begin{aligned} \Vert f_N \xi _\text {gs} \Vert ^2 \langle \xi _\text {gs} , \mathcal {H}_\text{ corr } \xi _\text {gs} \rangle \geqq \langle f_N \xi _\text {gs} , \mathcal {H}_\text{ corr } f_N \xi _\text {gs} \rangle - C N^{-1}\;. \end{aligned}$$

From (9.2), we have, fixing $C_0$ large enough,

$$\begin{aligned} \Vert g_N \xi _\text {gs} \Vert ^2 = \langle \xi _\text {gs}, g^2 (\mathcal {N}/ C_0 N^{1/3}) \xi _\text {gs} \rangle \leqq \frac{1}{C_0 N^{1/3}} \langle \xi _\text {gs} , \mathcal {N}\xi _\text {gs} \rangle \leqq \frac{1}{2} \;. \end{aligned}$$

Hence $\Vert f_N \xi _\text {gs} \Vert ^2 \geqq 1/2$ and, from (9.4),

9.5 $$\begin{aligned} \langle \xi _\text {gs} , \mathcal {H}_\text{ corr } \xi _\text {gs} \rangle \geqq \langle \xi , \mathcal {H}_\text{ corr } \xi \rangle - C N^{-1} \end{aligned}$$

where we defined $\xi = f_N \xi _\text {gs} / \Vert f_N \xi _\text {gs} \Vert \in \chi (\mathcal {N}_\text{ p } - \mathcal {N}_\text{ h } = 0) \mathcal {F}$ (particle number localization leaves the space invariant, since $\mathcal {N}_\text{ p }$ and $\mathcal {N}_\text{ h }$ commute with $\mathcal {N}$). Like $\xi _\text {gs}$, the localized vector $\xi$ satisfies $\langle \xi , \mathcal {H}_\text{ corr } \xi \rangle \leqq C \hbar$ and therefore by Lemma 4.1 we get

9.6 $$\begin{aligned} \langle \xi , \mathbb {H}_0 \xi \rangle \leqq C \hbar \;. \end{aligned}$$

The advantage of working with $\xi$ is that it satisfies stronger bounds (compared with $\xi _\text {gs}$) on the number of particles. In fact, we find that

9.7 $$\begin{aligned} \langle \xi , \mathcal {N}^m \xi \rangle \leqq C^m N^{m/3} , \quad \langle \xi \;, \mathcal {N}^m \mathcal {N}_\varepsilon \xi \rangle \leqq C^m N^{\varepsilon + m/3} \end{aligned}$$

for every $m \in \mathbb {N}$ and $\varepsilon > 0$ (to prove the second estimate, we used $[ \mathcal {N}, \mathcal {N}_\varepsilon ] = 0$).

From (9.5), to conclude the proof of the lower bound, it is therefore enough to show that $\langle \xi , \mathcal {H}_\text{ corr } \xi \rangle \geqq E_N^\text{ RPA } - C N^{-1/3-\alpha }$, for sufficiently small $\alpha > 0$ and for all $\xi \in \chi (\mathcal {N}_\text{ p } - \mathcal {N}_\text{ h } = 0) \mathcal {F}$ satisfying (9.6) and (9.7). For such vectors, it follows from Lemma 4.7, Corollary 4.9 and Lemma 6.1 that, for any sufficiently small $\varepsilon , \delta > 0$ and for $N^{2\delta } \ll M \ll N^{2/3-2\delta }$,

9.8 $$\begin{aligned} \begin{aligned} \langle \xi , \mathcal {H}_\text { corr } \xi \rangle \geqq \;&\langle \xi , (\mathbb {H}_0 + Q_\text { B}^R) \xi \rangle \\ {}&- C \hbar \Big ( N^{-1/3} + N^{-\varepsilon /4} + N^{-(1-\gamma )/3 + 5\varepsilon /4} + N^{-\delta /2} \\ {}&\qquad + R^{1/2} M^{1/4} N^{-1/6+ \delta /2} + R^{-1/2} \Big ) \end{aligned} \end{aligned}$$

with the quadratic expression $Q_\text{ B}^R$ defined in (6.1) (notice that the definition of $Q_\text{ B}^R$ depends on $\delta$). Using the notation introduced in (6.3) and in (6.5), we can write

9.9 $$\begin{aligned} \langle \xi , (\mathbb {H}_0 + Q_\text{ B}^R) \xi \rangle = \langle \xi , (\mathbb {H}_0 - \mathbb {D}_\text{ B}) \xi \rangle + \sum _{k \in \Gamma ^{\text{ nor }}} 2\hbar \kappa |k| \langle \xi , h_\text {eff} (k) \xi \rangle \;. \end{aligned}$$

Next, we diagonalize the quadratic Hamiltonian $h_\text {eff} (k)$ by means of the approximate Bogoliubov transformations defined in Sect. 7. Recalling (7.11), we define $\eta = Z^* T^* \xi \in \chi (\mathcal {N}_\text{ p } - \mathcal {N}_\text{ h } = 0) \mathcal {F}$. From (9.7) and from Lemma 7.3, we can control the number of particles in $\eta$ and $Z \eta = T^* \xi$: for every $m \in \mathbb {N}$ we find a $C > 0$ such that

9.10 $$\begin{aligned} \langle \eta , \mathcal {N}^m \eta \rangle&\leqq C N^{m/3}\;,&\langle Z \eta , \mathcal {N}^m Z \eta \rangle&\leqq C N^{m/3}\;, \end{aligned}$$

9.11 $$\begin{aligned} \langle \eta , \mathcal {N}^m \mathcal {N}_\delta \eta \rangle&\leqq C N^{\delta + m/3}\;,&\langle Z \eta , \mathcal {N}^m \mathcal {N}_\delta Z \eta \rangle&\leqq C N^{\delta + m/3} \;. \end{aligned}$$

Writing $\xi = T Z \eta$ and applying Lemma 8.2 and Lemma 8.3, we obtain

9.12 $$\begin{aligned} \langle \xi , (\mathbb {H}_0 - \mathbb {D}_\text{ B}) \xi \rangle&= \langle T Z \eta , (\mathbb {H}_0 - \mathbb {D}_\text{ B}) T Z \eta \rangle \nonumber \\&\geqq \langle \eta , (\mathbb {H}_0 - \mathbb {D}_\text{ B}) \eta \rangle - C \hbar \Big ( M^{-1/2} \Vert (\mathcal {N}_\delta + 1)^{1/2} \eta \Vert ^2 \nonumber \\&\quad + R^3 M^{3/2} N^{-2/3+ \delta } \Vert \mathcal {N}_\delta ^{1/2} (\mathcal {N}+1) \eta \Vert \Vert (\mathcal {N}_\delta + 1)^{1/2} \eta \Vert \Big ) \nonumber \\&\geqq \langle \eta , (\mathbb {H}_0 - \mathbb {D}_\text{ B}) \eta \rangle - C \hbar \left( M^{-1/2} N^\delta + R^3 M^{3/2} N^{-1/3+2\delta } \right) \;. \end{aligned}$$

We now focus on the second term on the right-hand side of (9.9). Writing $\xi = T Z \eta$, we compute first the action of *T*. We proceed here as in the proof of [5, Lemma 10.1]. Analogously to [5, Eqs. (10.13)] we find that

9.13 $$\begin{aligned}&\sum _{k \in \Gamma ^{\text { nor }}} \hbar \kappa |k| \langle \xi , h_\text{ eff } (k) \xi \rangle \nonumber \\ {}&\quad = \sum _{k \in \Gamma ^{\text { nor }}} 2\hbar \kappa |k| \langle T Z \eta , h_\text{ eff } (k) T Z \eta \rangle \nonumber \\ {}&\quad \geqq \sum _{k \in \Gamma ^{\text { nor }}} \hbar \kappa |k|{\text{ tr }}\left( E(k) - D(k) - W(k) \right) + \sum _{k \in \Gamma ^{\text { nor }}} \sum _{\alpha ,\beta \in \mathcal {I}_k} 2\hbar \kappa |k| \, \mathfrak {K} (k)_{\alpha , \beta } \langle Z \eta , c_\alpha ^* (k) c_\beta (k) Z \eta \rangle \nonumber \\ {}&\qquad - C\hbar \Big ( N^{-2/3+\delta } \Vert \mathcal {N}^{1/2} Z \eta \Vert ^2 + M R^4 N^{-2/3+\delta } \Vert (\mathcal {N}_\delta +1)^{1/2} Z \eta \Vert \Vert (\mathcal {N}_\delta + M)^{1/2} (\mathcal {N}+1) Z \eta \Vert \nonumber \\ {}&\qquad \qquad + M^2 R^4 N^{-4/3 + 2\delta } \Vert (\mathcal {N}_\delta + M)^{1/2} (\mathcal {N}+1) Z \eta \Vert ^2 \Big ) \end{aligned}$$

where we introduced the $|\mathcal {I}_k| \times |\mathcal {I}_k|$ matrix $\mathfrak {K}$ by

$$\begin{aligned} \begin{aligned} \left( \begin{array}{ll} \mathfrak {K} (k) &{} 0 \\ 0 &{} \mathfrak {K} (k) \end{array} \right)&:= \left( \begin{array}{ll} \cosh (K(k)) &{} \sinh (K(k)) \\ \sinh (K(k)) &{} \cosh (K(k)) \end{array} \right) \\&\quad \times \left( \begin{array}{ll} D(k) + W(k) &{} \widetilde{W} (k) \\ \widetilde{W} (k) &{} D(k) + W(k) \end{array} \right) \left( \begin{array}{ll} \cosh (K(k)) &{} \sinh (K(k)) \\ \sinh (K(k)) &{} \cosh (K(k)) \end{array} \right) \,. \end{aligned} \end{aligned}$$

Comparing with (7.4), we find $\mathfrak {K}(k) = O(k) E(k) O(k)^T$. The first error term in the square brackets on the right-hand side of ((9.13)) arises from [5, Eq. (10.10)], a bound which holds under the assumption $\Vert \hat{V} \Vert _1 < \infty$; this follows from the observation that [5, Eq. (10.9)] can be improved to

$$\begin{aligned} \begin{aligned}&\Big | \big [ 2 \sinh (K(k)) (D(k) + W(k)) \sinh (K(k)) + \cosh (K(k)) \widetilde{W} (k) \sinh (K(k)) \\&\quad + \sinh (K(k)) \widetilde{W} (k) \cosh (K(k)) \big ]_{\alpha , \alpha } \Big | \leqq C \hat{V} (k) M^{-1} \; . \end{aligned} \end{aligned}$$

The further two error terms in the square brackets arise from [5, Eq. (10.6)]; this estimate holds for every fixed *k*. The sum over $k \in \Gamma ^{\text{ nor }}$ gives the additional factor $R^4$. Using (9.10) and Lemma 9.2 (and recalling $M \gg N^{2\delta }$) we find that

9.14 $$\begin{aligned}&\sum _{k \in \Gamma ^{\text { nor }}} 2\hbar \kappa |k| \langle \xi , h_\text{ eff } (k) \xi \rangle \geqq E_N^\text { RPA } + \sum _{k \in \Gamma ^{\text { nor }}} \sum _{\alpha ,\beta \in \mathcal {I}_k} 2\hbar \kappa |k| \, \mathfrak {K} (k)_{\alpha , \beta } \langle Z \eta , c_\alpha ^* (k) c_\beta (k) Z \eta \rangle \nonumber \\ {}&\quad - C\hbar \Big ( R^2 M^{1/4} N^{-1/6+\delta /2} + N^{-\delta /2} + M^{-1/4} N^{\delta /2} + N^{-1/3+\delta } \nonumber \\ {}&\qquad \qquad + M^{3/2} R^4 N^{-1/3+3\delta /2} + M^{3} R^4 N^{-2/3 + 2\delta } \Big )\,. \end{aligned}$$

Next, we compute the action of the approximate Bogoliubov transformation (approximate unitary transformation in the one-boson Hilbert space) *Z* in the second term on the right-hand side of (9.14). With Lemma 7.5, recalling that $\exp (L(k)) = O(k)\widetilde{O}(k)$, we find that

9.15 $$\begin{aligned} \begin{aligned}&\sum _{k \in \Gamma ^{\text{ nor }}} 2\hbar \kappa |k| \sum _{\alpha ,\beta \in \mathcal {I}_k} \mathfrak {K}_{\alpha ,\beta } (k) \langle \eta , Z^* c_\alpha ^* (k) c_\beta (k) Z \eta \rangle \\&\quad = \sum _{k \in \Gamma ^{\text{ nor }}} 2\hbar \kappa |k| \sum _{\alpha ,\beta \in \mathcal {I}_k} \left[ \widetilde{O}^T(k) O^T (k) \mathfrak {K} (k) O (k) \widetilde{O}(k) \right] _{\alpha ,\beta } \langle \eta , c_\alpha ^* (k) c_\beta (k) \eta \rangle \\&\qquad + \sum _{k \in \Gamma ^{\text{ nor }}} 2\hbar \kappa |k| \sum _{\alpha ,\beta \in \mathcal {I}_k} \left[ \widetilde{O}^T(k) O^T (k) \mathfrak {K} (k) \right] _{\alpha ,\beta } \langle \eta , c_\alpha ^* (k) \mathfrak {F}_\beta (1,k) \eta \rangle \\&\qquad + \sum _{k \in \Gamma ^{\text{ nor }}} 2\hbar \kappa |k| \sum _{\alpha ,\beta \in \mathcal {I}_k} \left[ \mathfrak {K} (k) O (k) \widetilde{O}(k)\right] _{\alpha ,\beta } \langle \eta , \mathfrak {F}^*_\alpha (1,k) c_\beta (k) \eta \rangle \\&\qquad + \sum _{k \in \Gamma ^{\text{ nor }}} 2\hbar \kappa |k| \sum _{\alpha ,\beta \in \mathcal {I}_k} \mathfrak {K} (k)_{\alpha ,\beta } \langle \eta , \mathfrak {F}^*_\alpha (1,k) \mathfrak {F}_\beta (k) \eta \rangle \;. \end{aligned} \end{aligned}$$

By Lemma 7.5 we can show that the contributions on the last three lines are negligible. For example, the second term can be bounded by

$$\begin{aligned} \begin{aligned}&\Big | \sum _{k \in \Gamma ^{\text{ nor }}} 2\hbar \kappa |k| \sum _{\alpha ,\beta \in \mathcal {I}_k} \left[ \widetilde{O}^T(k) O^T (k) \mathfrak {K} (k) \right] _{\alpha ,\beta } \langle \eta , c_\alpha ^* (k) \mathfrak {F}_\beta (1,k) \eta \rangle \Big | \\&\quad \leqq \sum _{k \in \Gamma ^{\text{ nor }}} 2\hbar \kappa |k| \sum _{\beta \in \mathcal {I}_k} \Vert \mathfrak {F}_\beta (1,k) \eta \Vert \Big \Vert \sum _{\alpha \in \mathcal {I}_k} \left[ \widetilde{O}^T(k) O^T (k) \mathfrak {K} (k) \right] _{\alpha ,\beta } c_\alpha (k) \eta \Big \Vert \\&\quad \leqq \sum _{k \in \Gamma ^{\text{ nor }}} 2\hbar \kappa |k| \sum _{\beta \in \mathcal {I}_k} \Vert \mathfrak {F}_\beta (1,k) \eta \Vert \Vert [\widetilde{O}^T(k) O^T (k) \mathfrak {K} (k)]_{\beta , .} \Vert _2 \Vert \mathcal {N}_\delta ^{1/2} \eta \Vert \\&\quad \leqq C N^{-1+ \delta } M^{3/2} \sum _{k \in \Gamma ^{\text{ nor }}} |k| \, \Vert \widetilde{O}^T(k) O^T (k) \mathfrak {K} (k) \Vert _{\text{ HS }}\Vert \mathcal {N}_\delta ^{1/2} \mathcal {N}\eta \Vert \Vert \mathcal {N}_\delta ^{1/2} \eta \Vert \;. \end{aligned} \end{aligned}$$

Recalling $\mathfrak {K} (k) = O (k) E (k) O^T (k)$ and the expression (7.8) for the matrix *E*(*k*), we find

$$\begin{aligned} \Vert \widetilde{O}^T(k) O^T (k) \mathfrak {K} (k) \Vert _{\text{ HS }}= \sqrt{2} \left( {\text {tr}}\; d^2 + 2 {\text {tr}}\; d^{1/2} b d^{1/2} \right) ^{1/2} \leqq C M^{1/2} \;. \end{aligned}$$

Since $|k| < R$ for all $k \in \Gamma ^{\text{ nor }}$, we conclude, with the bounds (9.10), that

$$\begin{aligned} \Big | \sum _{k \in \Gamma ^{\text{ nor }}} 2\hbar \kappa |k| \sum _{\alpha ,\beta \in \mathcal {I}_k} \left[ \widetilde{O}^T(k) O^T (k) \mathfrak {K} (k) \right] _{\alpha ,\beta } \langle \eta , c_\alpha ^* (k) \mathfrak {F}_\beta (1,k) \eta \rangle \Big | \leqq C N^{-2/3+ 2\delta } R^4 M^{2} \;. \end{aligned}$$

Proceeding similarly to bound the last two terms on the right-hand side of (9.15), we obtain

$$\begin{aligned} \begin{aligned}&\sum _{k \in \Gamma ^{\text{ nor }}} 2\hbar \kappa |k| \sum _{\alpha ,\beta \in \mathcal {I}_k} \mathfrak {K}_{\alpha ,\beta } (k) \langle \eta , Z^* c_\alpha ^* (k) c_\beta (k) Z \eta \rangle \\&\quad \geqq \sum _{k \in \Gamma ^{\text{ nor }}} 2\hbar \kappa |k| \sum _{\alpha ,\beta \in \mathcal {I}_k} \left[ \widetilde{O}^T(k) O^T (k) \mathfrak {K} (k) O (k) \widetilde{O}(k) \right] _{\alpha ,\beta } \langle \eta , c_\alpha ^* (k) c_\beta (k) \eta \rangle - C N^{-2/3+ 2\delta } R^4 M^{2}\,. \end{aligned} \end{aligned}$$

According to (7.10), we have $\widetilde{O}^T (k) O^T (k) \mathfrak {K} (k) O (k) \widetilde{O} (k) = \widetilde{P} (k)$, with the matrix $\widetilde{P}$ defined as in (7.9). From $P \geqq D$ (and recalling from (6.4) and (6.5) the relation between $\mathbb {D}_\text{ B }$ and *D*), we get the key lower bound

$$\begin{aligned} \sum _{k \in \Gamma ^{\text{ nor }}} 2\hbar \kappa |k| \sum _{\alpha ,\beta \in \mathcal {I}_k} \mathfrak {K}_{\alpha ,\beta } (k) \langle \eta , Z^* c_\alpha ^* (k) c_\beta (k) Z \eta \rangle \geqq \langle \eta , \mathbb {D}_\text{ B } \eta \rangle - C \hbar N^{-1/3+2\delta } R^4 M^2\;. \end{aligned}$$

From (9.14), we obtain

9.16 $$\begin{aligned} \sum _{k \in \Gamma ^{\text { nor }}} 2\hbar \kappa |k| \langle \xi , h_\text{ eff } (k) \xi \rangle&\geqq E_N^\text { RPA } + \langle \eta , \mathbb {D}_\text { B } \eta \rangle \nonumber \\ {}&\quad - C \hbar \Big ( R^2 M^{1/4} N^{-1/6+\delta /2} + N^{-\delta /2} + M^{-1/4} N^{\delta /2} \nonumber \\ {}&\qquad \quad + M^{2} R^4 N^{-1/3+2\delta } + M^{3} R^4 N^{-2/3 + 2\delta } \Big ) \;. \end{aligned}$$

Inserting the last equation and (9.12) in (9.9), we find that

$$\begin{aligned} \begin{aligned} \langle \xi , (\mathbb {H}_0 + Q_\text { B}^R) \xi \rangle&\geqq E^\text { RPA}_N + \langle \eta , \mathbb {H}_0 \eta \rangle \\ {}&\quad - C\hbar \Big ( M^{-1/2} N^{\delta } + R^2 M^{1/4} N^{-1/6+\delta /2} + N^{-\delta /2} + M^{-1/4} N^{\delta /2} \\ {}&\qquad \qquad + M^{2} R^4 N^{-1/3+2\delta } + M^{3} R^4 N^{-2/3 + 2\delta } \Big )\;. \end{aligned} \end{aligned}$$

Since $\mathbb {H}_0 \geqq 0$, from (9.8) we obtain

$$\begin{aligned} \begin{aligned} \langle \xi , \mathcal {H}_\text { corr } \xi \rangle \geqq \;&E^\text { RPA}_N - C\hbar \Big ( N^{-\varepsilon /4} + N^{- (1-\gamma )/3 + 5\varepsilon /4} + N^{-\delta /2} + R^{2} M^{1/4} N^{-1/6+\delta /2} + R^{-1 /2} \\ {}&\qquad \qquad +M^{-1/2} N^{\delta } + M^{-1/4} N^{\delta /2} + M^{2} R^4 N^{-1/3+2\delta } + M^{3} R^4 N^{-2/3 + 2\delta } \Big ) \,. \end{aligned} \end{aligned}$$

Choosing $R= N^{\delta }$, $M = N^{C \delta }$ for a sufficiently large constant $C > 0$, $\gamma < 1$ and then both $\varepsilon > 0$ and $\delta > 0$ small enough, we conclude that $\langle \xi , \mathcal {H}_\text{ corr } \xi \rangle \geqq E_N^\text{ RPA } - C N^{-1/3 - \alpha }$ for some $\alpha > 0$ and thus, from (9.5), also that $\langle \xi _\text {gs}, \mathcal {H}_\text{ corr } \xi _\text {gs} \rangle \geqq E_N^\text{ RPA } - C N^{-1/3 - \alpha }$. This completes the proof of the lower bound for Theorem 1.1.

*Upper bound* Instead of working with the state $\xi = T Z \eta$ and establishing its properties through a-priori estimates, we directly use the trial state $\xi _\text{ trial } := T \Omega$, where the transformation *Z* is not needed. We compute explicitly the expectation value

$$\begin{aligned} \langle \xi _\text{ trial }, \mathcal {H}_\text{ corr } \xi _\text{ trial } \rangle = \langle \xi _\text{ trial }, (\mathbb {H}_0 + Q_\text{ B }+ \mathcal {E}_1 + \mathcal {E}_2 + \mathbb {X}) \xi _\text{ trial } \rangle \;. \end{aligned}$$

Note that by Lemma 7.3 we have

9.17 $$\begin{aligned} \langle T \Omega , \mathcal {N}^k T \Omega \rangle \leqq C_k\;, \quad \text{ for } k \in \mathbb {N}\;. \end{aligned}$$

Furthermore, for all $\delta > 0$, we have the simple bound for the gapped number operator

9.18 $$\begin{aligned} \mathcal {N}_\delta \leqq \mathcal {N}\;, \end{aligned}$$

so that all expectations values of powers of $\mathcal {N}$ and $\mathcal {N}_\delta$ in $T\Omega$ are of order one with respect to *N*. By Lemma 8.2 we get

$$\begin{aligned} \langle T\Omega , \mathbb {H}_0 T\Omega \rangle&= \langle T\Omega , (\mathbb {H}_0 - \mathbb {D}_\text{ B}) T\Omega \rangle + \langle T\Omega , \mathbb {D}_\text{ B }T\Omega \rangle \\&\leqq \langle T\Omega , \mathbb {D}_\text{ B }T\Omega \rangle + C \hbar \left( M^{-1/2} + R^3 M N^{-2/3+\delta } \right) \;. \end{aligned}$$

The expectation value $\langle T\Omega ,\mathbb {D}_\text{ B }T\Omega \rangle$ can be computed by applying the approximate Bogoliubov transform according to Lemma 7.4. Expressions that are normal-ordered in terms of bosonic pairs operators vanish on $\Omega$; only the contribution of the form $c c^*$ is non-vanishing but easily seen to be of order $\hbar$. We conclude that

9.19 $$\begin{aligned} \langle T\Omega , \mathbb {H}_0 T\Omega \rangle \leqq C \hbar \;.\end{aligned}$$

The bounds (9.17), (9.18), and (9.19) are sufficient to control all error terms in the following computation. In fact, using Lemma 4.7 and Corollary 4.9 the contributions of $\mathcal {E}_1$, $\mathcal {E}_2$, and $\mathbb {X}$ are now found to be of order $N^{-1/3-\alpha }$ for some $\alpha > 0$. Furthermore, by Lemma 6.1, we can replace $Q_\text{ B }$ by the patch-decomposed $Q_\text{ B}^R$ at the cost of a only a further small error.

It remains to compute explicitly the expectation value

$$\begin{aligned} \begin{aligned} \langle T \Omega , ( \mathbb {D}_\text{ B }+ Q_\text{ B}^R ) T\Omega \rangle = \;&\sum _{k \in \Gamma ^{\text{ nor }}} 2\hbar \kappa |k| \langle T\Omega , h_\text {eff} (k) T\Omega \rangle \leqq E_N^\text{ RPA } + C N^{-1/3-\alpha } \end{aligned} \end{aligned}$$

for $\alpha > 0$ small enough. Here, we proceeded as in (9.13) (with $Z\eta$ replaced by $\Omega$) to implement the action of the approximate Bogoliubov transformation *T* and used that all pair annihilation operators vanish on $\Omega$. This completes the proof of the upper bound for Theorem 1.1. $\square$

We quickly discuss how to adapt the computation of [4] of the explicit RPA formula. The only new aspect here is the additional factor $R^2$ in the first error term.

### Lemma 9.2

(Explicit RPA formula) Let $\Vert \hat{V} \Vert _1 < \infty$. Then

$$\begin{aligned}&\sum _{k \in \Gamma ^{\text{ nor }}}\!\!\! \hbar \kappa |k|{\text {tr}}\left( E(k) - D(k) - W(k) \right) \\&\quad = E^\text{ RPA}_N + \mathcal {O}\left( \hbar \big ( R^2 M^{1/4} N^{-1/6+\delta /2} + N^{-\delta /2} + M^{-1/4} N^{\delta /2}\big )\right) \,. \end{aligned}$$

### Proof

The proof was given in [4, Eqs. (5.13)–(5.18)] under the assumption that $\hat{V}$ has compact support. We only give the generalization of the main estimates in original notation. With a factor $|k |^2 < R^2$ (for $k\in \Gamma ^{\text{ nor }}$) originating from (5.3) we find

$$\begin{aligned} |\log f(\lambda ) - \log \tilde{f}(\lambda ) |\leqq C \left( R^2 \hat{V}(k) \sqrt{M} N^{-1/3 + \delta } + N^{-\delta } + \frac{N^\delta }{\sqrt{M}} \right) \,. \end{aligned}$$

Furthermore,

$$\begin{aligned} |\log f(\lambda )|&\leqq C \hat{V}(k) \lambda ^{-2}\;,&|\log \tilde{f}(\lambda )|&\leqq C \hat{V}(k) \lambda ^{-2}\;. \end{aligned}$$

Following [4, Eq. (5.18)] and using $\Vert \hat{V} \Vert _1 < \infty$ the proof is completed as before. $\square$

## Data Availability

Data sharing not applicable to this article as no datasets were generated or analysed during the current study.

## A Generalized Upper Bound

As an upper bound, the estimate (1.4) for the correlation energy holds under weaker assumptions on the interaction.

### Theorem A.1

(Generalized RPA upper bound) Suppose $V : \mathbb {T}^{3} \rightarrow \mathbb {R}$, $\hat{V} \geqq 0$, and

A.1 $$\begin{aligned} \sum _{k \in \mathbb {Z}^3} |k| \hat{V} (k)^2 < \infty \;. \end{aligned}$$

For $k_\text{ F }> 0$ let $N := |B_\text{ F }| = | \{ k \in \mathbb {Z}^3 : |k|\leqq k_\text{ F }\}|$. Then, as $k_\text{ F }\rightarrow \infty$, we have

A.2 $$\begin{aligned} E_N \leqq E_N^\text{ HF } + E_N^\text{ RPA } + o \, (\hbar ) \end{aligned}$$

with $E_N^\text{ RPA }$ as defined in (1.5).

### Remark

Expanding the logarithm, it is easy to check that the assumption (A.1) guarantees that the sum defining $E_N^\text{ RPA }$ in (1.5) is finite.

### Proof of Theorem A.1

We now give the proof of Theorem A.1, explaining how to generalize the argument presented in Sect. 9 in the paragraph devoted to the upper bound. For given $0 < R \ll N^{1/3}$, we consider the set $\Gamma ^\text{ nor }$, defined in (5.5). Note that in particular $\Gamma ^{\text{ nor }}$ restricts our attention to momenta $|k|<R$. Moreover, for $\delta > 0$ sufficiently small, we introduce the sets $\mathcal {I}_k^{\pm }$ and $\mathcal {I}_k = \mathcal {I}_k^+ \cup \mathcal {I}_k^-$ as in (5.1). For $k \in \Gamma ^\text{ nor }$, we define the $|\mathcal {I}_k | \times |\mathcal {I}_k|$ matrix *K*(*k*) as in Sect. 7. As stated in Lemma 7.1, we have pointwise in $k \in \Gamma ^\text{ nor }$, without using the assumption on $\hat{V}$, the bound

A.3 $$\begin{aligned} |K_{\alpha , \beta } (k) | \leqq C \frac{\hat{V} (k)}{M} \;. \end{aligned}$$

With the matrices *K*(*k*) we define the unitary operators *T* as in (7.11). In fact, it will again be useful to consider, more generally, the family of operators $T_\lambda$, for $\lambda \in [0,1]$, as introduced in (7.39), with $T_1 = T$ and $T_0 = 1$.

We define the trial state $\psi ^\text{ trial } := R_\text{ F }T \Omega \in L^2_\text{ a } (\mathbb {T}^{3N})$ and the corresponding excitation vector $\xi ^\text{ trial } := R_\text{ F}^* \psi ^\text{ trial } = T \Omega \in \chi (\mathcal {N}_\text{ h } - \mathcal {N}_\text{ p } = 0) \mathcal {F}$. Since $R_\text{ F }$ and *T* only create particles with momentum at distance smaller than *R* from the Fermi surface, and since we assumed $R \ll N^{1/3}$, we have

$$\begin{aligned} \langle \psi ^\text{ trial } , \mathcal {H}_N \psi ^\text{ trial } \rangle = \langle \psi ^\text{ trial } , \widetilde{\mathcal {H}}_N \psi ^\text{ trial } \rangle \end{aligned}$$

where $\widetilde{\mathcal {H}}_N$ is the Hamilton operator (2.2), with $\hat{V} (k)$ replaced by $\hat{V} (k) \chi (|k| \leqq CN^{1/3})$. Proceeding as in Sect. 2, we find that

A.4 $$\begin{aligned} \langle \psi ^\text{ trial } , \widetilde{\mathcal {H}}_N \psi ^\text{ trial } \rangle = E_N^\text {HF} + \langle \xi ^\text{ trial } \widetilde{\mathcal {H}}_\text{ corr } \xi ^\text{ trial } \rangle \end{aligned}$$

with the Hartree–Fock energy (1.3) (replacing $\hat{V} (k)$ with $\hat{V} (k) \chi (|k| \leqq C N^{1/3})$ does not change the right-hand side of (1.3) if $C > 0$ is large enough) and with

$$\begin{aligned} \widetilde{\mathcal {H}}_\text{ corr } = \mathbb {H}_0 + \widetilde{Q}_B + \widetilde{\mathcal {E}}_1 + \widetilde{\mathcal {E}}_2 + \widetilde{\mathbb {X}} \end{aligned}$$

where $\widetilde{Q}_B$, $\widetilde{\mathcal {E}}_1$, $\widetilde{\mathcal {E}}_2$, $\widetilde{\mathbb {X}}$ denote the operators $Q_B$, $\mathcal {E}_1$, $\mathcal {E}_2$, $\mathbb {X}$, respectively, from (2.5) and (2.6), with $\hat{V} (k)$ replaced by $\widetilde{V} (k) \chi (|k| \leqq C N^{1/3})$.

To estimate the expectation of $\widetilde{\mathcal {H}}_\text{ corr }$ in the state $\xi ^\text{ trial }$, we first establish rough bounds on the number of particles and the energy of $\xi ^\text{ trial }$.

### Lemma A.2

(Bounds for particle number and kinetic energy) For every $R > 0$ and $m \in \mathbb {N}$ there exists $C_{R,m} > 0$ such that

A.5 $$\begin{aligned} \langle T_\lambda \Omega , \mathcal {N}^m T_\lambda \Omega \rangle \leqq C_{R,m} \qquad \text {for all}\, \lambda \in [0,1]. \end{aligned}$$

Moreover, for every $R > 0$ there exists a constant $C_R < \infty$ such that

A.6 $$\begin{aligned} \langle T_\lambda \Omega , \mathbb {H}_0 T_\lambda \Omega \rangle \leqq C_R \hbar \qquad \text {for all}\, \lambda \in [0,1]. \end{aligned}$$

### Proof of Lemma A.2

For (A.5) we can proceed as in the proof of [4, Prop. 4.6]. The only new aspect is that we use the assumption (A.1) together with (A.3) to estimate

A.7 $$\begin{aligned} \sum _{k \in \Gamma ^\text{ nor }} \Vert K (k) \Vert _\text{ HS } \leqq C \sum _{|k| \leqq R} \hat{V} (k) \leqq C R \left( \sum _{|k| < R} \hat{V} (k)^2 |k| \right) ^{1/2} \leqq C R. \end{aligned}$$

This allows us to show that

$$\begin{aligned} \left| \frac{{\text{ d }}}{{\text{ d }}\lambda } \langle T_\lambda \Omega , (\mathcal {N}+5)^m T_\lambda \Omega \rangle \right| \leqq C R \langle T_\lambda \Omega , (\mathcal {N}+5)^m T_\lambda \Omega \rangle \;. \end{aligned}$$

By Grönwall’s lemma, we conclude that

$$\begin{aligned} \langle T_\lambda \Omega , \mathcal {N}^m T_\lambda \Omega \rangle \leqq e^{C_mR \lambda } \;. \end{aligned}$$

To show (A.6) we write

A.8 $$\begin{aligned} \langle T_\lambda \Omega , \mathbb {H}_0 T_\lambda \Omega \rangle = \langle T_\lambda \Omega , (\mathbb {H}_0 - \mathbb {D}_\text{ B}) T_\lambda \Omega \rangle + \langle T_\lambda \Omega , \mathbb {D}_\text{ B } T_\lambda \Omega \rangle \end{aligned}$$

with the operator $\mathbb {D}_\text{ B }$ introduced in (6.3). From Lemma 8.2 and (A.5), we find

A.9 $$\begin{aligned} | \langle T_\lambda \Omega , (\mathbb {H}_0 - \mathbb {D}_\text{ B}) T_\lambda \Omega \rangle | \leqq C_R \hbar \big ( M^{-1/2} + M N^{-2/3+\delta } \big ) \;. \end{aligned}$$

As in the proof of (A.5) above, the condition $\sum _{k \in \mathbb {Z}^3} \hat{V} (k) (1+ |k|) < \infty$ required in Lemma 8.2 is now replaced (since $K (k) = 0$ for $|k| > R$) by

$$\begin{aligned} \sum _{|k| < R} \hat{V} (k) (1+ |k|) \leqq C R^2 \left( \sum _k \hat{V} (k)^2 |k| \right) ^{1/2} \leqq C R^2 \end{aligned}$$

which leads (together with (A.5)) to an *R*-dependent constant in (A.9). We also have

A.10 $$\begin{aligned} \langle T_\lambda \Omega , \mathbb {D}_\text{ B } T_\lambda \Omega \rangle&\leqq C_R \hbar \sum _{k \in \Gamma ^{\text {nor}}} \sum _{\alpha = 1}^{M} \langle T_{\lambda } \Omega , c^{*}_{\alpha }(k) c_{\alpha }(k) T_{\lambda } \Omega \rangle \nonumber \\ {}&\leqq C_{R} \hbar \langle T_{\lambda } \Omega , \mathcal {N} T_{\lambda } \Omega \rangle \leqq C_{R} \hbar \;, \end{aligned}$$

where we used (5.9) in the second and (A.5) in the third inequality. Inserting (A.9) and (A.10) in (A.8), we obtain (A.6). This concludes the proof of Lemma A.2. $\square$

To estimate the potential energy we need the following lemma, which shows that, when computing expectation values in $\xi ^\text{ trial }$, we can effectively cutoff the interaction $\hat{V}$ to momenta $|k| \leqq R$, up to negligible errors. This observation relies on the fact that *T* only creates particle–hole pairs with pair momentum $|k|\leqq R$.

### Lemma A.3

(Control of the high-momentum cutoff) Assume $\sum _{k \in \mathbb {Z}^3} |k| \hat{V} (k)^2 < \infty$. Then for every $R > 0$ there exists $C_R > 0$ such that

A.11 $$\begin{aligned} \begin{aligned} \frac{1}{N} \sum _{k \in \mathbb {Z}^3 : R< |k| \leqq C N^{1/3}} \hat{V} (k) \langle T \Omega , b^* (k) b (k) T \Omega \rangle \leqq C_R M^{3/2} N^{-1/2 + \delta /2}\;, \\ \Big | \frac{1}{N} \sum _{k \in \mathbb {Z}^3 : R < |k| \leqq C N^{1/3}} \hat{V} (k) \langle T \Omega , b (k) b (-k) T \Omega \rangle \Big | \leqq C_R M^{3/2} N^{-1/2 + \delta /2} \;. \end{aligned} \end{aligned}$$

### Proof of Lemma A.3

Consider the second inequality in (A.11). We write

A.12 $$\begin{aligned}&\frac{1}{N} \sum _{R< |k| \leqq C N^{1/3}} \hat{V} (k) \langle T\Omega , b (k) b (-k) T \Omega \rangle \nonumber \\&\quad = \frac{1}{N} \sum _{R < |k| \leqq C N^{1/3}} \hat{V} (k) \sum _{k' \in \Gamma ^\text{ nor }} \sum _{\alpha , \beta \in \mathcal {I}_{k'}} K_{\alpha ,\beta } (k') \int _0^1 {\text{ d }}\lambda \, \Big \langle T_\lambda \Omega , \Big [ c_\alpha ^* (k') c^*_\beta (k') , b (k) b (-k) \Big ] T_\lambda \Omega \Big \rangle \;. \end{aligned}$$

We compute that

A.13 $$\begin{aligned} \begin{aligned}&\Big [ c_\alpha ^* (k') c^*_\beta (k') , b (k) b (-k) \Big ] \\ {}&\quad = c^{*}_{\alpha }(k') b(k) [ c^{*}_{\beta }(k'), b(-k) ] + c^{*}_{\alpha }(k') [ c^{*}_{\beta }(k'), b(k) ] b(-k) \\ {}&\qquad + b(k) [ c^{*}_{\alpha }(k') , b(-k) ] c^{*}_{\beta }(k') + [ c^{*}_{\alpha }(k') , b(k) ] b(-k) c^{*}_{\beta }(k')\;.\end{aligned} \end{aligned}$$

We consider the case $\alpha , \beta \in \mathcal {I}^{+}_{k'}$ (so that $c_\alpha ^* (k') = b_\alpha ^* (k')$ and $c_\beta ^* (k') = b_\beta ^* (k')$ by (5.4)); the other cases can be studied in the same way. We find that

A.14 $$\begin{aligned}&{[} c^{*}_{\alpha }(k'), b(k) ] \\ {}&=\frac{1}{n_{\alpha }(k')} \sum _{\begin{array}{c} p \in B_F^c \cap B_\alpha : \\ p-k' \in B_F \cap B_\alpha \end{array}} \sum _{q \in B_F^c \cap B_F + k} \big ( \delta _{p,q} \delta _{k,k'} -\delta _{p,q} a_{p-k'}^* a_{q-k} - \delta _{p-k', q-k} a_p^* a_q \big )\;. \end{aligned}$$

Thanks to the constraint $|k'|< R < |k|$, the otherwise dominant contribution due to $\delta _{p,q} \delta _{k,k'}$ vanishes. For such *k* and $k'$ and for any $\psi , \varphi \in \mathcal {F}$ we obtain

A.15 $$\begin{aligned} |\langle \varphi , [ c^{*}_{\alpha }(k'), b(k) ] \psi \rangle | \leqq \frac{C}{n_{\alpha }(k')} \Vert \mathcal {N}^{1/2} \varphi \Vert \Vert \mathcal {N}^{1/2} \psi \Vert \;. \end{aligned}$$

We can use this estimate to bound all the contributions to (A.12) arising from the various terms in the right-hand side of (A.13). For instance, consider the first. Using (A.14) we have

$$\begin{aligned} | \langle T_{\lambda } \Omega , c^{*}_{\alpha }(k') b(k) [ c^{*}_{\beta }(k'), b(-k) ] T_{\lambda } \Omega \rangle |&\leqq \frac{C}{n_{\beta }(k')} \Vert \mathcal {N}^{1/2} b^{*}(k) c_{\alpha }(k') T_{\lambda } \Omega \Vert \Vert \mathcal {N}^{1/2} T_{\lambda } \Omega \Vert \\&= \frac{C}{n_{\beta }(k')} \Vert b^{*}(k) c_{\alpha }(k') \mathcal {N}^{1/2}T_{\lambda } \Omega \Vert \Vert \mathcal {N}^{1/2} T_{\lambda } \Omega \Vert . \end{aligned}$$

Lemma 5.1, together with the assumption $\alpha , \beta \in \mathcal {I}_{k'}$, implies that $n_{\beta } (k') \geqq C N^{1/3-\delta /2} M^{-1/2}$. Next, we will use the bounds

A.16 $$\begin{aligned} \Vert b^{\natural }(k) \varphi \Vert \leqq C|k|^{1/2} N^{1/3} \Vert (\mathcal {N} + 1)^{1/2} \varphi \Vert \;,\qquad \Vert c^{\natural }_{\alpha }(k') \varphi \Vert \leqq C\Vert (\mathcal {N}+1)^{1/2} \varphi \Vert \;, \end{aligned}$$

where $b^\natural$ is either *b* or $b^*$, and analogously for $c^\natural$. Here, the first estimate follows from [6, Eqs. (4.12) and (4.13)] (observing that $|B_F^c \cap B_F + k| \leqq C |k| N^{2/3}$), the second from Lemma 5.3 (using the inequality $[c_\alpha (k'), c_\alpha ^* (k')] \leqq 1$; see [5, Eq. (5.10)]). Thus

A.17 $$\begin{aligned} | \langle T_{\lambda } \Omega , c^{*}_{\alpha }(k') b(k) [ c^{*}_{\beta }(k'), b(-k) ] T_{\lambda } \Omega \rangle | \leqq C|k|^{1/2} N^{\delta /2} M^{1/2} \langle T_{\lambda } \Omega , (\mathcal {N} + 1)^{3} T_{\lambda } \Omega \rangle \;. \end{aligned}$$

All the other contributions in (A.13) can be estimated in a similar way. We get, using the bounds $|K_{\alpha ,\beta }(k')| \leqq {\hat{V}}(k')/M$ and (A.5),

A.18 $$\begin{aligned} \begin{aligned}&\Big | \frac{1}{N} \sum _{k \in \mathbb {Z}^3 : R< |k| \leqq C N^{1/3}} \hat{V} (k) \langle T \Omega , b(k) b(-k) T \Omega \rangle \Big | \\&\quad \leqq C_{R} M^{3/2} N^{-1 + \delta /2} \sum _{R < |k| \leqq C N^{1/3}} |k|^{1/2}\hat{V} (k) \sum _{k' \in \Gamma ^\text{ nor }} {\hat{V}}(k') \\&\quad \leqq C_{R} M^{3/2} N^{-1/2 + \delta /2} \end{aligned} \end{aligned}$$

where the sum over $k'$ has been absorbed in the constant $C_R$ (recall that $|k'| < R$ in $\Gamma ^\text{ nor }$) and where we estimated

A.19 $$\begin{aligned} \sum _{k: |k| \leqq CN^{1/3}} |k|^{1/2}\hat{V} (k) \leqq CN^{1/2} \left( \sum _{k} |k| {\hat{V}}(k)^{2}\right) ^{1/2} \leqq CN^{1/2}\; . \end{aligned}$$

This concludes the proof of the second inequality in (A.11). The first can be shown similarly; we omit the details. This concludes the proof of Lemma A.3. $\square$

With Lemma A.2 and Lemma A.3, we can go back to the computation of the expectation value on the right-hand side of (A.4). We control the expectation of the error term $\widetilde{\mathcal {E}}_1$ with the bound

$$\begin{aligned} \Vert \widetilde{\mathcal {E}}_1 \xi \Vert \leqq \frac{C \Vert \hat{V} \Vert _1}{N} \Vert \mathcal {N}^2 \xi \Vert \end{aligned}$$

established in [6, Eq. (4.10)]. With (A.5) and estimating

$$\begin{aligned} \sum _{|k| \leqq C N^{1/3}} \hat{V} (k) \leqq C N^{1/3} \Big (\sum _{k \in \mathbb {Z}^3} \hat{V} (k)^2 |k| \Big )^{1/2} \leqq C N^{1/3}, \end{aligned}$$

we find, for a constant $C_R$ depending on the cutoff $R > 0$, that

$$\begin{aligned} \langle \xi ^\text{ trial }, \widetilde{\mathcal {E}}_1 \xi ^\text{ trial } \rangle \leqq C_R N^{-2/3}\;. \end{aligned}$$

The expectation value of $\widetilde{\mathcal {E}}_2$ in our trial state vanishes for parity reasons exactly as in [4, Lemma 5.2].

Applying Lemma A.3 and (A.11) and using the fact that $\mathbb {X}\leqq 0$, from (A.4) we get

$$\begin{aligned} \langle \psi ^\text{ trial } , \mathcal {H}_N \psi ^\text{ trial } \rangle \leqq E_N^\text {HF} + \langle \xi ^\text{ trial }, (\mathbb {H}_0 + \widetilde{Q}_B^R) \xi ^\text{ trial } \rangle + C_R N^{-2/3} + C_R M^{3/2} N^{-1/2 + \delta /2} \end{aligned}$$

where we defined

$$\begin{aligned} \widetilde{Q}_B^R := \frac{1}{N} \sum _{k \in \mathbb {R}^3 : |k| \leqq R} \hat{V} (k) \Big ( b^* (k) b(k) + \frac{1}{2} \big (b^* (k) b^* (-k) + b(k) b(-k) \big ) \Big ) \;. \end{aligned}$$

In order to obtain an upper bound for the expectation of the operator $\mathbb {H}_0 + \widetilde{Q}_B^R$, we proceed as in the proof of Theorem 1.1, now with $\hat{V} (k)$ replaced everywhere by $\hat{V} (k) \chi (|k| \leqq R)$. We conclude that

$$\begin{aligned} \begin{aligned}&\langle \psi ^\text{ trial } , \mathcal {H}_N \psi ^\text{ trial } \rangle \\&\quad \leqq E_N^\text {HF} + \hbar \kappa _0 \sum _{|k| \leqq R} |k| \left( \frac{1}{\pi } \int _0^\infty \log \left( 1 + 2\pi \kappa _0 \hat{V} (k) \Big (1-\lambda \arctan \big (\frac{1}{\lambda }\big ) \Big ) \right) {\text{ d }}\lambda - \frac{\pi }{2} \kappa _0 \hat{V} (k) \right) \\&\qquad + C_R N^{-2/3} + C_R N^{-1/3} M^{-1/2} + C_R M^{3/2} N^{-1/2+\delta /2} \\&\quad \leqq E_N^\text {HF} + E^\text {RPA}_N + C \sum _{|k| > R} \hat{V} (k)^2 |k| + C_R \Big ( N^{-2/3} + N^{-1/3} M^{-1/2} + M^{3/2} N^{-1/2 + \delta /2} \Big )\;. \end{aligned} \end{aligned}$$

Fixing $M = N^\alpha$, choosing $\alpha > 0$ small enough and then $R = R(N)$ so that $R(N) \rightarrow \infty$ as $N \rightarrow \infty$ at a sufficiently slow pace, we obtain (A.2). This concludes the proof of the generalized RPA upper bound, Theorem A.1. $\square$

## Footnotes

1 By *approximate* bosonization we refer to the fact that we construct operators that only up to an error term satisfy canonical commutator relations; this is in contrast to certain one-dimensional fermionic systems [24] and spin systems [2, 11, 12, 26] which can be expressed through operators that satisfy the canonical commutator relations exactly.

2 The transformation *Z* is a trivial Bogoliubov transformation, corresponding to only a change of basis in the one-boson Hilbert space. In the language of bosonic second-quantized operators, it corresponds to a transformation of the form $e^{{\text{ d }}\Gamma (L)} = \Gamma (e^L)$, where $e^L$ is an orthogonal matrix acting on the one-boson space.
